# Supplementary material for: Discovery and characterization of a terpene biosynthetic pathway featuring a norbornene-forming Diels-Alderase
Source: Nat Commun. 2022 May 11;13:2568. doi: 10.1038/s41467-022-30288-6 (PMC9095873; doi:10.1038/s41467-022-30288-6)

Supplementary Information

Discovery and Characterization of a Terpene Biosynthetic Pathway featuring a Norbornene-forming Diels-Alderase

Zuodong Sun[1], Cooper S. Jamieson[2], Masao Ohashi[1], K. N. Houk[1,2]*, and Yi Tang[1,2]*

[1]Department of Chemical and Biomolecular Engineering, University of California, Los Angeles, Los Angeles, California 90095, United States

[2]Department of Chemistry and Biochemistry, University of California, Los Angeles, Los Angeles, California 90095, United States

* Emails of corresponding authors: houk@chem/ucla.edu; yitang@g.ucla.edu

Table of Contents

I Supplementary Notes 6
Isolation of compounds 6
Chemical synthesis of compounds 4, 7, and **10** 8
Preparation of compound 9 9
II Supplementary Tables 10
Supplementary Table 1. Annotation of the putative sordaricin B cluster. 10
Supplementary Table 2. DNA and protein sequence of SdnG (*S. araneosa*) used in this study. 11
Supplementary Table 3. Spectroscopic data of compound **1**. 12
Supplementary Table 4. Spectroscopic data of compound **2**. 13
Supplementary Table 5. Spectroscopic data of compound **3** 14
Supplementary Table 6. Spectroscopic data of compound **4**. 15
Supplementary Table 7. Spectroscopic data of compound **5** 16
Supplementary Table 8. Spectroscopic data of compound **6**. 17
Supplementary Table 9. Spectroscopic data of compound **7**. 18
Supplementary Table 10. Spectroscopic data of compound **8**. 19
Supplementary Table 11. Spectroscopic data of compound **9**. 20
Supplementary Table 12. Spectroscopic data of compound **10**. 21
Supplementary Table 13. Spectroscopic data of compound **11**. 22
Supplementary Table 14. Spectroscopic data of compound **12**. 23
Supplementary Table 15. Spectroscopic data of compound **13**. 24
Supplementary Table 16. Spectroscopic data of compound **14**. 25
III Supplementary Figures 26
Supplementary Fig 1. Proposed biosynthesis of sordaricin, sordarin, and hypoxysordarin by Kudo, et al[19]. 26
Supplementary Fig 2. GC-MS analysis of *A. nidulans* transformed with SdnA-C.. 27
Supplementary Fig 3. Stepwise reconstitution of genes involved in sordaricin biosynthesis. 28
Supplementary Fig 4. UV-vis spectra of purified compounds. 29
Supplementary Fig 5. Proposed mechanism of SdnB (diol cleavage[5]) and SdnH (desaturation[6]). 30
Supplementary Fig 6. Reconstitution of sordaricin biosynthesis in *S. cerevisiae* RC01. 31
Supplementary Fig 7. Blast research results for SdnG homologs in NCBI genomic database. 32
Supplementary Fig 8. Characterization of SdnG. 33

Supplementary Fig 9. ^{1}H NMR of compound **1** in d5-pyridine, 500 MHz. 34
Supplementary Fig 10. ^{13}C NMR of compound **1** in d5-pyridine, 500 MHz. 34
Supplementary Fig 11. ^{1}H-^{1}H COSY of compound **1** in d5-pyridine, 500 MHz. 35
Supplementary Fig 12. ^{1}H-^{13}C HSQC of compound **1** in d5-pyridine, 500 MHz. 35
Supplementary Fig 13. ^{1}H-^{13}C HMBC of compound **1** in d5-pyridine, 500 MHz. 36
Supplementary Fig 14. ^{1}H NMR of compound **2** in $CDCl_3$, 500 MHz. 36
Supplementary Fig 15. ^{13}C NMR of compound **2** in $CDCl_3$, 500 MHz. 37
Supplementary Fig 16. ^{1}H-^{1}H COSY of compound **2** in $CDCl_3$, 500 MHz. 37
Supplementary Fig 17. ^{1}H-^{13}C HSQC of compound **2** in $CDCl_3$, 500 MHz. 38
Supplementary Fig 18. ^{1}H-^{13}C HMBC of compound **2** in $CDCl_3$, 500 MHz. 38
Supplementary Fig 19. ^{1}H-^{1}H NOESY of compound **2** in $CDCl_3$, 500 MHz. 39
Supplementary Fig 20. ^{1}H NMR of compound **3** in $CDCl_3$, 500 MHz. 39
Supplementary Fig 21. ^{13}C NMR of compound **3** in $CDCl_3$, 500 MHz. 40
Supplementary Fig 22. ^{1}H-^{1}H COSY of compound **3** in $CDCl_3$, 500 MHz. 40
Supplementary Fig 23. ^{1}H-^{13}C HSQC of compound **3** in $CDCl_3$, 500 MHz. 41
Supplementary Fig 24. ^{1}H-^{13}C HMBC of compound **3** in $CDCl_3$, 500 MHz. 41
Supplementary Fig 25. ^{1}H NMR of compound **4** in CD_2Cl_2, 500 MHz. 42
Supplementary Fig 26. ^{13}C NMR of compound **4** in CD_2Cl_2, 500 MHz. 42
Supplementary Fig 27. ^{1}H-^{1}H COSY of compound **4** in CD_2Cl_2, 500 MHz. 43
Supplementary Fig 28. ^{1}H-^{13}C HSQC of compound **4** in CD_2Cl_2, 500 MHz. 43
Supplementary Fig 29. ^{1}H-^{13}C HMBC of compound **4** in CD_2Cl_2, 500 MHz. 44
Supplementary Fig 30. ^{1}H NMR of compound **5** in d5-pyridine, 500 MHz. 44
Supplementary Fig 31. ^{13}C NMR of compound **5** in d5-pyridine, 500 MHz. 45
Supplementary Fig 32. ^{1}H-^{1}H COSY of compound **5** in d5-pyridine, 500 MHz. 45
Supplementary Fig 33. ^{1}H-^{13}C HSQC of compound **5** in d5-pyridine, 500 MHz. 46
Supplementary Fig 34. ^{1}H-^{13}C HMBC of compound **5** in d5-pyridine, 500 MHz. 46
Supplementary Fig 35. ^{1}H NMR of compound **6** in d5-pyridine, 500 MHz. 47
Supplementary Fig 36. ^{13}C NMR of compound **6** in d5-pyridine, 500 MHz. 47
Supplementary Fig 37. ^{1}H-^{1}H COSY of compound **6** in d5-pyridine, 500 MHz. 48
Supplementary Fig 38. ^{1}H-^{13}C HSQC of compound **6** in d5-pyridine, 500 MHz. 48
Supplementary Fig 39. ^{1}H-^{13}C HMBC of compound **6** in d5-pyridine, 500 MHz. 49
Supplementary Fig 40. ^{1}H NMR of compound **7** in CD_2Cl_2, 500 MHz. 49
Supplementary Fig 41. ^{13}C NMR of compound **7** in CD_2Cl_2, 500 MHz. 50

Supplementary Fig 42. ^{1}H-^{1}H COSY of compound **7** in CD_2Cl_2, 500 MHz. 50
Supplementary Fig 43. ^{1}H-^{13}C HSQC of compound **7** in CD_2Cl_2, 500 MHz. 51
Supplementary Fig 44. ^{1}H-^{13}C HMQC of compound **7** in CD_2Cl_2, 500 MHz. 51
Supplementary Fig 45. ^{1}H NMR of compound **8** in $CDCl_3$, 500 MHz. 52
Supplementary Fig 46. ^{13}C NMR of compound **8** in $CDCl_3$, 500 MHz. 52
Supplementary Fig 47. ^{1}H-^{1}H COSY of compound **8** in $CDCl_3$, 500 MHz. 53
Supplementary Fig 48. ^{1}H-^{13}C HSQC of compound **8** in $CDCl_3$, 500 MHz. 53
Supplementary Fig 49. ^{1}H-^{13}C HMBC of compound **8** in $CDCl_3$, 500 MHz. 54
Supplementary Fig 50. ^{1}H-^{1}H NOESY of compound **8** in $CDCl_3$, 500 MHz. 54
Supplementary Fig 51. ^{1}H NMR of mixed compounds **7** and **9** in d5-pyridine, 500 MHz. 55
Supplementary Fig 52. ^{1}H NMR of compound **9** in $CDCD_3$, 500 MHz. 55
Supplementary Fig 53. ^{13}C NMR of compound **9** in $CDCD_3$, 500 MHz. 56
Supplementary Fig 54. ^{1}H-^{1}H COSY of compound **9** in $CDCD_3$, 500 MHz. 56
Supplementary Fig 55. ^{1}H-^{13}C HSQC of compound **9** in $CDCD_3$, 500 MHz. 57
Supplementary Fig 56. ^{1}H-^{13}C HMBC of compound **9** in $CDCD_3$, 500 MHz. 57
Supplementary Fig 57. ^{1}H NMR of compound **10** in CD_2Cl_2, 500 MHz. 58
Supplementary Fig 58. ^{13}C NMR of compound **10** in CD_2Cl_2, 500 MHz. 58
Supplementary Fig 59. ^{1}H-^{1}H COSY of compound **10** in CD_2Cl_2, 500 MHz. 59
Supplementary Fig 60. ^{1}H-^{13}C HSQC of compound **10** in CD_2Cl_2, 500 MHz. 59
Supplementary Fig 61. ^{1}H-^{13}C HMBC of compound **10** in CD_2Cl_2, 500 MHz. 60
Supplementary Fig 62. ^{1}H NMR of compound **11** in d5-pyridine, 500 MHz. 60
Supplementary Fig 63. ^{13}C NMR of compound **11** in d5-pyridine, 500 MHz. 61
Supplementary Fig 64. ^{1}H-^{1}H COSY of compound **11** in d5-pyridine, 500 MHz. 61
Supplementary Fig 65. ^{1}H-^{13}C HSQC of compound **11** in d5-pyridine, 500 MHz. 62
Supplementary Fig 66. ^{1}H-^{13}C HMBC of compound **11** in d5-pyridine, 500 MHz. 62
Supplementary Fig 67. ^{1}H NMR of compound **12** in d5-pyridine, 500 MHz. 63
Supplementary Fig 68. ^{13}C NMR of compound **12** in d5-pyridine, 500 MHz. 63
Supplementary Fig 69. ^{1}H-^{1}H COSY of compound **12** in d5-pyridine, 500 MHz. 64
Supplementary Fig 70. ^{1}H-^{13}HSQC of compound **12** in d5-pyridine, 500 MHz. 64
Supplementary Fig 71. ^{1}H-^{13}HMBC of compound **12** in d5-pyridine, 500 MHz. 65
Supplementary Fig 72. ^{1}H NMR of compound **13** in d5-pyridine, 500 MHz. 65
Supplementary Fig 73. ^{13}C NMR of compound **13** in d5-pyridine, 500 MHz. 66
Supplementary Fig 74. ^{1}H-^{1}H COSY of compound **13** in d5-pyridine, 500 MHz. 66

Supplementary Fig 75. ^{1}H-^{13}C HSQC of compound **13** in d5-pyridine, 500 MHz.............................. 67
Supplementary Fig 76. ^{1}H-^{13}C HSQC of compound **13** in d5-pyridine, 500 MHz.............................. 67
Supplementary Fig 77. ^{1}H NMR of compound **14** in d5-pyridine, 500 MHz...................................... 68
Supplementary Fig 78. ^{13}C NMR of compound **14** in d5-pyridine, 500 MHz..................................... 68
Supplementary Fig 79. ^{1}H-^{1}H COSY of compound **14** in d5-pyridine, 500 MHz............................... 69
Supplementary Fig 80. ^{1}H-^{13}C HSQC of compound **14** in d5-pyridine, 500 MHz.............................. 69
Supplementary Fig 81. ^{1}H-^{13}C HMBC of compound **14** in d5-pyridine, 500 MHz. 70
Supplementary Fig 82. Intrinsic reaction coordinate calculation initiated from TS-2.......................... 70
IV Supplementary references 71
V Uncropped scan of SDS-PAGE in Supplementary Fig 8. 71

I Supplementary Notes

Isolation of compounds

General isolation methods were outlined in the Method section of main text. The source, yield, and analytical data of each compound were listed here.

<u>Isolation of compound **1**</u>

Compound **1** was isolated from 2L of CDST agar plates inoculated with *A. nidulans* expressing SdnA-C-B-H-F-E as 40 mg reddish white solid (20 mg/L). $[\alpha]^{23}{}_{D}$ = -53.4 (c 4.2 MeOH), lit.[1] -55.3 (c 0.19 MeOH); 1H NMR (500 MHz, d5-pyridine) δ 10.26 (s, 1H), 6.16 (dd, J = 3.7, 1.3 Hz, 1H), 4.38 (d, J = 10.7 Hz, 1H), 4.23 (d, J = 10.6 Hz, 1H), 2.98 (t, J = 3.9 Hz, 1H), 2.74 (sept, J = 6.8 Hz, 1H), 2.45 (m, 1H), 2.37-2.27 (m, 2H), 2.08-1.96 (m, 4H), 1.78 (m, 1H), 1.51 (d, J = 12.4 Hz, 1H), 1.11 (t, J = 6.7 Hz, 6H), 1.05 (m, 2H), 0.84 (d, J = 6.9 Hz, 3H); 13C NMR (500 MHz, d5-pyridine) δ 204.7, 175.6, 148.6, 130.7, 73.5, 67.2, 66.6, 59.1, 46.8, 41.7, 41.6, 32.1, 31.3, 29.4, 28.9, 27.9, 26.6, 22.5, 21.0, 17.4; HRMS for $C_{20}H_{28}O_4$ (ESI, $[M+H^+]$) calculated 333.2060, found 333.2072 (deviation 3.49 ppm). The 1H and 13C NMRs of compound **1** match with those previously reported for sordaricin[1]. See Supplementary Table 3, Supplementary Figs. 4, 9-13 for more spectroscopic data.

<u>Isolation of compound **2**</u>

Compound **2** was isolated from 2L of CDST agar plates inoculated with *A. nidulans* expressing SdnA-C-B as 50 mg light yellow solid (25 mg/L). $[\alpha]^{23}{}_{D}$ = 42.5 (c 3.0 DMSO); 1H NMR (500 MHz, $CDCl_3$) δ 5.15 (t, J = 1.0 Hz, 1H), 4.93 (s, 1H), 4.47 (dt, J = 5.3, 1.9 Hz, 1H), 4.35 (d, J = 5.4 Hz, 1H), 3.15 (sept, J = 6.8 Hz, 1H), 2.13 (m, 3H), 2.03 (m, 1H), 1.91-1.77 (m, 2H), 1.67 (m, 2H), 1.62-1.51 (m, 2H), 1.39 (m, 1H), 1.36-1.26 (m, 2H), 0.97 (s, 3H), 0.94 (d, J = 6.9 Hz, 3H), 0.91 (d, J = 6.7 Hz, 3H), 0.78 (d, J = 7.1 Hz, 3H); 13C NMR (500 MHz, $CDCl_3$) δ 155.9, 146.8, 137.8, 106.6, 76.3, 69.1, 50.6, 50.4, 45.6, 43.0, 41.5, 34.6, 34.4, 34.4, 28.4, 27.3, 26.8, 21.8, 20.7, 14.7; HRMS for $C_{20}H_{32}O_2$ (ESI, $[M+H^+-H_2O]$) calculated 287.2369, found 287.2384 (deviation 5.08 ppm). See Supplementary Table 4, Supplementary Figs. 4, 14-19 for more spectroscopic data.

<u>Isolation of compound **3**</u>

Compound **3** was coisolated with compound **2** as 7 mg light yellow solid (3.5 mg/L). 1H NMR (500 MHz, $CDCl_3$) δ 9.97 (s, 1H), 5.09 (q, J = 1.5 Hz, 1H), 4.85 (s, 1H), 4.06-3.94 (m, 2H), 3.45-3.38 (sept, J = 7.0 Hz, 1H), 2.47-2.39 (m, 2H), 2.21-2.12 (m, 2H), 1.89-1.75 (m, 3H), 1.68 (dd, J = 14.1, 9.4 Hz, 1H), 1.59 (m, 1H), 1.57-1.47 (m, 2H), 1.41-1.29 (m, 2H), 1.19 (s, 3H), 1.11 (d, J = 6.9 Hz, 3H), 1.09 (d, J = 6.9 Hz, 3H), 0.87 (d, J = 7.1 Hz, 3H); 13C NMR (500 MHz, $CDCl_3$) δ 188.9, 172.8, 152.2, 142.0, 109.4, 64.6, 50.1, 48.2, 44.9, 36.1, 36.1, 35.6, 33.5, 30.1, 28.9, 26.9, 26.4, 21.7, 21.7, 16.1; HRMS for $C_{20}H_{32}O_2$ (ESI, $[M+Na^+]$) calculated 327.2295, found 327.2306 (derivation 3.51 ppm). See Supplementary Table 5, Supplementary Figs. 4, 20-24 for more spectroscopic data.

<u>Isolation of compound **5**</u>

Compound **5** was isolated from 2L of CDST agar plates inoculated with *A. nidulans* expressing SdnA-C-B-H as 14 mg light yellow solid (9 mg/L). 1H NMR (500 MHz, d5-Pyridine) δ 10.20 (s, 1H), 6.85 (d, J = 5.8 Hz, 1H), 6.49 (dd, J = 5.4 Hz, 1H), 5.55 (t, J = 1.9 Hz, 1H), 5.03 (s, 1H), 4.36 (m, 2H), 3.44 (sept, J = 7.0 Hz, 1H), 2.36 (m, 1H), 2.28 (dd, J = 13.9, 3.1 Hz, 1H), 1.98-1.85 (m, 3H), 1.68 (m, 1H), 1.54-1.48 (m, 1H), 1.37 (s, 3H), 1.29-1.20 (m, 1H), 1.14 (d, J = 6.8 Hz, 3H), 1.11 (d, J = 6.8 Hz, 3H), 0.83 (d, J = 7.0 Hz, 2H); 13C NMR (500 MHz, d5-Pyridine) δ 184.4, 170.0, 157.2, 153.8, 144.0, 128.7, 108.4, 64.5, 57.8, 47.9,

45.0, 36.4, 34.9, 33.3, 30.1, 26.4, 22.8, 22.7, 22.5, 15.5; HRMS for $C_{20}H_{30}O_2$ (ESI, [M+H$^+$]) calculated 303.2319, found 303.2325 (derivation 2.12 ppm). See Supplementary Table 7, Supplementary Figs. 4, 30-34 for more spectroscopic data.

Isolation of compound **6**

Compound **6** was coisolated with compound **5** as 9 mg light yellow solid (4.5 mg/L). 1H NMR (500 MHz, d5-Pyridine) δ 6.72 (d, J = 5.5 Hz, 1H), 6.51 (d, J = 5.4Hz, 1H), 5.54 (s, 1H), 5.16 (s, 1H), 4.46 (d, J = 14.9 Hz, 1H), 4.40 (d, J = 14.9 Hz, 1H), 4.33 (m, 1H), 2.50 (d, J = 13.9, 1H), 2.43 (m, 1H), 2.10-2.02 (m, 1H), 1.93 (m, 2H), 1.76-1.68 (m, 1H), 1.57 (s, 3H), 1.51 (m, 2H), 1.26 (m, 1H), 1.25 (d, J = 6.8 Hz, 3H), 1.19 (d, J = 6.9 Hz, 3H), 0.87 (d, J = 7.1 Hz, 3H); 13C NMR (500 MHz, d5-Pyridine) δ 167.6, 163.7, 153.9, 153.9, 136.6, 128.2, 108.3, 64.4, 58.7, 48.1, 45.2, 36.2, 35.0, 33.3, 29.8, 27.8, 23.3, 22.7, 22.3, 15.4; HRMS for $C_{20}H_{30}O_3$ (ESI, [M+H$^+$-H_2O]) calculated 301.2162, found 301.2161 (deviation 0.35 ppm). See Supplementary Table 8, Supplementary Figs. 4, 35-39 for more spectroscopic data.

Isolation of compound **8**

Plasmid XW55 harboring SdnH was transformed into *Saccharomyces cerevisiae* RC01 via Frozen-EZ Yeast Transformation II Kit (Zymo Research)[2]. The transformant was grown in 20 mL uracil drop out media (2% glucose, 0.5% casamino acid, 0.67% yeast nitrogen base w/o amino acid, 0.002% adenine, 0.002% L-tryptophan) overnight which was then used to inoculate 300 mL YPD media (1% yeast extract, 2% peptone, 2% glucose). This culture was shaken at 28 °C 250 rpm overnight before fed with 10 mg compound **2** in DMSO. The culture was left in shaker for additional two days. Cells and media were extracted separately by acetone and ethyl acetate respectively. The organics were combined and dried with a rotary evaporator. The crude extract was then separated by HPLC as stated in main methods. Compound **8** was obtained as 4 mg of light yellow solid (40% isolation yield from **2**). 1H NMR (500 MHz, $CDCl_3$) δ 6.26 (d, J = 5.4 Hz, 1H), 6.02 (d, J = 5.4, 1H), 5.21 (t, J = 1.2 Hz, 1H), 4.90 (s, 1H), 4.68 (d, J = 5.7 Hz, 1H), 4.656 (d, J = 5.7 Hz, 1H), 3.37 (sept, J = 6.8 Hz, 1H), 2.00 (d, J = 14.6 Hz, 1H), 1.97-1.88 (m, 3H), 1.74 (m, 1H), 1.65-1.54 (m, 1H), 1.45 (dd, J = 14.5, 9.6 Hz, 1H), 1.15 (s, 3H), 1.12 (d, J = 6.8 Hz, 3H), 1.10 (d, J = 6.9 Hz, 3H), 0.79 (d, J = 7.1 Hz, 3H), 0.75-0.66 (m, 1H); 13C NMR (500 MHz, $CDCl_3$) δ 155.0, 148.7, 145.4, 141.9, 129.4, 107.8, 75.2, 70.0, 57.1, 48.4, 48.0, 40.1, 38.1, 33.4, 32.5, 27.1, 24.1, 23.4, 21.7, 15.9; HRMS for $C_{20}H_{30}O_2$ (ESI, [M+H +-H2O]) calculated 285.2213, found 285.2220 (deviation 2.48 ppm). See Supplementary Table 10, Supplementary Figs. 4, 45-50 for more spectroscopic data.

Isolation of compound **11**

A. nidulans expressing SdnA-C-B-H-F was used to inoculate 3L of CDST liquid culture which was then shaken at 28 °C for 6 days. Cell body and media were separated by filtration and extracted separately by acetone and ethyl acetate respectively. The organics were combined and dried with a rotary evaporator. The crude extract was then separated by normal-phase flash chromatography with a gradient of hexane and ethyl acetate and then by reverse-phase HPLC as stated in main methods. Compound **11** was obtained as 3 mg light yellow solid (1 mg/L). 1H NMR (500 MHz, d5-Pyridine) δ 10.07 (d, J = 1.0 Hz, 1H), 5.97 (d, J = 3.4, 1H), 2.63 (sept, J = 6.8, 1H), 2.23 (m, 2H), 2.05 (t, J = 13.4 Hz, 1H), 1.92-1.78 (m, 4H), 1.53 (m, 1H), 1.39-1.28 (m, 2H), 1.24 (s, 3H), 0.98 (d, J = 6.8 Hz, 3H), 0.95 (d, J = 6.9 Hz, 3H), 0.92-0.81 (m, 2H), 0.68 (d, J = 6.6 Hz, 3H); 13C NMR (500 MHz, d5-Pyridine) δ 204.8, 175.5, 148.8, 131.5, 72.8, 62.1, 58.8, 50.9, 42.1, 41.6, 32.9, 32.3, 31.4, 29.6, 28.1, 26.8, 22.5, 22.4, 21.2, 17.6; HRMS for $C_{20}H_{28}O_3$ (ESI, [M+H$^+$-H_2O]) calculated 299.2006, found 299.2009 (deviation 1.15 ppm). See Supplementary Table 13, Supplementary Figs. 4, 62-66 for more spectroscopic data.

Isolation of compound **12**

Compound **12** was coisolated with compound **11** as 3 mg light yellow solid (1 mg/L). 1H NMR (500 MHz, d5-Pyridine) δ 5.97 (d, J = 3.6 1H), 3.74 (d, J = 11.6 Hz, 1H), 3.61 (d, J = 11.6 Hz, 1H), 2.89 (sept, J = 6.8 Hz, 1H), 2.29-2.12 (m, 4H), 2.06-1.95 (m, 3H), 1.79 (m, 1H), 1.52 (d, J = 14.0, 6.6 Hz, 1H), 1.43 (s, 3H), 1.27-1.17 (m, 2H), 1.16 (d, J = 7.0 Hz, 3H), 1.14 (d, J = 7.0 Hz, 3H), 0.83 (d, J = 6.8 Hz, 3H); 0.30 (d, J = 12.3 Hz, 1H); 13C NMR (500 MHz, d5-Pyridine) δ 179.5, 149.8, 130.6, 68.0, 63.4, 50.7, 49.9, 43.3, 42.8, 33.6, 33.0, 32.7, 32.7, 28.8, 27.1, 24.2, 24.1, 23.1, 21.6, 18.3; HRMS for $C_{20}H_{30}O_3$ (ESI, $[M+H^+-H_2O]$) calculated 301.2162, found 301.2167 (deviation 1.64 ppm). See Supplementary Table 14, Supplementary Figs. 4, 67-71 for more spectroscopic data.

Isolation of compound **13**

Compound **13** was coisolated with compound **1** as 5.5 mg light yellow solid (2.3 mg/L). 1H NMR (500 MHz, d5-Pyridine) δ 6.73 (m, 1H), 6.55-6.50 (m, 1H), 4.40-4.32 (m, 1H), 3.15 (m, 1H), 2.63 (d, J = 13.7 Hz, 1H), 2.56-2.48 (m, 1H), 2.02-1.94 (m, 2H), 1.83-1.75 (m, 1H), 1.64-1.53 (m, 2H), 1.58 (s, 3H), 1.53-1.42 (m, 1H), 1.27 (m, 1H), 1.29 (dd, J = 7.1, 3.2 Hz, 3H), 1.27 (d, J = 7.0 Hz, 3H), 1.22 (d, J = 7.0 Hz, 3H), 0.84 (d, J = 7.1 Hz, 3H); 13C NMR (500 MHz, d5-Pyridine) δ 179.6, 167.4, 164.2, 153.9, 128.4, 58.8, 44.8, 43.5, 39.9, 36.7, 35.2, 34.0, 27.9, 23.2, 22.8, 22.1, 15.1, 10.8; HRMS for $C_{20}H_{30}O_4$ (ESI, $[M+H^+-H_2O]$) calculated 317.2111, found 317.2117 (derivation 1.82 ppm). See Supplementary Table 15, Supplementary Figs. 4, 72-76 for more spectroscopic data.

Isolation of compound **14**

Compound **14** was coisolated with compound **1** as 3 mg light yellow solid (1.5 mg/L). 1H NMR (500 MHz, d5-Pyridine) δ 6.72 (m, 1H), 6.52 (m, 1H), 4.35 (m, 1H), 3.75-3.64 (m, 2H), 2.59 (d, J = 13.9, 1H), 2.41-2.28 (m, 1H), 2.07 (m, 1H), 2.01-1.88 (m, 2H), 1.58 (s, 3H), 1.54 (m, 2H), 1.47 (m, 1H), 1.41-1.30 (m, 2H), 1.27 (d, J = 7.0 Hz, 3H), 1.21 (d, J = 7.0 Hz, 3H), 0.96 (d, J = 6.7 Hz, 3H), 0.84 (d, J = 7.0 Hz, 3H); 13C NMR (500 MHz, d5-Pyridine) δ 167.4, 164.1, 154.1, 136.4, 136.4, 128.3, 68.2, 58.9, 43.4, 43.4, 36.9, 35.5, 34.3, 27.9, 23.4, 22.8, 22.2, 22.0, 15.2, 11.3; HRMS for $C_{20}H_{32}O_3$ (ESI, $[M+H^+-H_2O]$) calculated 303.2319, found 303.2322 (deviation 2.12 ppm). See Supplementary Table 16, Supplementary Figs. 4, 76-81 for more spectroscopic data.

Chemical synthesis of compounds 4, 7, and **10**

General synthetic methods were outlined in the main methods. The source, yield and analytical data of each compound were listed here.

Synthesis of compound **4**

Compound **4** was synthesized from 5 mg of compound **3** with a yield of 100% estimated from UV of the remaining reactant. 1H NMR (500 MHz, CD_2Cl_2) δ 9.95 (s, 1H), 9.48 (s, 1H), 6.20 (s, 1H), 5.97 (d, J = 0.8 Hz, 1H), 3.44-3.35 (sept, J = 7.0 Hz, 1H), 2.57 (m, 1H), 2.40 (m, 2H), 2.20 (m, 1H), 1.96-1.84 (m, 2H), 1.80 (m, 1H), 1.76-1.67 (m, 2H), 1.53-1.42 (m, 1H), 1.39 (m, 1H), 1.40-1.32 (m, 1H), 1.29 (dd, J = 14.3, 2.0 Hz, 1H), 1.16 (s, 3H), 1.10 (d, J = 6.9 Hz, 3H), 1.09 (d, J = 6.9 Hz, 3H), 0.89 (d, J = 7.1 Hz, 3H); 13C NMR (126 MHz, CD_2Cl_2) δ 195.4, 188.6, 172.5, 154.3, 142.3, 134.7, 50.3, 45.5, 43.2, 36.9, 36.5, 36.1, 33.8, 30.4, 29.6, 27.2, 26.4, 21.8, 21.8, 16.0; HRMS for $C_{20}H_{30}O_2$ (ESI, $[M+H^+]$) calculated 303.2319, found 303.2325 (deviation 2.12 ppm). See Supplementary Table 6, Supplementary Figs. 4, 25-29 for more spectroscopic data.

Synthesis of compound **7**

Compound **7** was synthesized from 3 mg of compound **5** with a yield of 100% estimated from UV of the remaining reactant. 1H NMR (500 MHz, CD_2Cl_2) δ 9.90 (s, 1H), 9.45 (s, 1H), 6.77 (d, J = 5.3 Hz, 1H), 6.44 (d, J = 5.3 Hz, 1H), 6.10 (s, 1H), 5.93 (s, 1H), 3.47 (sept, J = 6.8 Hz, 1H), 2.50 (m, 1H), 1.93-1.79 (m, 3H), 1.77-1.66 (m, 2H), 1.25 (m, 3H), 1.23 (d, J = 6.9 Hz, 3H), 1.19 (d, J = 6.9 Hz, 3H), 1.19 (s, 3H), 0.78 (d, J = 7.1 Hz, 3H); 13C NMR (500 MHz, CD_2Cl_2) δ 195.6, 184.2, 170.9, 157.0, 153.9, 143.3, 134.4, 128.5, 57.6, 45.4, 42.1, 36.3, 34.3, 33.2, 29.8, 26.5, 22.9, 22.5, 22.4, 15.0; HRMS for $C_{20}H_{28}O_2$ (ESI, [$M+Na^+$]) calculated 301.2162, found 301.2168 (deviation 1.97 ppm). See Supplementary Table 9, Supplementary Figs. 4, 40-44 for more spectroscopic data.

Synthesis of compound **10**

Compound **10** was synthesized from 5 mg of compound **6** with a yield of 95% estimated from UV of the remaining reactant. 1H NMR (500 MHz, d5-Pyridine) δ 9.61 (s, 1H), 6.67 (d, J = 5.4 Hz, 1H), 6.51 (d, J = 5.3 Hz, 1H), 6.33 (s, 1H), 5.93 (s, 1H), 4.31 (sept, J = 6.8 Hz, 1H), 2.79 (m, 1H), 2.33 (d, J = 13.9 Hz, 1H), 2.07-1.76 (m, 4H), 1.69 (m, 1H), 1.51 (s, 3H), 1.40-1.31 (m, 2H), 1.31 (d, J = 6.8 Hz, 3H), 1.19 (d, J = 6.9 Hz, 3H), 0.84 (d, J = 7.0 Hz, 3H); 13C NMR (500 MHz, d5-Pyridine) δ 195.5, 167.2, 164.2, 154.3, 153.7, 136.1, 134.5, 128.4, 58.6, 46.1, 42.2, 36.6, 34.7, 33.7, 30.3, 27.9, 23.2, 22.8, 22.2, 15.4; HRMS for $C_{20}H_{28}O_3$ (ESI, [$M+H^+-H_2O$]) calculated 299.2006, found 299.2009 (deviation 1.15 ppm). See Supplementary Table 12, Supplementary Figs.4, 57-61 for more spectroscopic data.

Preparation of compound 9

Compound **9** was a formed by cyclization of compound **7**. During the synthetic reaction of **7**, **9** was only observed as a minor product (less than 5%). However, when a solution of compound **7** **(**9 mg) in CH_2Cl_2 was evaporated in vacuum, ~ 66% of **9** cyclizes (Supplementary Fig. 51). How the concentration process promotes cyclization of **7** is not fully understood but is possibly caused by increased energy of the system due to higher collision frequency between concentrated molecules. Residue containing compounds **7** and **9** was further separated by HPLC as stated in the main methods to yield 4 mg of compound **9** as light-yellow solid. 1H NMR (500 MHz, MeOD) δ 10.15 (s, 1H), 9.81 (s, 1H), 6.13 (dd, J = 3.4, 1.4 Hz, 1H), 2.43 (dd, J = 4.3, 3.5 Hz, 1H), 2.22-2.12 (m, 1H), 2.14-2.03 (m, 3H), 2.02 (dd, J = 12.6, 4.4 Hz, 1H), 1.84 (m, 1H), 1.77-1.64 (m, 2H), 1.65 (dd, J = 14.3, 6.4 Hz, 1H), 1.32 (d, J = 12.5 Hz, 1H), 1.26-1.19 (m, 1H), 1.16 (s, 3H), 1.05-0.92 (m, 1H), 0.98 (d, J = 6.9 Hz, 3H), 0.93 (d, J = 6.9 Hz, 3H), 0.78 (d, J = 7.0 Hz, 3H); 13C NMR (500 MHz, MeOD) δ 206.9, 206.2, 165.0, 150.1, 134.1, 76.1, 63.3, 61.5, 51.9, 43.1, 42.4, 34.7, 33.2, 32.5, 30.3, 28.9, 27.1, 23.2, 21.8, 21.5, 17.8; HRMS for $C_{20}H_{28}O_2$ (ESI, [$M+H^+$]) calculated 301.2162, found 301.2172 (deviation 3.3 ppm). See Supplementary Table 11, Supplementary Figs. 4, 52-56 for more spectroscopic data.

II Supplementary Tables

Supplementary Table 1. Annotation of the putative sordaricin B cluster.

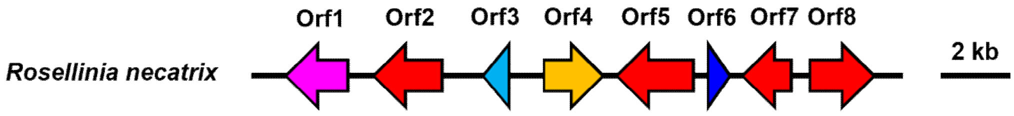

Gene	GenBank accession	Size (aa)	Proposed function	Homologs in *S. araneosa* (identity/similarity, %)	Homologs in other pathways (identity/similarity, %)
Orf1	GAP90682.1 [https://www.ncbi.nlm.nih.gov/protein/GAP90682.1]	406	Terpene cyclase	SdnA (66/79)	S0DX56.1 (38/59)
Orf2	GAW26838.1 [https://www.ncbi.nlm.nih.gov/protein/GAW26838.1]	537	P450	SdnB (62/78)	A0A084R1M7.1 (35/56)
Orf3	GAW26839.1 [https://www.ncbi.nlm.nih.gov/protein/GAW26839.1]	245	Methyltransferase	SdnD (58/75)	P74838.1 (31/48)
Orf4	GAP90684.1 [https://www.ncbi.nlm.nih.gov/protein/GAP90684.1]	514	Glycosyltransferase	SdnJ (60/79)	A0A411KZY6.1 (31/51)
Orf5	GAP90685.1 [https://www.ncbi.nlm.nih.gov/protein/GAP90685.1]	524	P450	SdnH (66/79)	Q6WP51.1 (38/58)
Orf6	GAP90686.1 [https://www.ncbi.nlm.nih.gov/protein/GAP90686.1]	144	Hypothetical protein	SdnG (51/68)	Q7V2Q8.1 (29/54)
Orf7	GAP90687.1 [https://www.ncbi.nlm.nih.gov/protein/GAP90687.1]	433	P450	SdnF (71/83)	C9K202.1 (34/53)
Orf8	GAP90688.2 [https://www.ncbi.nlm.nih.gov/protein/GAP90688.2]	520	P450	SdnE (70/82)	C8V7P3.1 (40/62)

Supplementary Table 2. DNA and protein sequence of SdnG (*S. araneosa*) used in this study.

DNA sequence	ATGGCTGGCAAGGAAATTCAGACCCCCGATCAGGCCGAGGCATTCGT<br>CGCCAAGGTATTTGACGTCCTCGATTCATATGATTACACCCGTTTCGG<br>TGAGGTGCTTTCCACCGACCTGAAGTACGAAGGTGGTTTGCAAAAGA<br>CCTCTGGGCTGGATAACTTCATCAATGACATCAAGGCATCCACCCAG<br>CGAATGCCAGGGCTCCAGACCTCTCACTCCCGATATCGCACTGAACT<br>CACCGCCGAGGGCACCATCTACAGCGAGGGTCACTCAAACGCATCTT<br>TGGAGTCGAATCCGGGCAAGGTTGTGACTGTACCCATGATTGGCGTG<br>TTCAAGTTGGACAGTGAAGATGGCAAGATCAAGGAGATGCGCATTTA<br>CAAGGATCGTTTGCCATTCTTGGCGCTCCATCAAGCACTCCCTGGGAT<br>GAAGGCGAACAATTAG
Protein sequence<br>(UniProt accession: A0A1B4XBH4, [https://www.uniprot.org/uniprot/A0A1B4XBH4])	MAGKEIQTPDQAEAFVAKVFDVLDSYDYTRFGEVLSTDLKYEGGLQKT<br>SGLDNFINDIKASTQRMPGLQTSHSRYRTELTAEGTIYSEGHSNASLESNP<br>GKVVTVPMIGVFKLDSEDGKIKEMRIYKDRLPFLALHQALPGMKANN

Supplementary Table 3. Spectroscopic data of compound **1**.

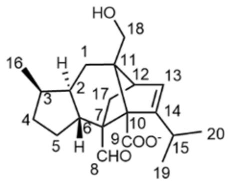

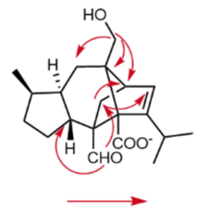

Key HMBC correlations

Position	δ_H (ppm), mult (J in Hz)	δ_C (ppm)	COSY
1	2.27, m	28.8	
2	1.77, m	41.6	H1, H3, H6
3	2.00, m	31.3	H16
4	1.80, m<br>0.92, m	32.1	H3, H4, H5
5	2.00, m<br>1.05, m	26.6	H4, H5, H6
6	2.47, m	41.7	H2, H5
7		59.1	
8	10.25	204.5	
9		175.6	
10		73.4	
11		67.2	
12	2.98, t (3.8)	46.8	H13, H17
13	6.16, d (3.3)	130.7	H12, H15
14		148.6	
15	2.74, septet (6.8)	27.9	H13, H19, H20
16	0.84, d (7.0)	17.4	H3
17	2.00, m<br>1.52, d (12.4)	29.4	H12
18	4.37, d (10.6)<br>4.24, d (10.6)	66.6	
19	1.11, d (6.7)	21.1	H15
20	1.11, d (6.8)	22.4	H15

Supplementary Table 4. Spectroscopic data of compound **2**.

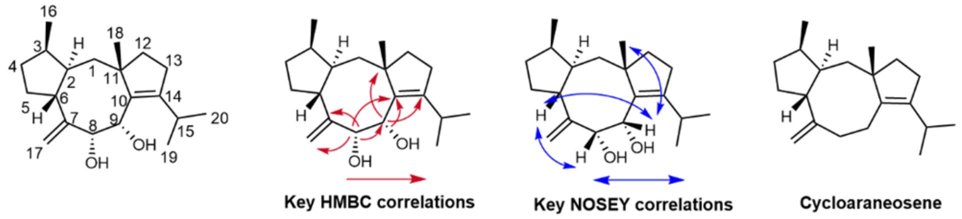

Position	δ$_H$ (ppm), mult (J in Hz)	δ$_C$ (ppm)	COSY
1	1.57, d (14.5)<br>1.32, dd (14.5, 10.4)	43.0	
2	1.65, m	50.4	H3
3	2.02, m	41.5	H2, H16
4	1.39, m<br>1.33, m	34.4	H3, H5
5	2.09, m<br>1.65, m	34.6	H4, H6
6	1.86, m	45.6	H5
7		155.9	
8	4.47 dt (5.4, 1.6)	76.3	H9, H17
9	4.34 d (5.4)	69.1	H8
10		137.8	
11		50.6	
12	1.58, m<br>1.32, m	34.4	H13
13	2.14, m	26.8	H12
14		146.8	
15	3.14, septet (7.0)	28.4	H19, H20
16	0.78, d (7.2)	14.7	H3
17	5.15, t (1.0)<br>4.93, s	106.6	H8
18	0.96, s	27.3	
19	0.91, d (6.7)	21.7	H15
20	0.94, d (6.9)	20.7	H15

The stereochemistry of **2** was determined by NOESY based on the reported stereochemistry for cycloaraneosene[3,4].

Supplementary Table 5. Spectroscopic data of compound **3**

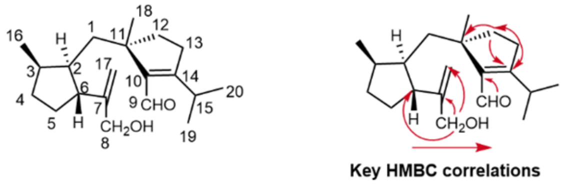

Position	δH (ppm), mult (J in Hz)	δC (ppm)	COSY
1	1.69, dd (14.2, 9.5)<br>1.50, m	36.1	
2	1.60, m	44.9	H3
3	2.17, m	36.1	H2, H16
4	1.78, m<br>1.37, m	33.5	H3
5	1.85, m<br>1.37, m	28.9	H6
6	2.17, m	48.2	H5
7		152.2	
8	3.99, m	64.6	H17
9	9.97	188.9	
10		142.0	
11		50.1	
12	1.80, m<br>1.34, m	35.6	H13
13	2.43, m	30.1	H12
14		172.8	
15	3.41, septet (7.0)	26.9	H19, H20
16	0.86, d (7.1)	16.1	H3
17	5.09, q (1.6)<br>4.85, s	109.4	H8
18	1.19, s	26.4	
19	1.11, d (6.9)	21.7	H15
20	1.09, d (6.9)	21.5	H15

Supplementary Table 6. Spectroscopic data of compound **4**.

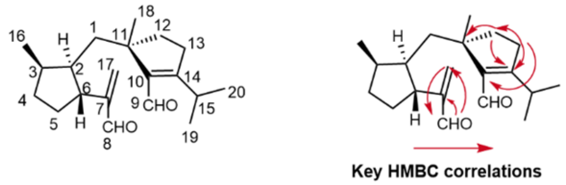

Position	δ_H (ppm), mult (J in Hz)	δ_C (ppm)	COSY
1	1.71, m<br>1.31, dd (14.2, 1.9)	36.5	
2	1.79, m	45.5	H3, H6
3	2.21, m	37.0	H2, H16
4	1.91, m<br>1.37, m	33.8	H3
5	1.91, m<br>1.37, m	29.6	H6
6	2.58, m	43.2	H2, H5
7		154.3	
8	9.48, s	195.4	
9	9.95, s	188.6	
10		142.3	
11		50.3	
12	1.71, m<br>1.50, m	36.1	H13
13	2.43, m	30.4	H12
14		172.5	
15	3.40, septet (6.9)	27.2	H19, H20
16	0.86, d (7.1)	16.0	H3
17	6.20<br>5.97, d (0.6)	134.7	
18	1.16, s	26.4	
19	1.10, d (6.9)	21.8	H15
20	1.09, d (6.9)	21.8	H15

Supplementary Table 7. Spectroscopic data of compound **5**

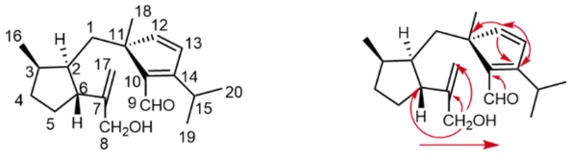

Key HMBC correlations

Position	δ_H (ppm), mult (J in Hz)	δ_C (ppm)	COSY
1	2.27, dd (14.0, 3.0)<br>1.90, m	34.9	H2
2	1.49, m	45.0	H1
3	1.90, m	36.4	H16
4	1.67, m<br>1.24, m	33.3	H3, H4, H5
5	1.90, m<br>1.49, m	30.1	H4, H5, H6
6	2.35, m	47.9	H5
7		153.8	
8	4.36, m	64.5	H17
9	10.2	184.4	
10		144.0	
11		57.8	
12	6.86, d (5.0)	157.2	H13
13	6.49, d (5.2)	128.7	H12
14		170.0	
15	3.45, septet (7.0)	26.4	H19, H20
16	0.82, d (7.0)	15.5	H3
17	5.55, t (1.9)<br>5.03, s	108.4	H8, H17
18	1.37, s	22.8	
19	1.14, d (6.8)	22.7	H15
20	1.11, d (6.8)	22.5	H15

Supplementary Table 8. Spectroscopic data of compound **6**.

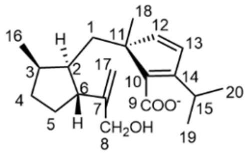

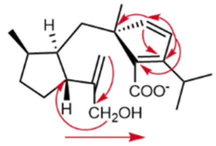

Key HMBC correlations

Position	δ_H (ppm), mult (J in Hz)	δ_C (ppm)	COSY
1	2.52, d (13.4)<br>1.94, m	34.0	H1, H2
2	1.49, m	45.0	H1
3	2.07, m	36.2	H4, H16
4	1.73, m<br>1.26, m	33.3	H3, H4, H5
5	1.94, m<br>1.51, m	29.8	H4, H5, H6
6	2.44, m	48.1	H2, H5
7		153.9	
8	4.46, d (14.9)<br>4.40, d (14.9)	64.4	H8, H17
9		167.6	
10		136.6	
11		58.7	
12	6.73, d (5.3)	153.9	H13
13	6.52, d (5.2)	128.2	H12
14		163.7	
15	4.33, m	27.8	H19, H20
16	0.88, d (7.2)	15.4	H3
17	5.54, s<br>5.16, s	108.3	H8, H17
18	1.57, s	23.3	
19	1.26, d (7.0)	22.7	H15
20	1.20, d (7.0)	22.3	H15

Supplementary Table 9. Spectroscopic data of compound **7**.

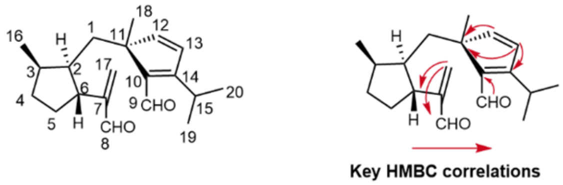

Position	δ_H (ppm), mult (J in Hz)	δ_C (ppm)	COSY
1	1.90, d (14.1, 2.9)<br>1.73, dd (14.0, 10.1)	34.3	
2	1.25, m	45.4	H3
3	1.85, m	36.3	H16
4	1.73, m<br>1.25, m	33.2	H5
5	1.88, m<br>1.25, m	29.8	H4, H6
6	2.49, m	42.1	H5
7		153.9	
8	9.45 s	195.5	
9	9.90 s	184.2	
10		143.26	
11		57.6	
12	6.45, d (5.4)	145.4	H13
13	6.76, d (5.3)	157.0	H12
14		170.9	
15	3.47, septet (6.7)	26.5	H19, H20
16	0.79, d (7.2)	15.0	H3
17	6.10, s<br>5.93, s	134.4	
18	1.19, s	22.5	
19	1.23, d (6.9)	22.9	H15
20	1.19, d (6.9)	22.4	H15

Supplementary Table 10. Spectroscopic data of compound **8**.

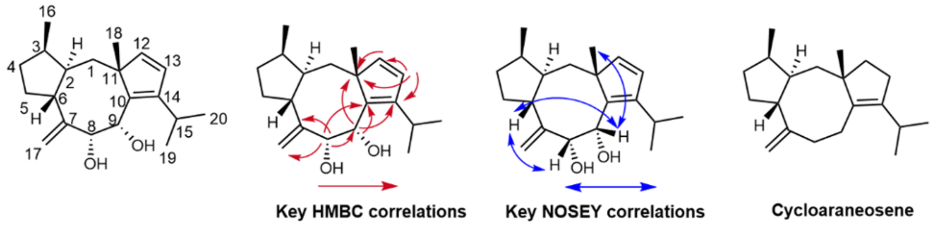

Position	δ_H (ppm), mult (J in Hz)	δ_C (ppm)	COSY
1	1.98, d (14.4)<br>1.43, dd (14.4, 9.4)	38.1	
2	0.70, q	48.0	H3
3	1.94, m	40.1	H16
4	1.72, m<br>1.28, m	33.4	H3, H5
5	1.94, m<br>1.60, m	32.5	H4, H6
6	1.94, m	48.4	H5
7		155.1	
8	4.56 d (5.6)	75.2	H9, H17
9	4.67 d (5.6)	70.0	H8
10		141.9	
11		57.1	
12	6.01, d (5.4)	145.4	H13
13	6.25, d (5.5)	129.4	H12
14		148.7	
15	3.37, septet (6.8)	27.1	H19, H20
16	0.78, d (7.0)	15.9	H3
17	5.20, t (1.2)<br>4.90, s	107.8	H8
18	1.15, s	24.1	
19	1.02, d (6.8)	23.7	H15
20	1.00, d (6.7)	21.4	H15

The stereochemistry of **8** was determined by NOESY based on the reported stereochemistry for cycloaraneosene[3,4]

Supplementary Table 11. Spectroscopic data of compound **9**.

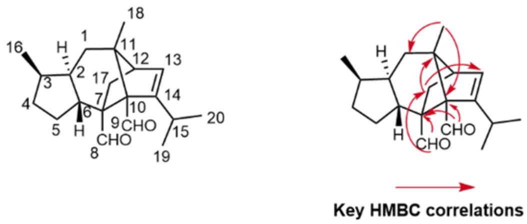

Position	δ_H (ppm), mult (J in Hz)	δ_C (ppm)	COSY
1	2.10, t (13.5)<br>1.65, dd (14.0, 6.5)	32.9	H2
2	1.84, m	43.1	H1
3	2.07, m	32.5	H16
4	2.07, m<br>1.21, m	33.2	H5
5	1.70, m<br>0.97, m	27.1	H4, H6
6	2.17, m	42.4	H5
7		61.5	
8	9.80, s	206.1	
9	10.15, s	206.9	
10		76.1	
11		63.3	
12	2.43, dd (4.3, 3.5)	51.9	H13, H17
13	6.13, dd (3.3, 1.3)	134.1	H12, H15
14		150.1	
15	2.17, m	28.9	H13, H19, H20
16	0.78, d (7.2)	17.8	H3
17	2.00, dd (12.5, 4.3)<br>1.31, d (12.6)	30.3	H12
18	1.16, s	21.5	
19	0.93, d (6.8)	23.2	H15
20	0.98, d (6.7)	21.8	H15

Supplementary Table 12. Spectroscopic data of compound **10**.

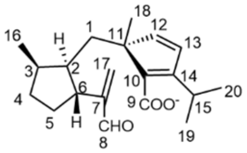

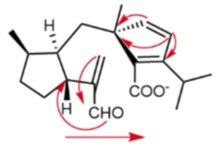

Key HMBC correlations

Position	δH (ppm), mult (J in Hz)	δC (ppm)	COSY
1	2.33, d (13.9)<br>1.89, dd (14, 10.2)	34.7	H2
2	1.70, m	46.1	H3, H6
3	2.03, m	36.6	H2, H16
4	1.81, m<br>1.31, m	33.6	H3
5	1.96, m<br>1.36, m	30.3	H6
6	2.78, m	42.2	H2, H5, H17
7		154.3	
8	9.61, s	195.5	H17
9		167.2	
10		136.1	
11		58.6	
12	6.67, d (5.4)	153.7	H13
13	6.50, d (5.4)	128.4	H12
14		164.2	
15	4.31, septet (6.8)	27.9	H19, H20
16	0.83, d (7.0)	15.3	H3
17	6.33, s<br>5.93, s	134.5	H6, H8
18	1.51, s	23.3	
19	1.31, d (6.8)	22.8	H15
20	1.19, d (6.8)	22.2	H15

Supplementary Table 13. Spectroscopic data of compound **11**.

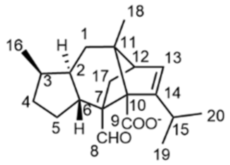

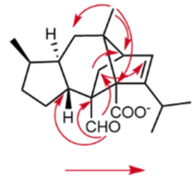

Key HMBC correlations

Position	δ_H (ppm), mult (J in Hz)	δ_C (ppm)	COSY
1	2.05, t (13.4)<br>1.35, dd (14.3, 6.4)	32.9	
2	1.53, m	42.1	
3	1.87, m	31.4	H16
4	1.80, m<br>0.92, m	32.3	H3, H5
5	1.87, m<br>0.87, m	26.8	H4, H6
6	2.26, m	41.6	H5
7		58.8	
8	10.07, s	204.8	
9		175.4	
10		72.8	
11		62.1	
12	2.23, m	50.9	H13
13	5.97, d (3.4)	131.4	H12, H15
14		148.7	
15	2.63, septet (6.7)	28.1	H13, H19, H20
16	0.68, d (6.7)	17.6	H3
17	1.80, t (13.4)<br>1.30, d (12.6)	29.6	H12
18	1.24, s	22.5	
19	0.95, d (6.7)	22.4	H15
20	0.98, d (6.8)	21.1	H15

Supplementary Table 14. Spectroscopic data of compound **12**.

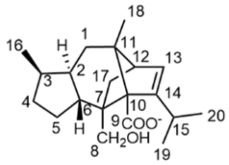

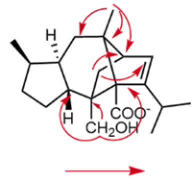

Key HMBC correlations

Position	δ_H (ppm), mult (J in Hz)	δ_C (ppm)	COSY
1	2.19, m<br>1.51, dd (14.3, 6.4)	33.6	H2
2	1.79, m	42.8	H1
3	2.01, m	32.7	H16
4	2.19, m<br>1.20, m	27.1	H5
5	2.01, m<br>1.11, m	32.7	H4, H6
6	2.23, m	43.3	H5
7		49.9	
8	3.75, d (11.6)<br>3.62, d (11.9)	68.0	
9		179.5	
10		72.6	
11		63.4	
12	2.23, m	50.7	H13
13	5.97, d (3.4)	130.6	H12, H15
14		149.8	
15	2.89, septet (6.8)	28.8	H13, H19, H20
16	0.84, d (6.9)	18.3	H3
17	2.01, m<br>0.31, d (12.3)	33.0	
18	1.43, s	24.1	
19	1.16, d (7.0)	23.1	H15
20	1.14, d (7.0)	21.5	H15

Supplementary Table 15. Spectroscopic data of compound **13**.

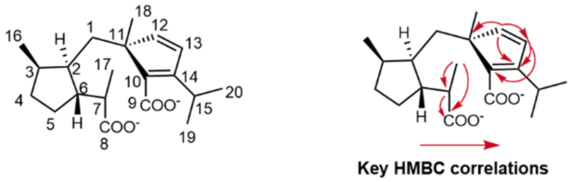

Position	δ_H (ppm), mult (J in Hz)	δ_C (ppm)	COSY
1	2.64, d (13.4)<br>1.97, m	35.2	H2
2	1.45, m	43.5	H1
3	1.98, m	36.7	H4, H16
4	1.57, m<br>1.27, m	34.0	H3, H5
5	1.79, m<br>1.65, m	23.2	H4, H6
6	2.52, m	44.8	H2, H5
7	3.15, m	39.9	H6, H17
8		179.6	
9		167.4	
10		135.9	
11		58.8	
12	6.73, m	153.9	H13
13	6.52, m	128.3	H12
14		164.2	
15	4.36, m	27.9	H19, H20
16	0.85, d (7.1)	15.1	H3
17	1.29, dd (7.1, 3.2)	10.8	H7
18	1.57, s	23.2	
19	1.27, d (7.0)	22.8	H15
20	1.22, d (7.0)	22.1	H15

Supplementary Table 16. Spectroscopic data of compound **14**.

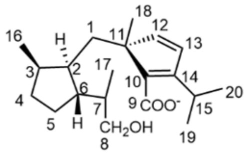

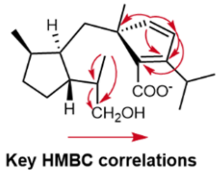

Position	δ_H (ppm), mult (J in Hz)	δ_C (ppm)	COSY
1	2.60, d (13.8)<br>1.92, m	35.5	H2
2	1.47, m	43.4	H1
3	1.92, m	36.9	H4, H16
4	1.54, m<br>1.35, m	34.3	H3, H5
5	1.54, m<br>1.35, m	22.0	H4
6	2.08, m	43.4	H7
7	2.33, m	36.9	H6, H8, H17
8	3.69, m	68.2	H7
9		167.4	
10		136.4	
11		58.9	
12	6.73, m	154.1	H13
13	6.52, m	128.3	H12
14		164.1	
15	4.35, m	27.9	H19, H20
16	0.84, d (7.1)	15.2	H3
17	0.96, d (6.8)	11.3	H7
18	1.58, s	23.4	
19	1.27, d (7.0)	22.8	H15
20	1.21, d (7.0)	22.2	H15

III Supplementary Figures

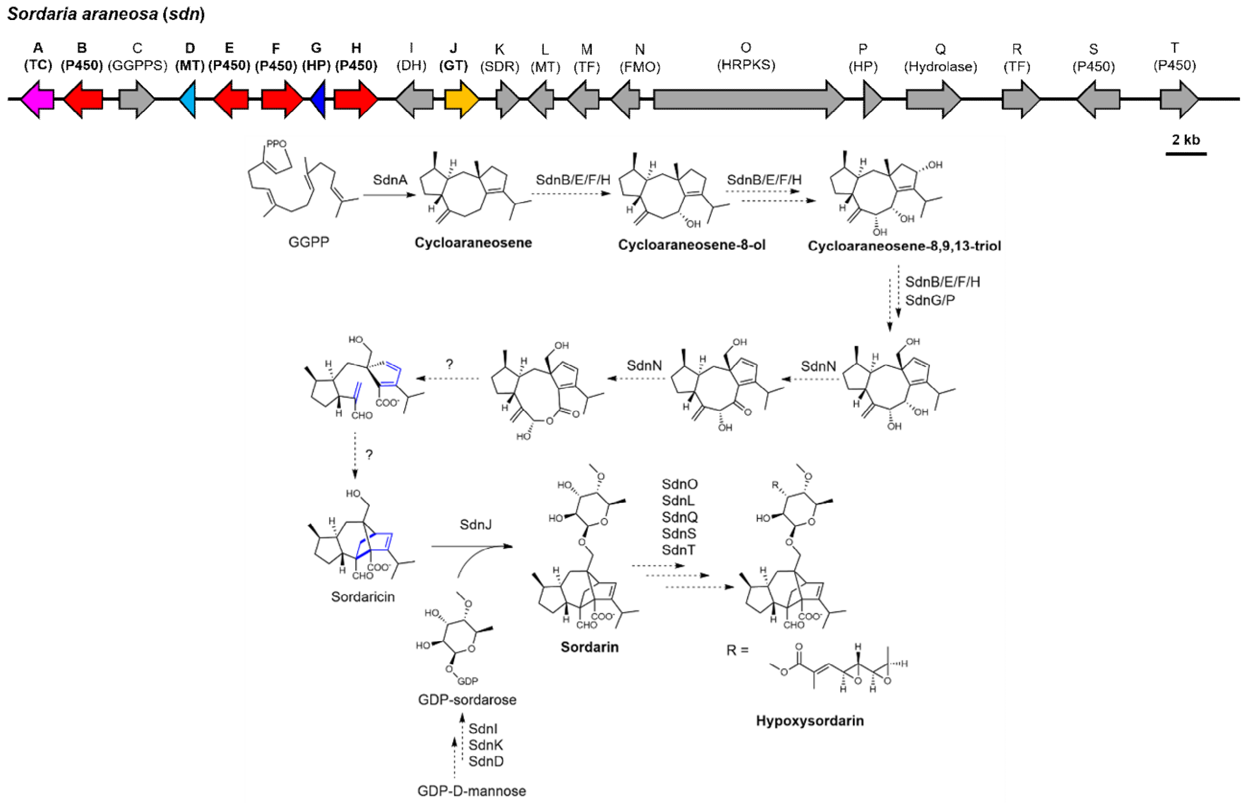

Supplementary Fig 1. Proposed biosynthesis of sordaricin, sordarin, and hypoxysordarin by Kudo, et al[19]. Genes in *sdn* but absent from the sordarin B cluster are colored gray. Solid arrows represent experimentally characterized steps while dashed arrows represent proposed reactions. Five structures named in bold were isolated from *S. araneosa* and characterized by the same study. TC, terpene cyclase; GGPPS, geranylgeranyl pyrophosphate synthase; MT, methyl transferase; HP, hypothetical protein; DH, dehydratase; GT, glycosyl transferase; SDR, short chain reductase; TF, transcription factor; FMO, flavin-dependent monooxygenase; HRPKS, highly reducing polyketide synthase.

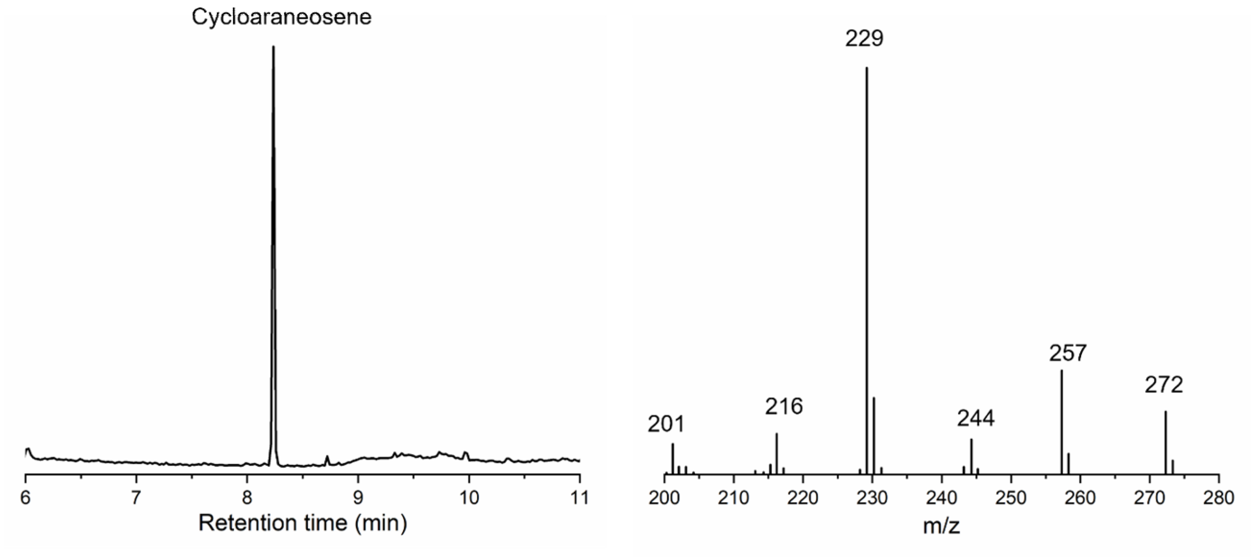

Supplementary Fig 2. GC-MS analysis of *A. nidulans* transformed with SdnA-C. The fragmentation pattern of cycloaraneosene (MW 272) matches with that reported by Kudo, et al[4].

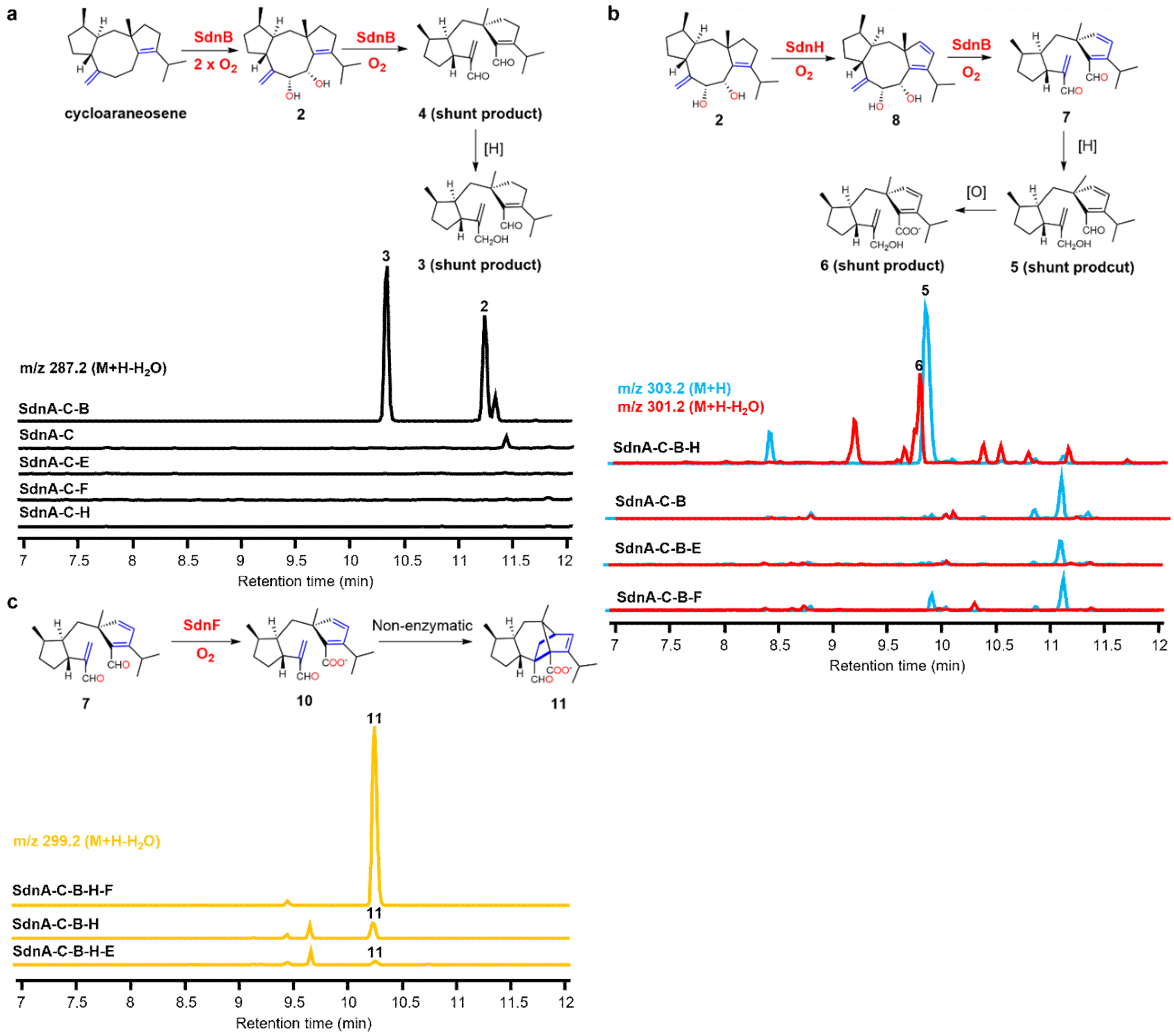

Supplementary Fig 3. Stepwise reconstitution of genes involved in sordaricin biosynthesis. **a**, metabolic profiles of *A.nidulans* coexpressing SdnA-C with SdnB/E/F/H individually. **b**, metabolic profiles of *A.nidulans* coexpressing SdnA-C-B with SdnE/F/H individually. **c**, metabolic profiles of *A.nidulans* coexpressing SdnA-C-B-H with SdnE/F individually. The trace mount of **11** in *A.nidulans* expressing SdnA-C-B-H and A-C-B-H-E is likely a result of air or host oxidation of compound **7** to **10** which then undergo non-enzymatic cyclization to form **11**. The chromatograms in all cases are extracted from mass spectra of the base peak for each compound.

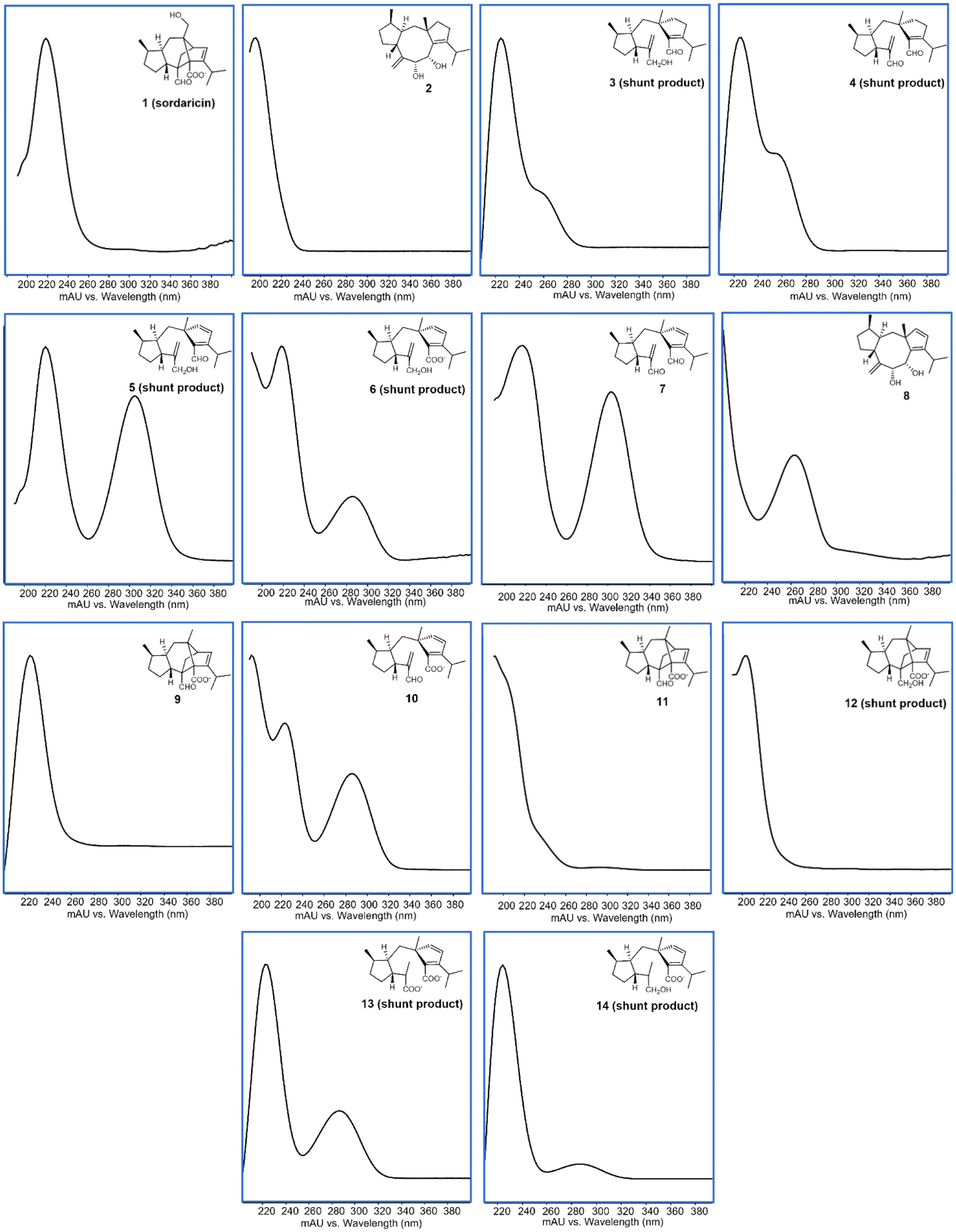

Supplementary Fig 4. UV-vis spectra of purified compounds. All spectra were measured with an Agilent 1260 Infinity II LC system equipped with a variable wavelength detector.

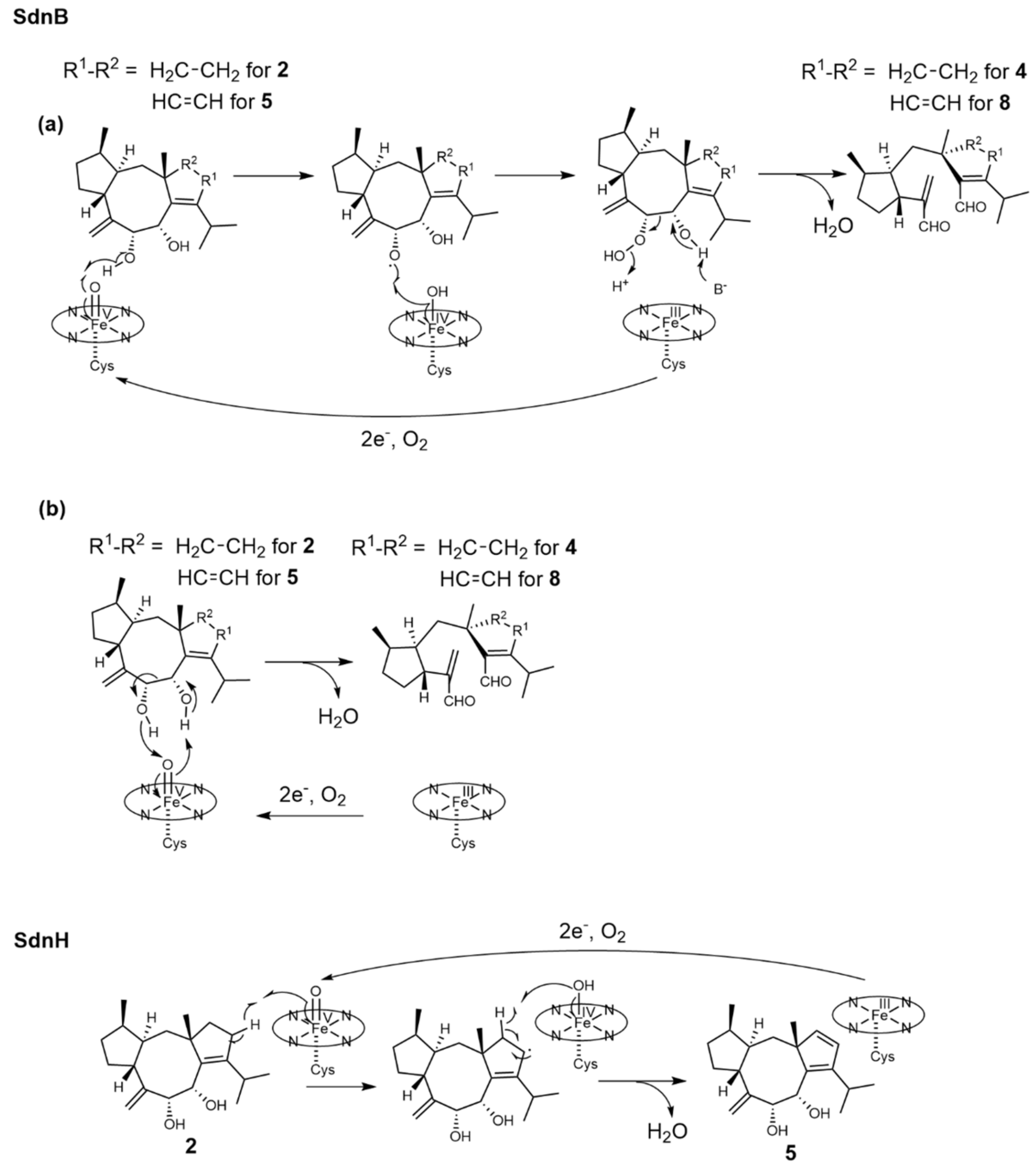

Supplementary Fig 5. Proposed mechanism of SdnB (diol cleavage[5]) and SdnH (desaturation[6]).

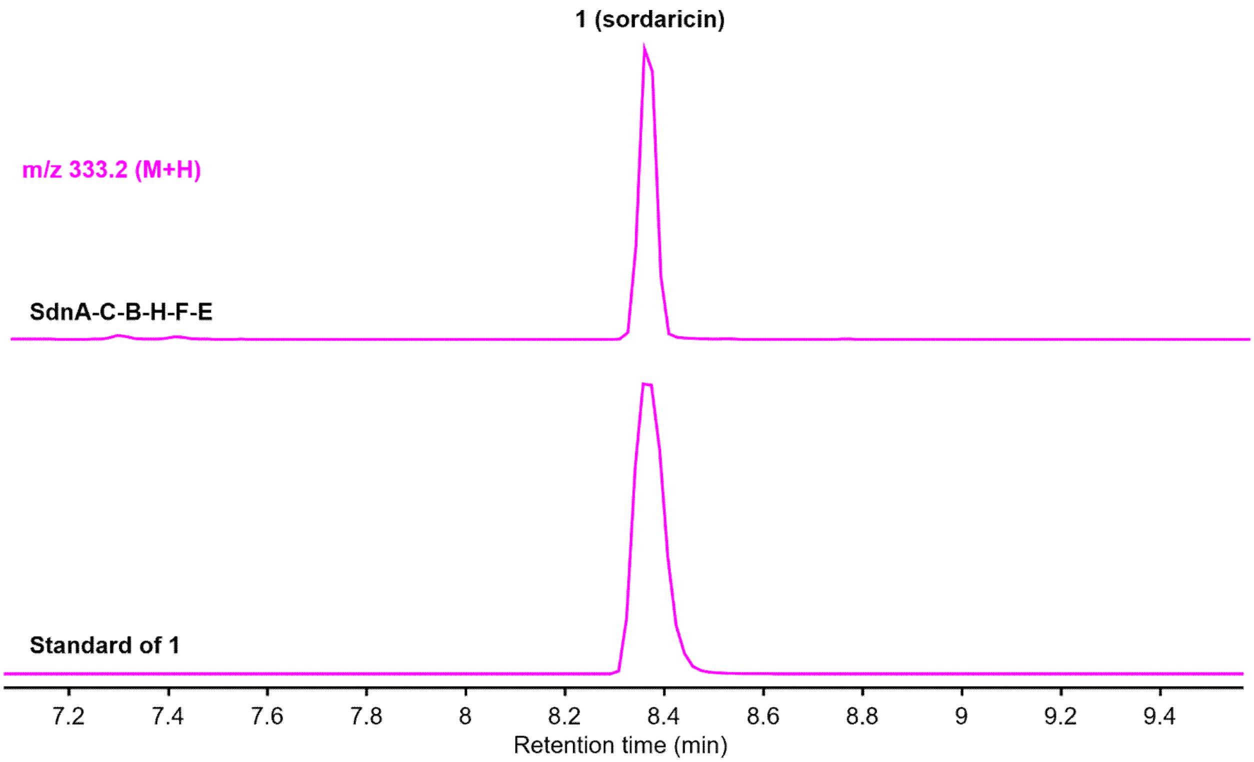

Supplementary Fig 6. Reconstitution of sordaricin biosynthesis in *S. cerevisiae* RC01.

	Description	Max Score	Total Score	Query Cover	E value	Per. Ident	Acc. Len	Accession
	Eutypa lata UCREL1 contig_2090, whole genome shotgun sequence	177	177	87%	9e-51	54.44%	69956	AORF01002090.1
	Scedosporium aurantiacum strain WM 09.24 scaffold-2, whole genome shotgun sequence	117	229	93%	8e-47	70.73%	314905	JUDQ01000002.1
	Reticulascus tulasneorum strain NRRL18230 unitig_0, whole genome shotgun sequence	110	199	100%	6e-44	69.23%	5257612	LSAX01000001.1
	Reticulascus tulasneorum strain NRRL18230 scaffold00019, whole genome shotgun sequence	110	199	100%	6e-44	69.23%	948340	LSAY01000019.1
	Trichoderma pleuroti strain TPhu1 contig118, whole genome shotgun sequence	157	157	99%	1e-43	44.12%	69223	MDJU01000118.1
	Neopestalotiopsis sp. 37M scaffold_3, whole genome shotgun sequence	102	188	98%	9e-41	67.12%	1371024	SWKT01000003.1
	Periconia macrospinosa strain DSE2036 DM02scaffold_371_Cont1150, whole genome shotgun sequence	101	186	87%	4e-40	65.75%	30277	PCYO01001150.1
	Talaromyces borbonicus strain SV-2017a Contig0000011, whole genome shotgun sequence	100	206	99%	8e-40	69.44%	718236	NBSA01000011.1
	Trichoderma afroharzianum cultivar MRI349 Contig615, whole genome shotgun sequence	136	136	99%	2e-36	43.43%	43395	JAEKOX010000615.1
	Trichoderma harzianum strain T6776 Scaffolds0269.1, whole genome shotgun sequence	136	136	99%	2e-36	43.43%	49334	JOKZ01000269.1
	Trichoderma harzianum strain T11_W Scaffold7_1, whole genome shotgun sequence	136	136	99%	2e-36	43.43%	1590712	WUWT01000010.1
	Calcarisporium arbuscula strain NRRL 3705 Contig4, whole genome shotgun sequence	118	173	86%	2e-36	55.36%	2470260	WBSA01000004.1
	Trichoderma harzianum strain Tr1 Tr1_contig_267, whole genome shotgun sequence	105	172	98%	5e-36	51.38%	210298	MTYI01000253.1
	Talaromyces verruculosus strain TS63-9 Scaffold63, whole genome shotgun sequence	117	199	100%	2e-29	41.50%	172023	LHCL01000063.1
	Xylaria longipes strain IHI A66 Contig_4, whole genome shotgun sequence	115	115	97%	3e-29	39.46%	259134	NQIL01000004.1
	Talaromyces adpressus strain CBS 142503 Contig0000575, whole genome shotgun sequence	113	198	100%	2e-28	41.50%	184356	NHZS01000561.1
	Talaromyces thailandensis strain OC-R06-P5 OC-R06-P5_contig_60, whole genome shotgun sequence	68.9	131	86%	7e-24	46.58%	288724	JACVQZ010000060.1
	Xylaria multiplex strain DSM 110363 Contig_156, whole genome shotgun sequence	69.3	129	82%	4e-23	43.04%	93426	WUBL01000156.1
	Neopestalotiopsis clavispora strain IHI 201606 Contig_4, whole genome shotgun sequence	97.8	184	98%	9e-23	63.01%	420075	JAANBA010000004.1
	Fungal sp. No.14919 DNA, contig: Contig0070, whole genome shotgun sequence	65.9	127	80%	1e-22	38.96%	217582	BDMC01000070.1
	Xylaria hypoxylon strain DSM 108379 Contig_44, whole genome shotgun sequence	95.1	95.1	84%	7e-22	39.26%	191544	SKBN01000044.1
	Xylaria hypoxylon strain CBS 122620 genome assembly, contig: tig00000792, whole genome shotgun sequence	95.1	95.1	84%	7e-22	39.26%	1026764	CADCXB010000050.1
	Xylaria grammica strain EL000614 Contig15, whole genome shotgun sequence	62.0	123	80%	2e-21	53.12%	3880253	NGZP02000014.1
	Xylaria grammica strain IHI A82 Contig_294, whole genome shotgun sequence	62.0	123	80%	2e-21	53.12%	51925	RYZI01000294.1
	Rosellinia necatrix DNA, contig: Contig87_1, strain: W97, whole genome shotgun sequence	91.7	91.7	84%	1e-20	37.72%	91525	BBSO02000556.1
	Pestalotiopsis sp. CR013 tig00037640_pilon_pilon_pilon, whole genome shotgun sequence	91.3	181	86%	2e-20	75.00%	7274155	JACFXT010000010.1
	Lecanicillium fungicola 150-1 genome assembly, contig: contig_232, whole genome shotgun sequence	82.0	131	85%	2e-17	37.88%	186091	FWCC01000232.1
	Hyphodiscus hymeniophilus strain ATCC 34498 Hyd_11, whole genome shotgun sequence	78.6	137	69%	4e-16	53.62%	1583289	VNKQ01000012.1
*	Chalara longipes BDJ N431scaffold_16_Cont117, whole genome shotgun sequence	50.8	99.0	74%	2e-06	48.94%	2141128	VKGA01000117.1

Supplementary Fig 7. Blast research results for SdnG homologs in NCBI genomic database. SdnG from *S. araneosa* was used as query. All identified homologs of SdnG cluster with other Sdn enzymes responsible for sordaricin biosynthesis except for the last entry (highlighted with *) which is an orphan gene.

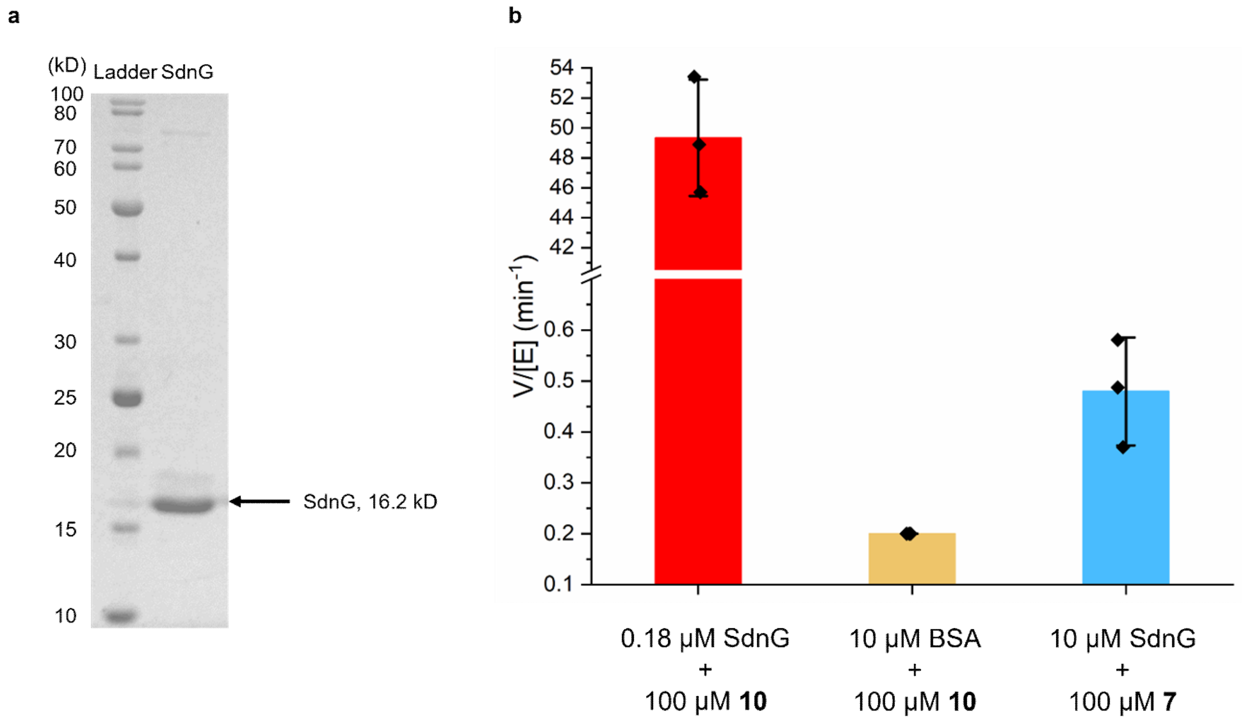

Supplementary Fig 8. Characterization of SdnG. **a**, SDS-PAGE (12%) of purified SdnG. **b**, Rates of cyclization of **10** and **7** (100 µM) in the presence of SdnG or BSA. The bars and error bars represent the mean and standard deviation of n=3 independent measurements respectively. The rate with 10 µM BSA is within the detection limit of the assay (0.2 min^{-1}). Source data are provided as a Source Data file.

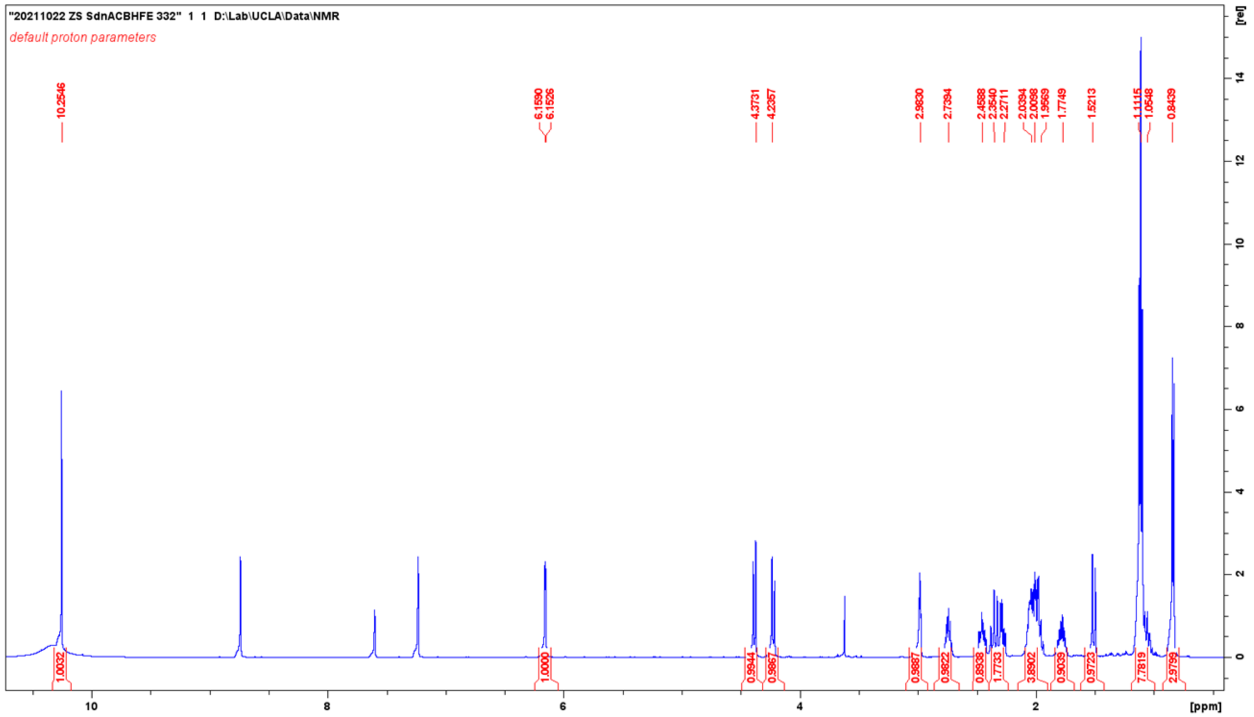

Supplementary Fig 9. ^{1}H NMR of compound **1** in d5-pyridine, 500 MHz.

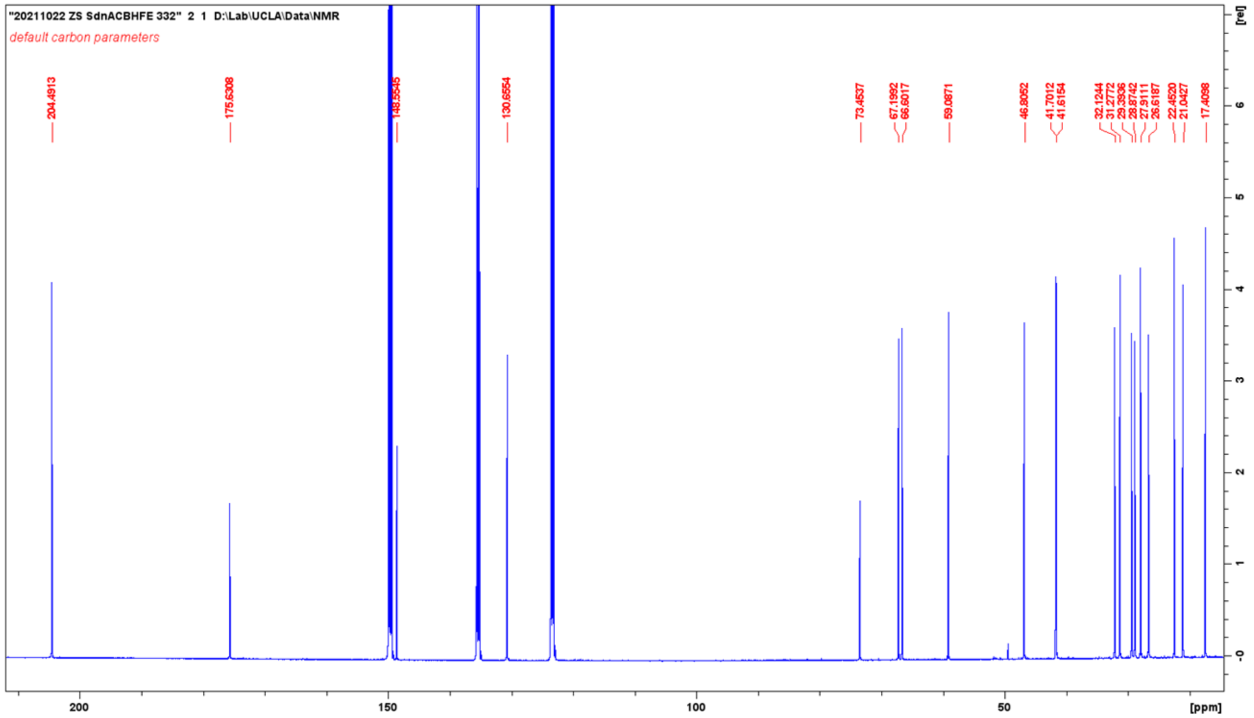

Supplementary Fig 10. ^{13}C NMR of compound **1** in d5-pyridine, 500 MHz.

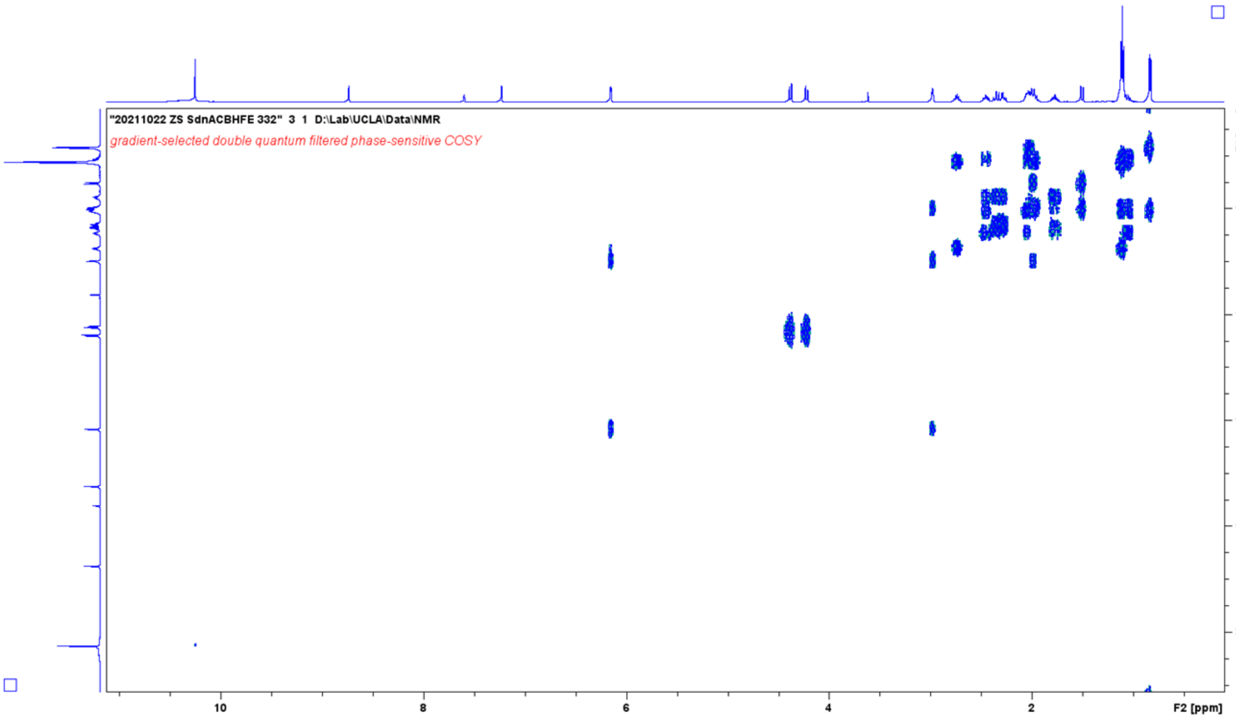

Supplementary Fig 11. ^{1}H-^{1}H COSY of compound **1** in d5-pyridine, 500 MHz.

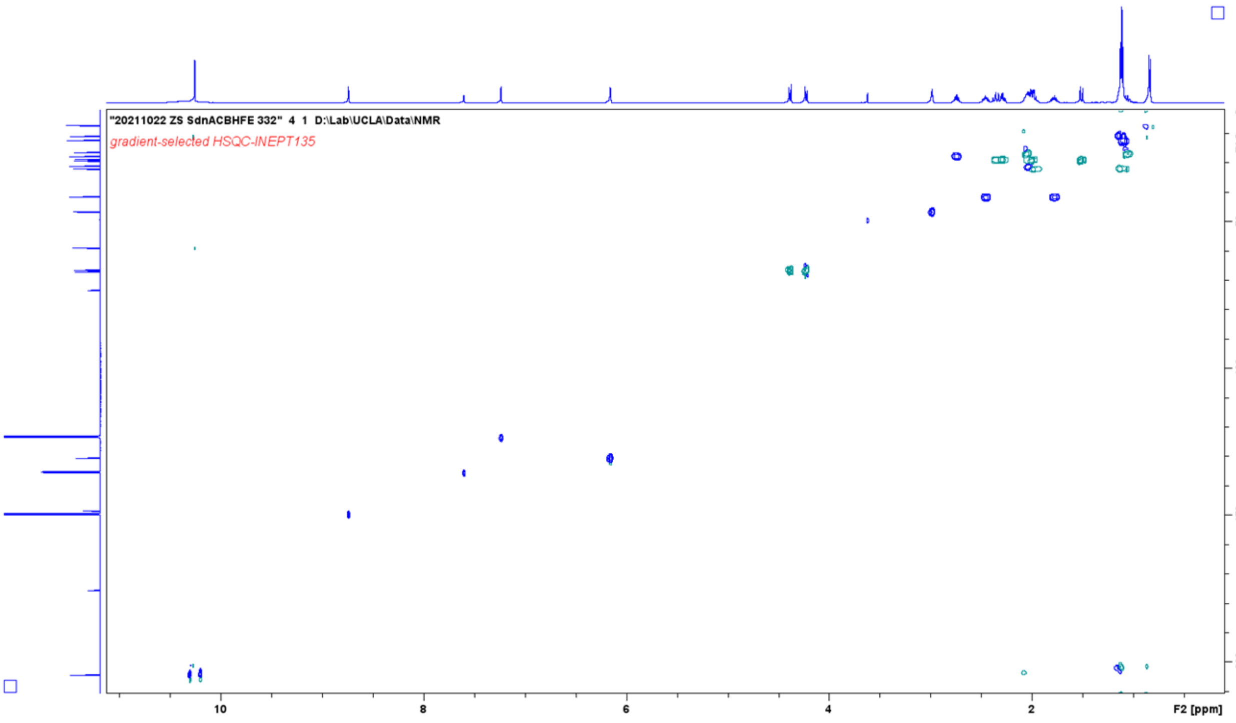

Supplementary Fig 12. ^{1}H-^{13}C HSQC of compound **1** in d5-pyridine, 500 MHz.

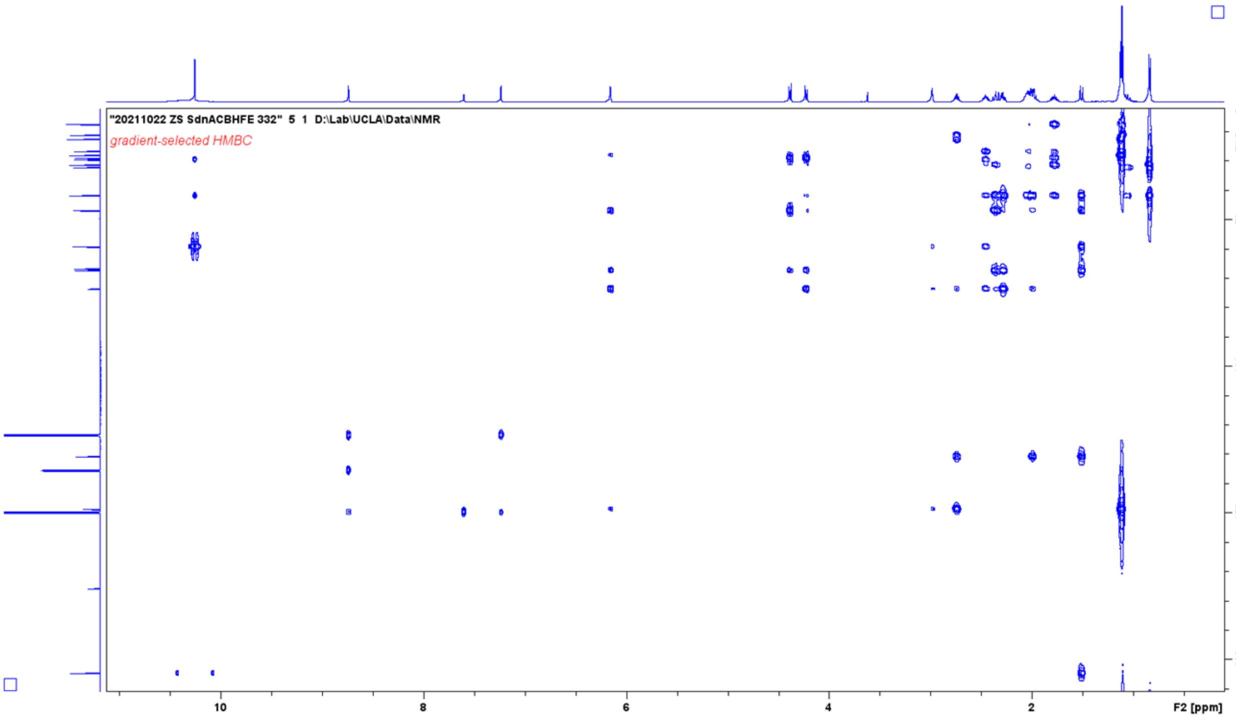

Supplementary Fig 13. ^{1}H-^{13}C HMBC of compound **1** in d5-pyridine, 500 MHz.

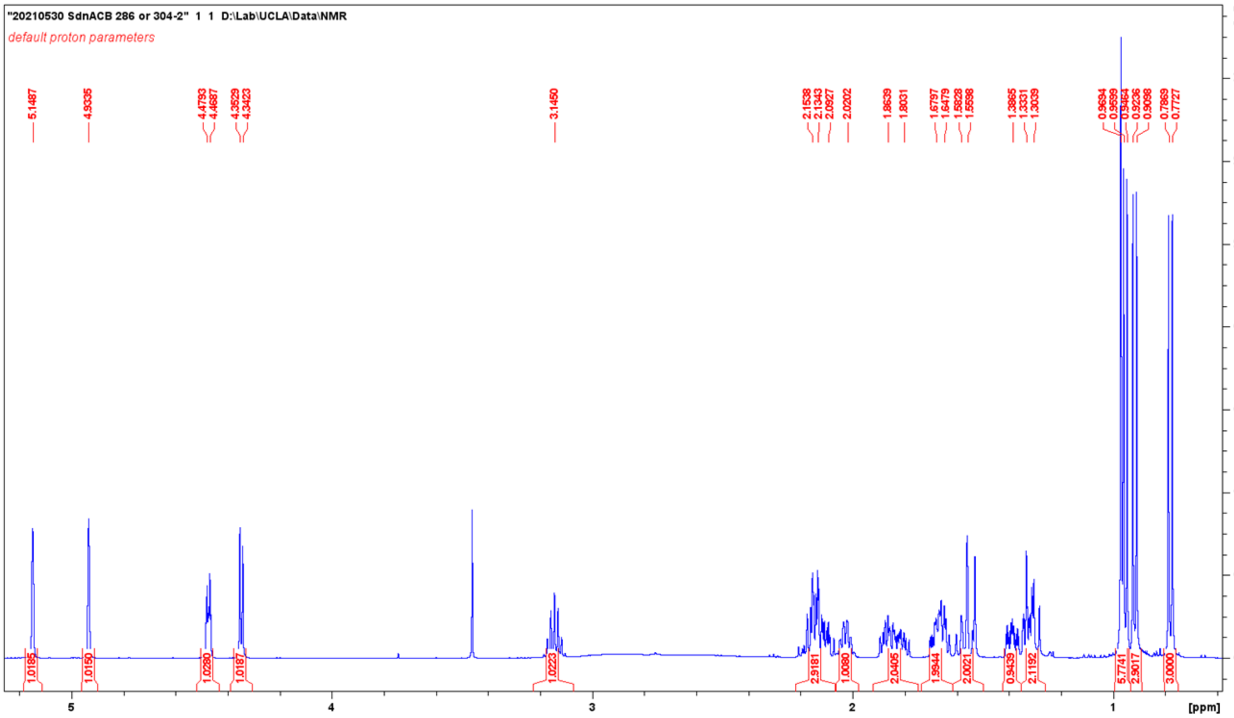

Supplementary Fig 14. ^{1}H NMR of compound **2** in $CDCl_3$, 500 MHz.

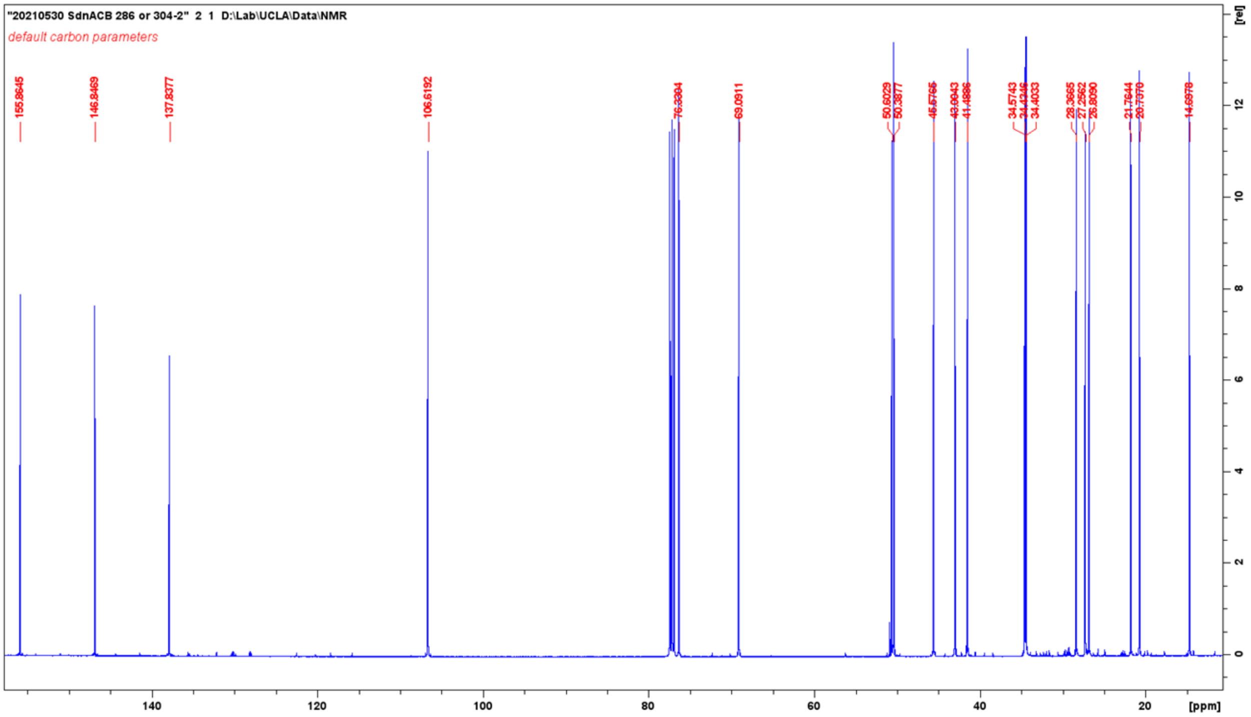

Supplementary Fig 15. ^{13}C NMR of compound **2** in $CDCl_3$, 500 MHz.

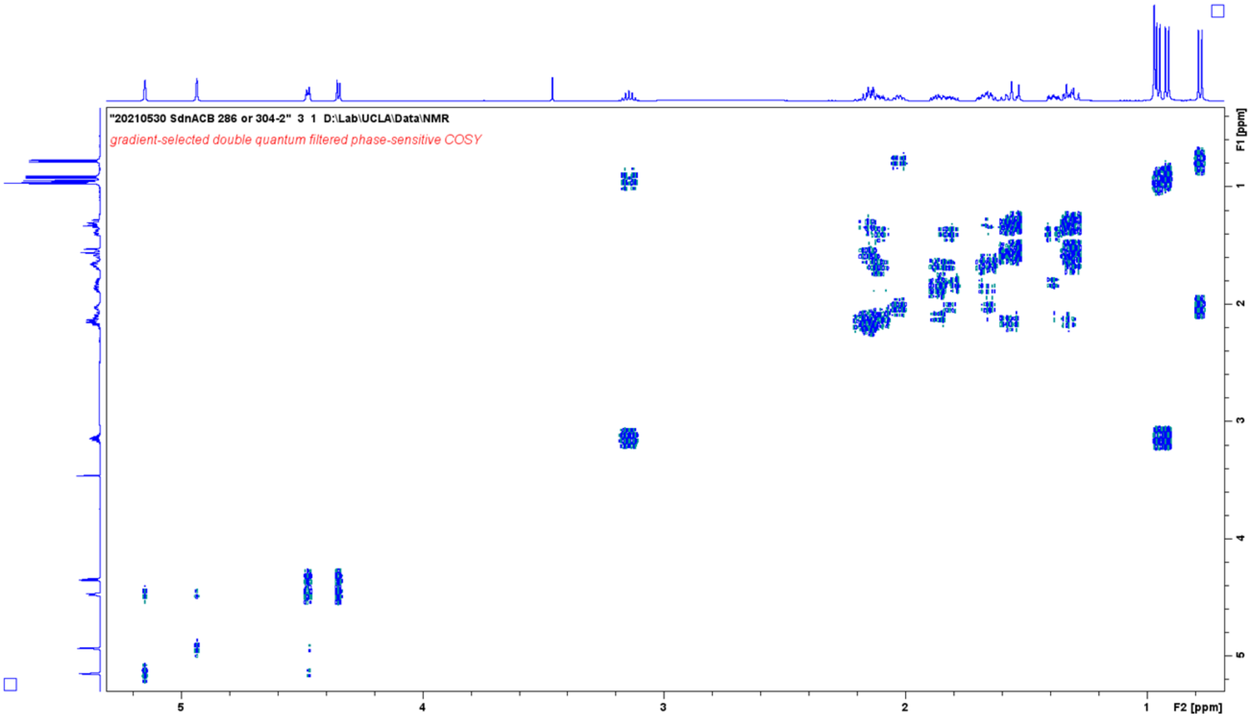

Supplementary Fig 16. ^{1}H-^{1}H COSY of compound **2** in $CDCl_3$, 500 MHz.

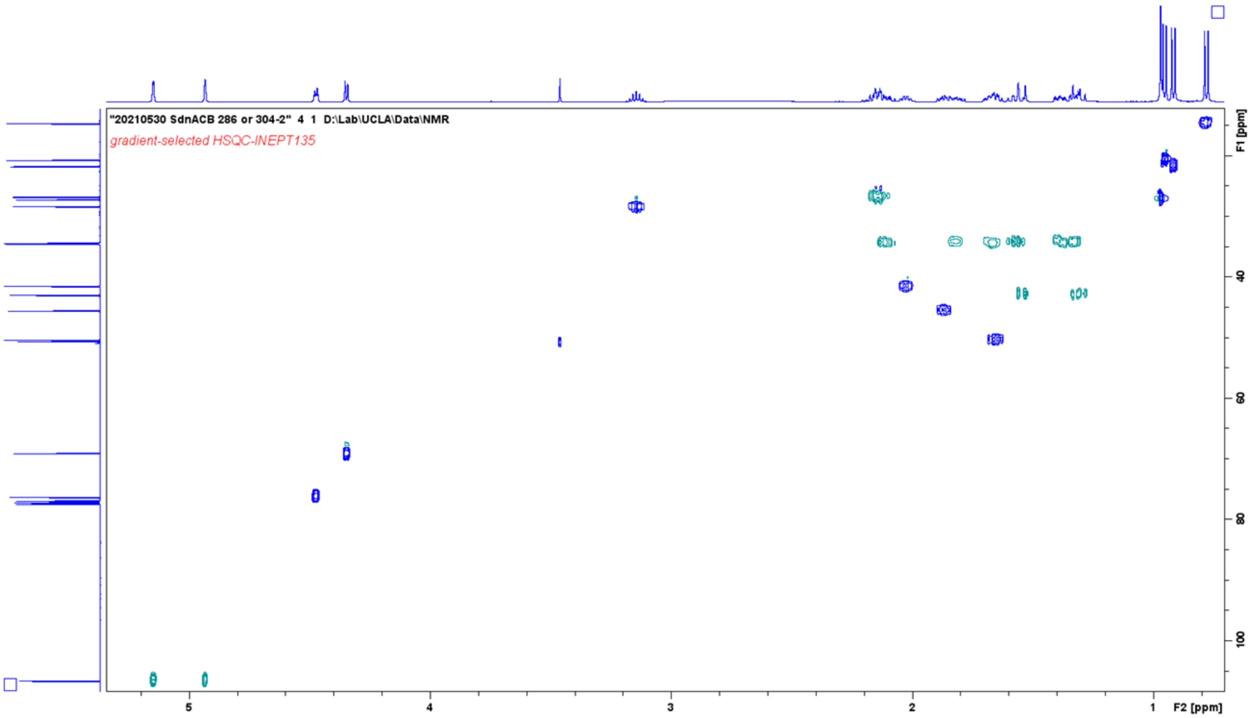

Supplementary Fig 17. ^{1}H-^{13}C HSQC of compound **2** in $CDCl_3$, 500 MHz.

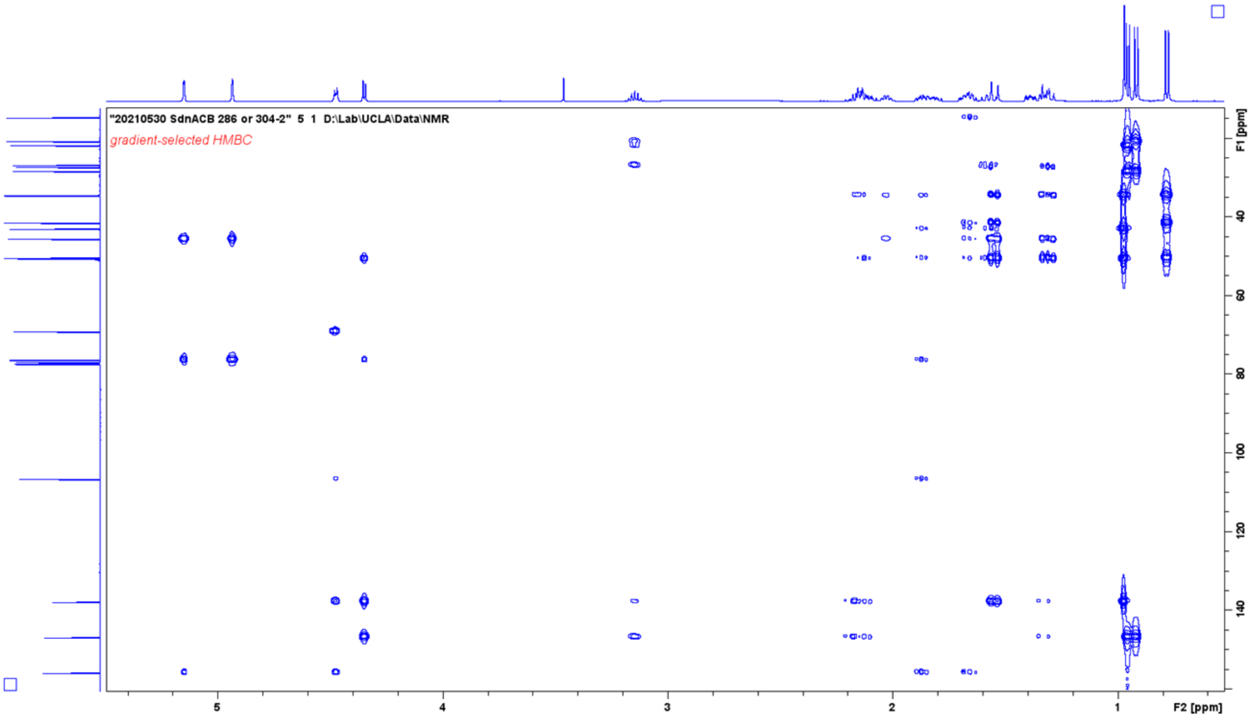

Supplementary Fig 18. ^{1}H-^{13}C HMBC of compound **2** in $CDCl_3$, 500 MHz.

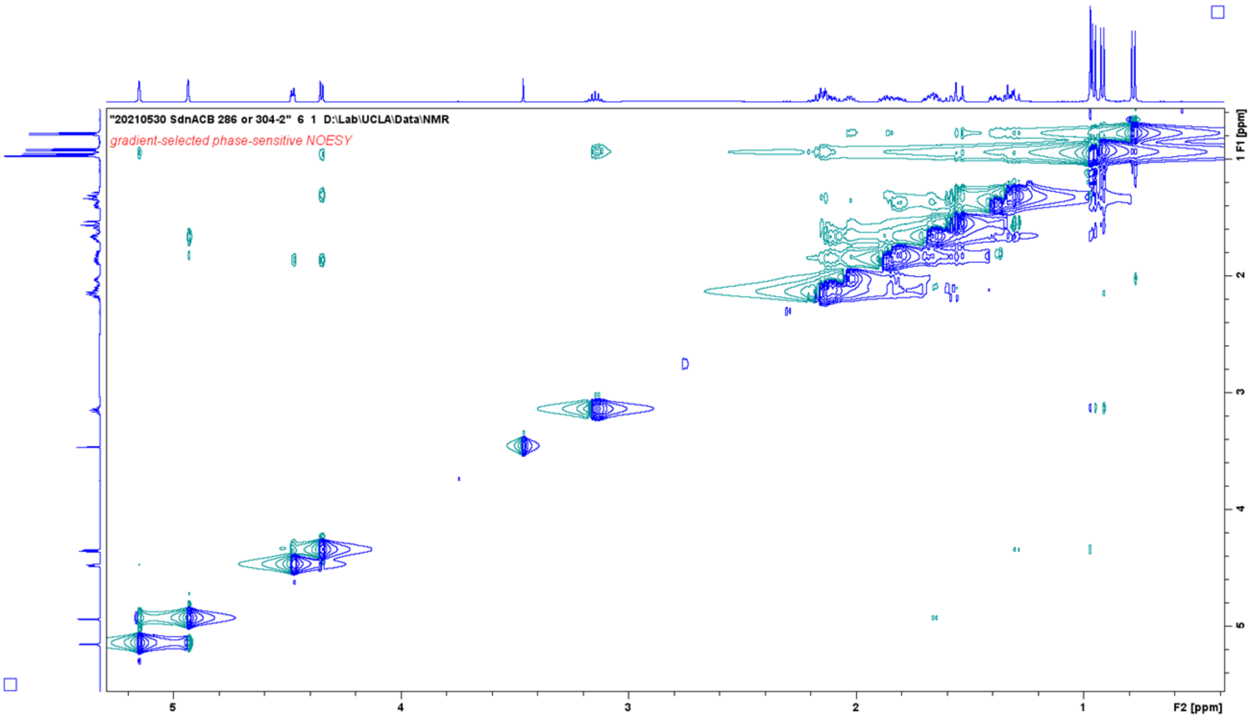

Supplementary Fig 19. ^{1}H-^{1}H NOESY of compound **2** in $CDCl_3$, 500 MHz.

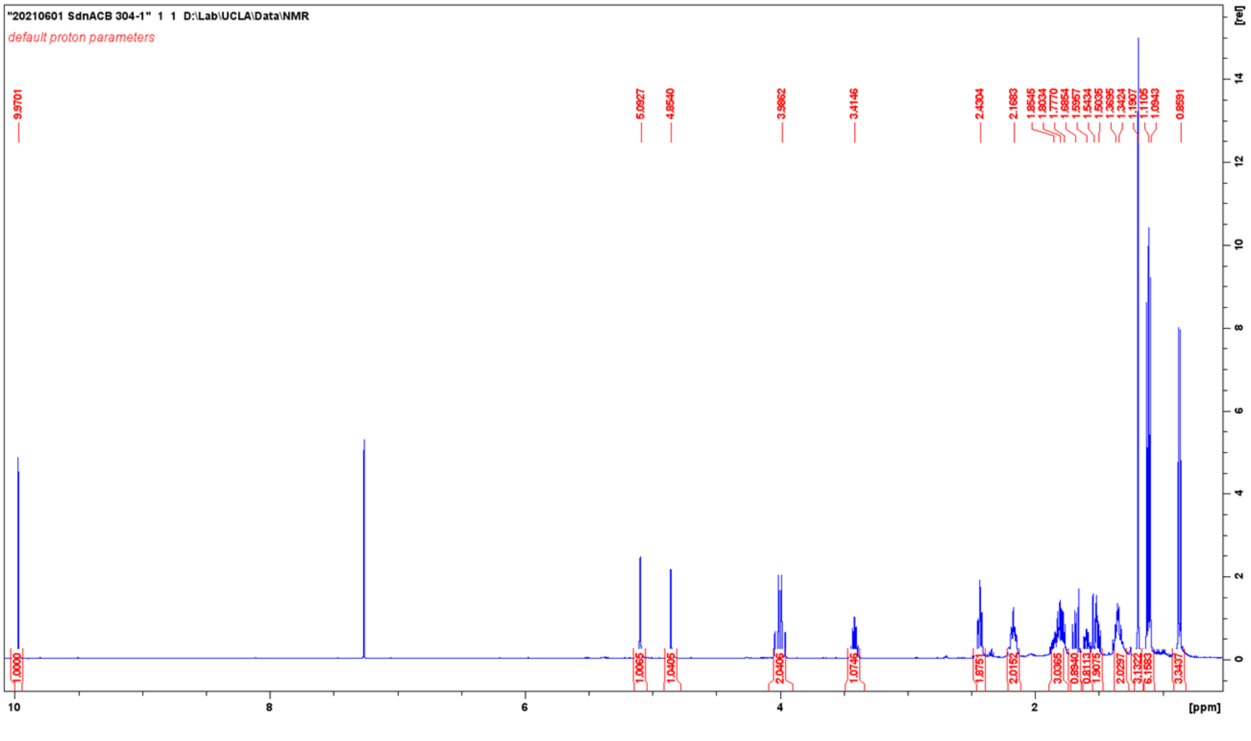

Supplementary Fig 20. ^{1}H NMR of compound **3** in $CDCl_3$, 500 MHz.

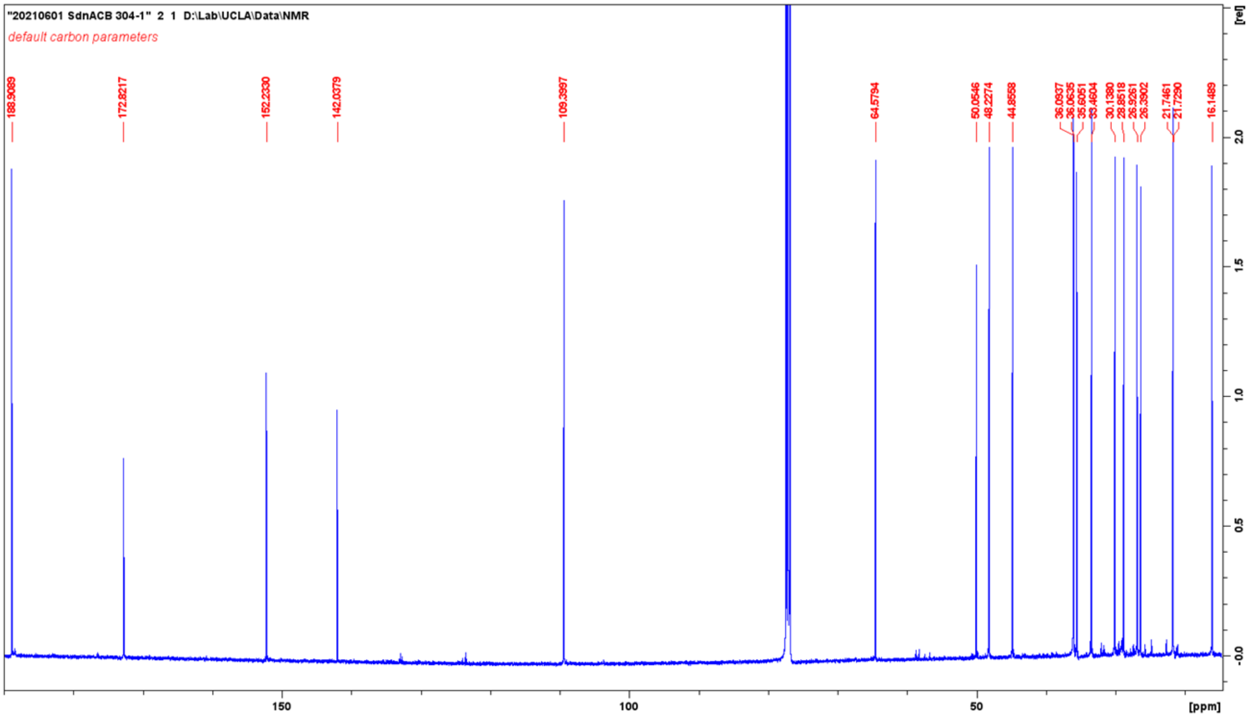

Supplementary Fig 21. ^{13}C NMR of compound **3** in $CDCl_3$, 500 MHz.

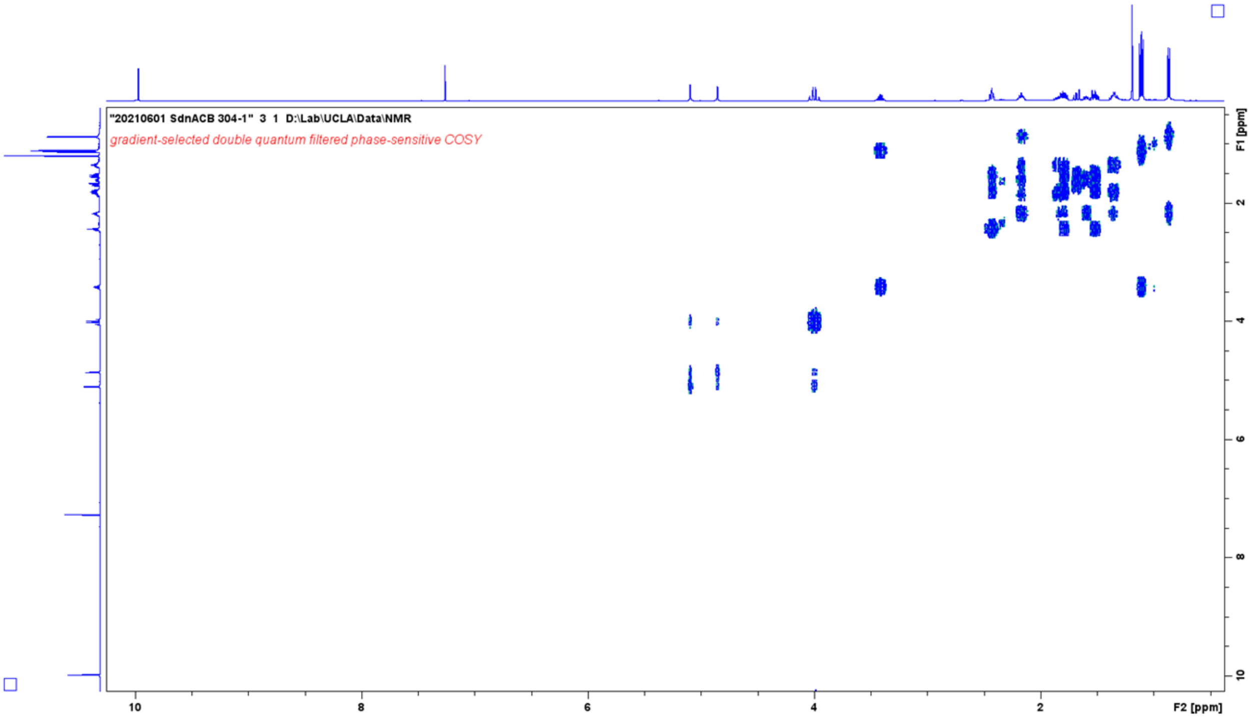

Supplementary Fig 22. ^{1}H-^{1}H COSY of compound **3** in $CDCl_3$, 500 MHz.

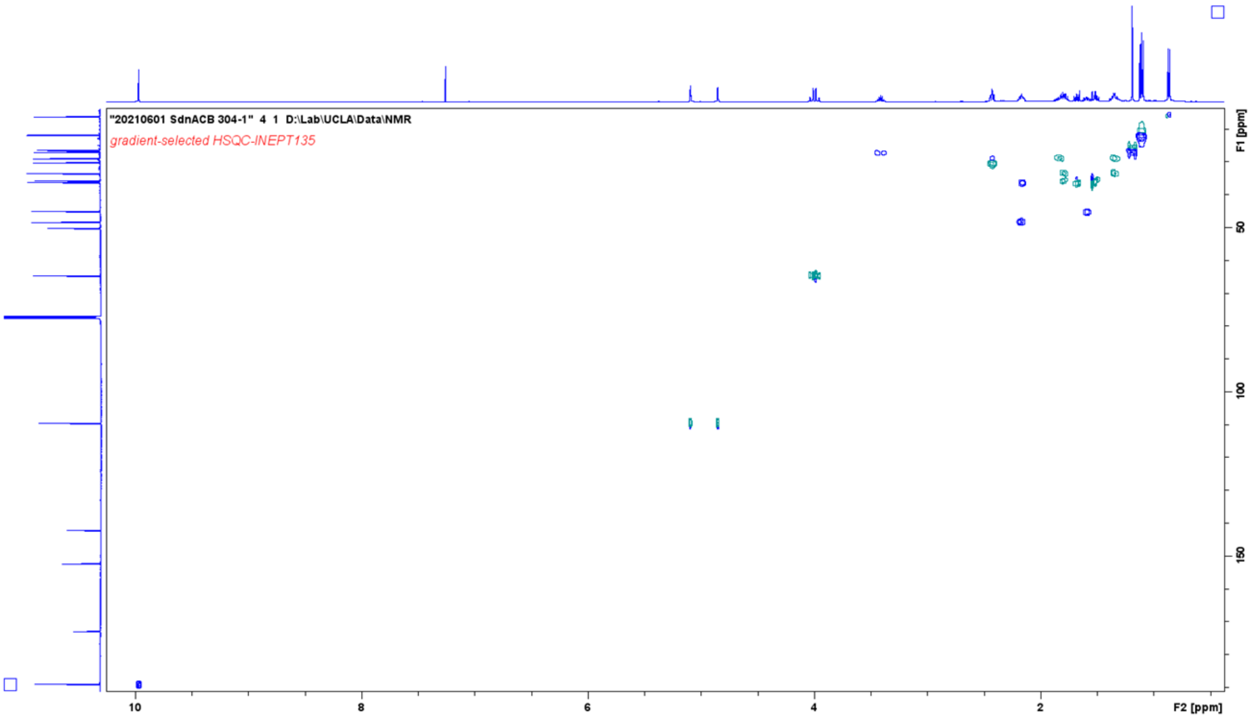

Supplementary Fig 23. ^{1}H-^{13}C HSQC of compound **3** in $CDCl_3$, 500 MHz.

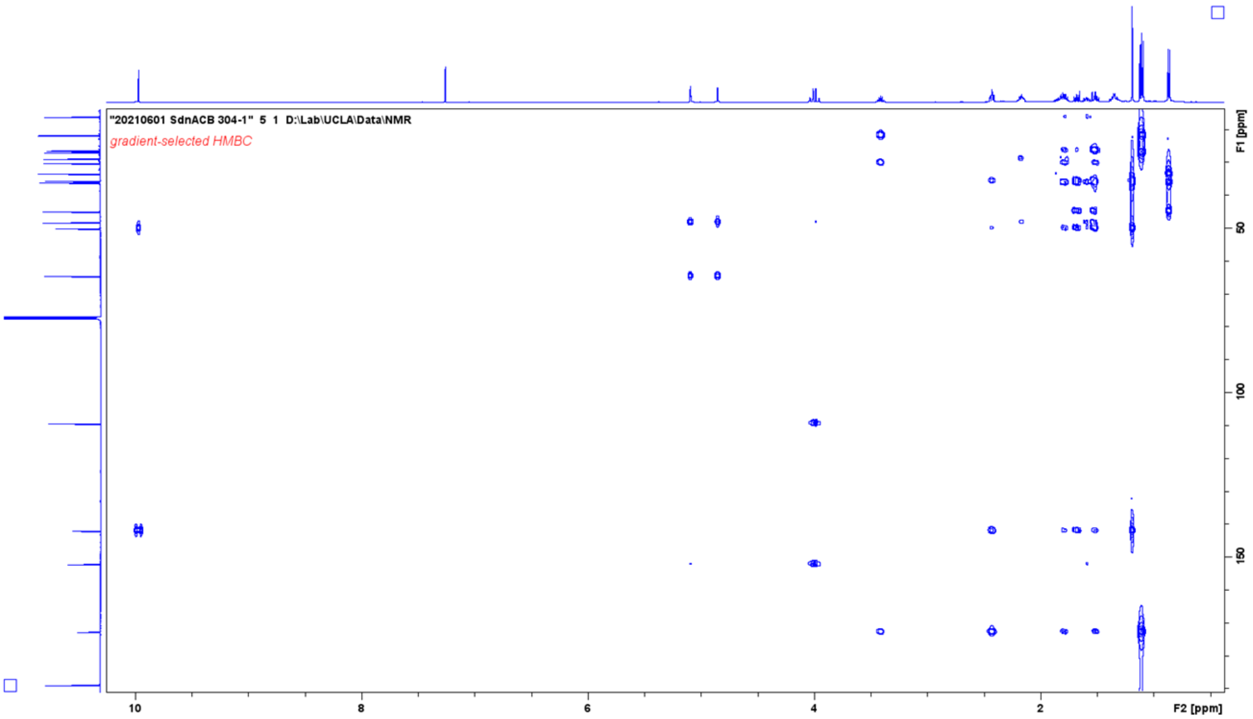

Supplementary Fig 24. ^{1}H-^{13}C HMBC of compound **3** in $CDCl_3$, 500 MHz.

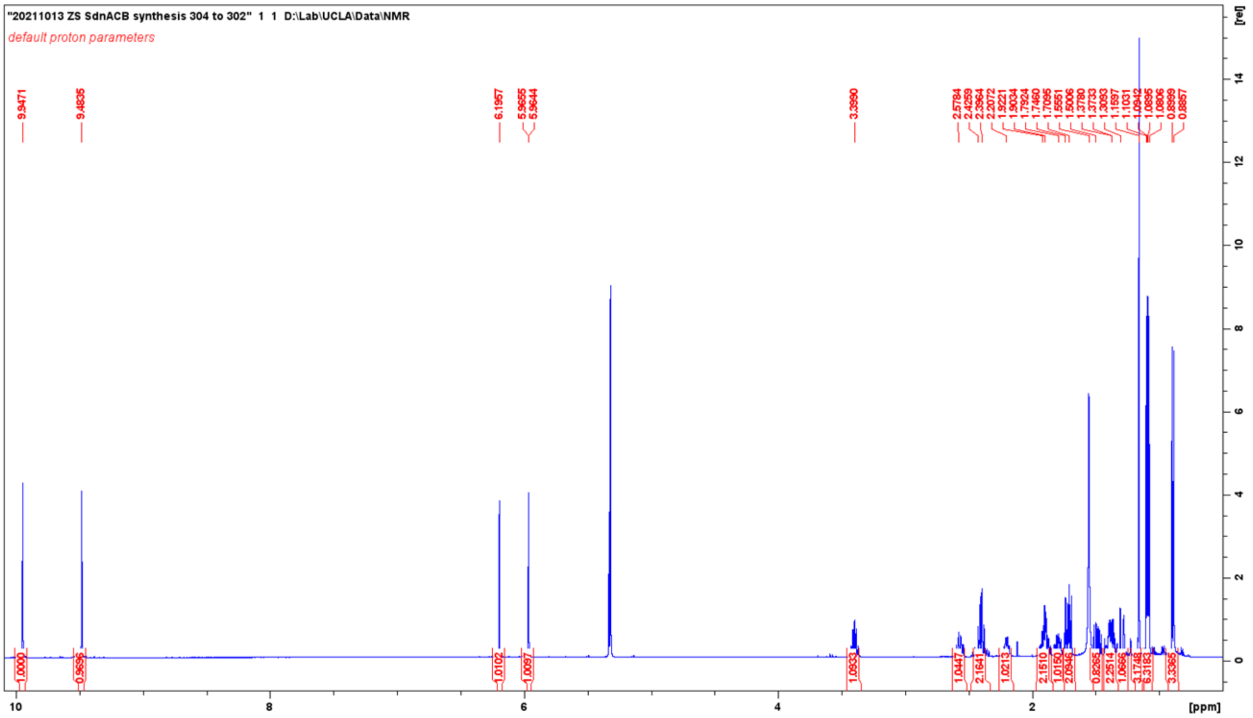

Supplementary Fig 25. ^{1}H NMR of compound **4** in CD_2Cl_2, 500 MHz.

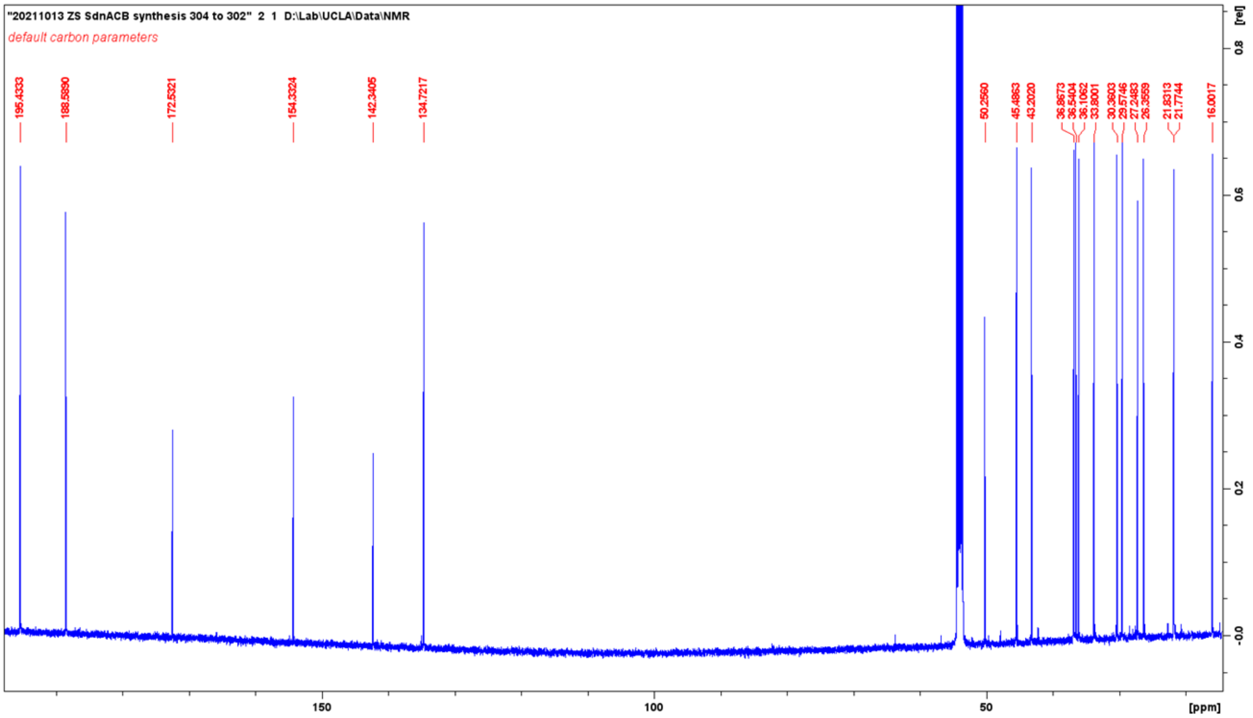

Supplementary Fig 26. ^{13}C NMR of compound **4** in CD_2Cl_2, 500 MHz.

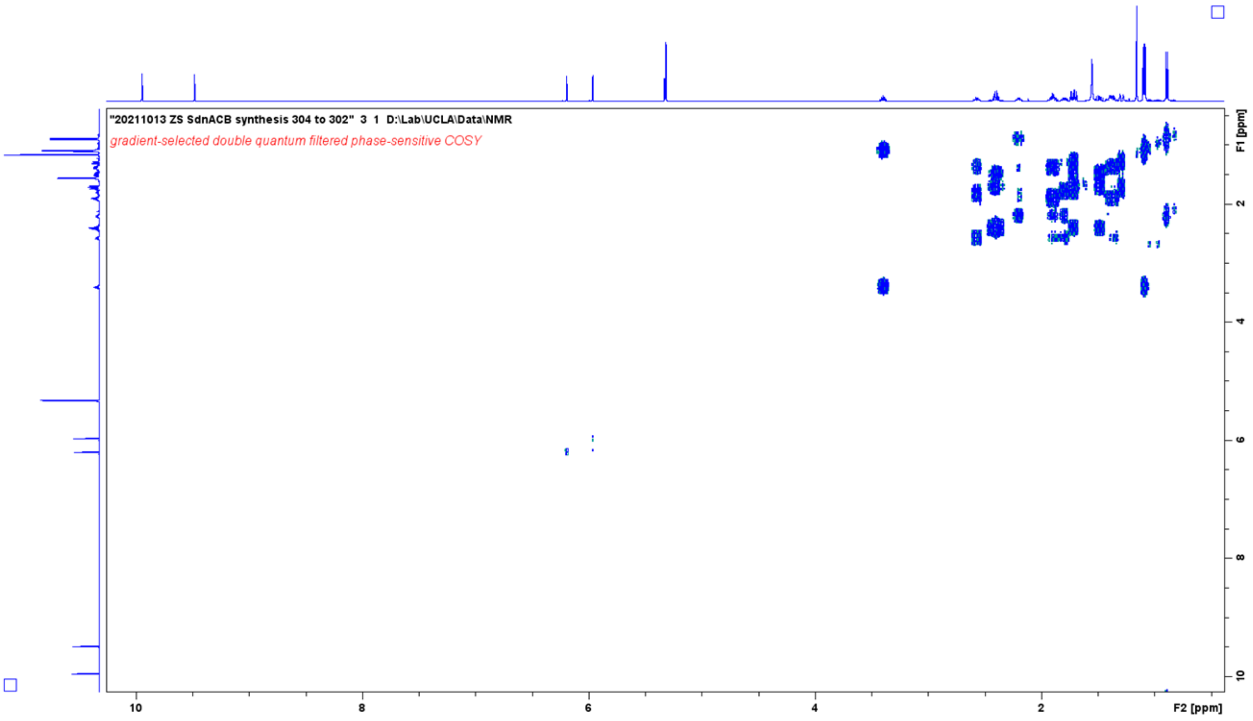

Supplementary Fig 27. ^{1}H-^{1}H COSY of compound **4** in CD_2Cl_2, 500 MHz.

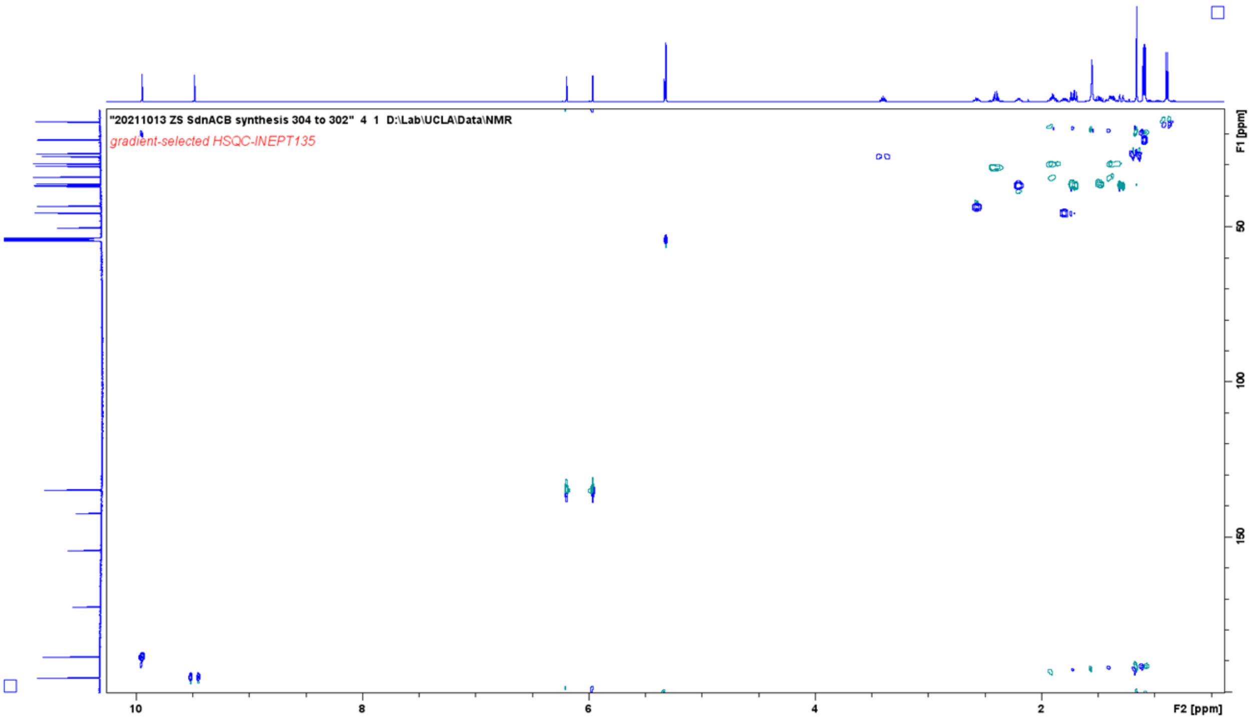

Supplementary Fig 28. ^{1}H-^{13}C HSQC of compound **4** in CD_2Cl_2, 500 MHz.

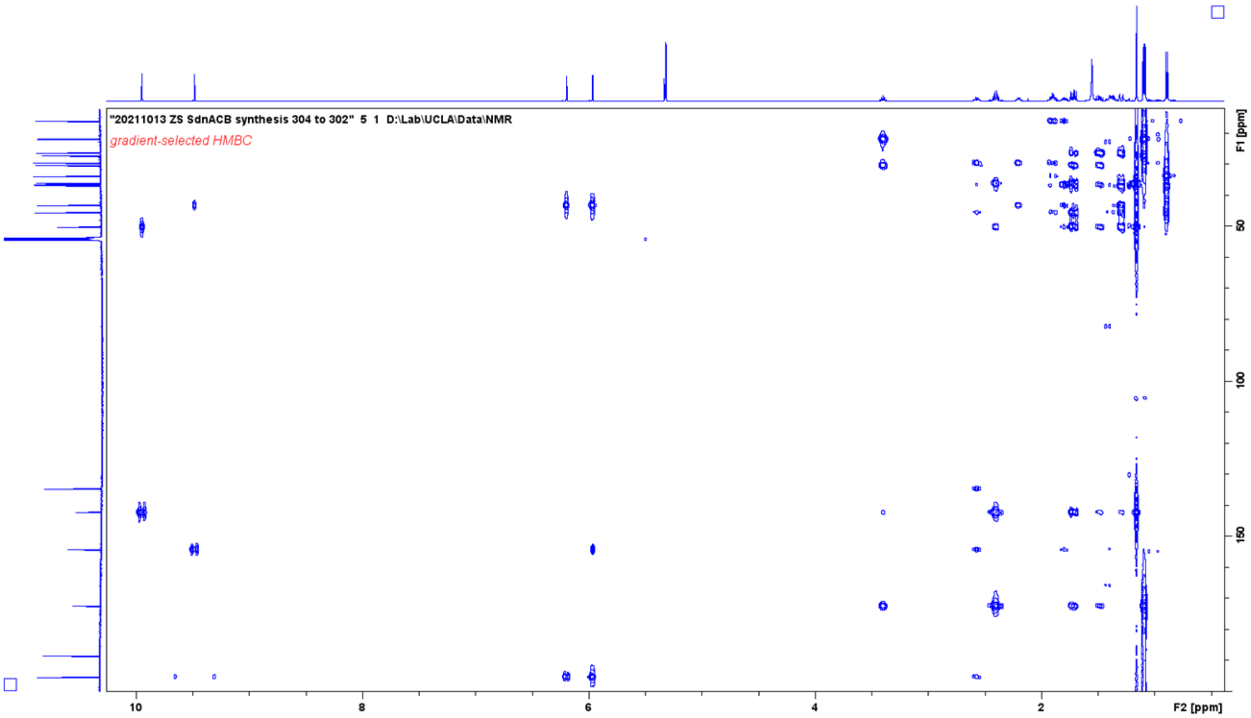

Supplementary Fig 29. ^{1}H-^{13}C HMBC of compound **4** in CD_2Cl_2, 500 MHz.

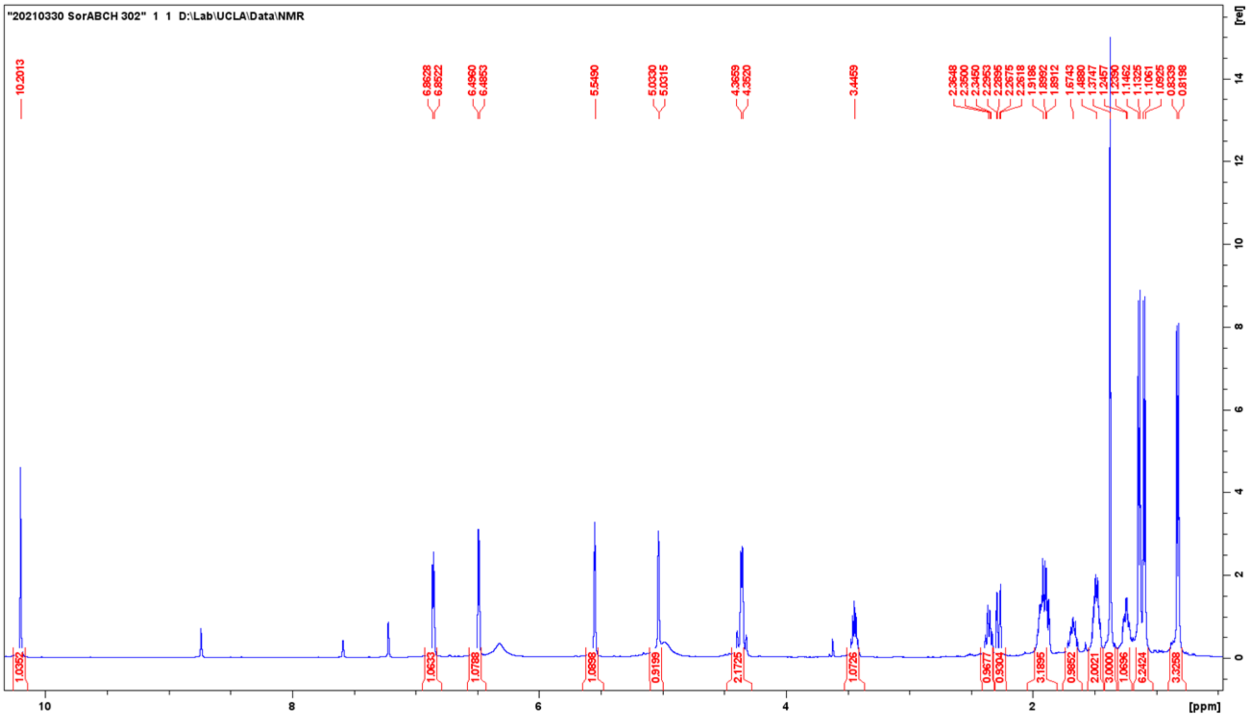

Supplementary Fig 30. ^{1}H NMR of compound **5** in d5-pyridine, 500 MHz.

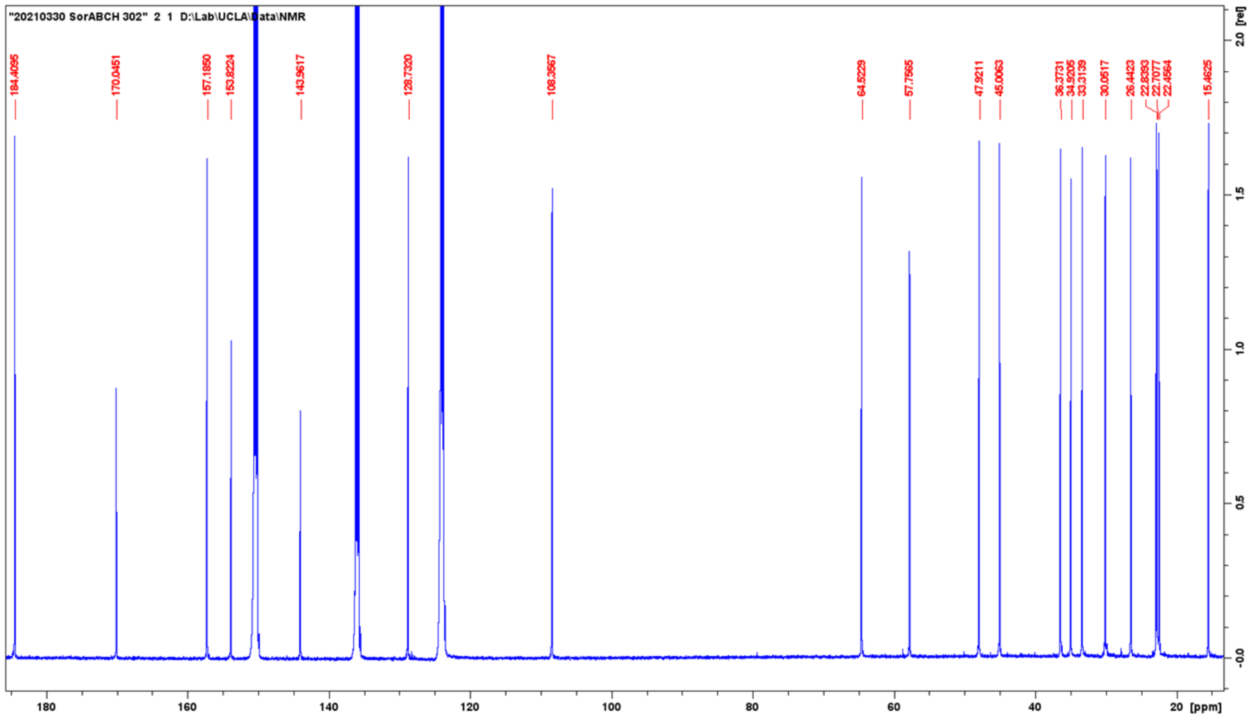

Supplementary Fig 31. ^{13}C NMR of compound **5** in d5-pyridine, 500 MHz.

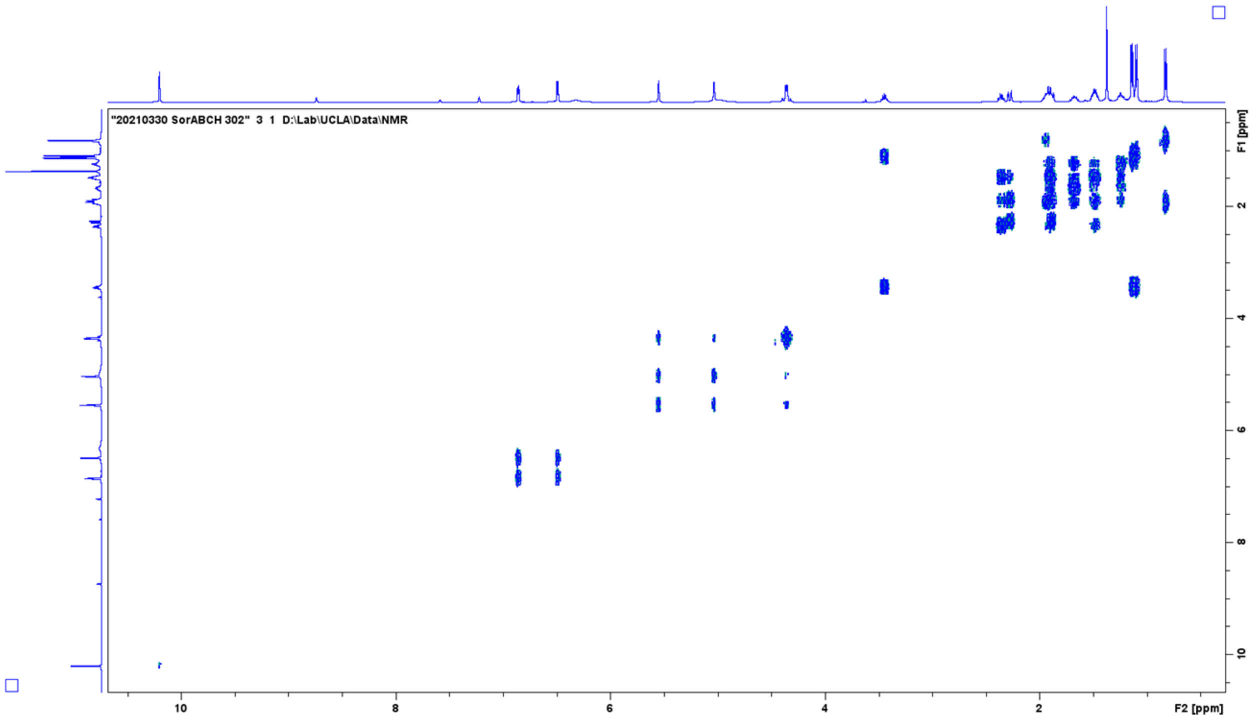

Supplementary Fig 32. ^{1}H-^{1}H COSY of compound **5** in d5-pyridine, 500 MHz.

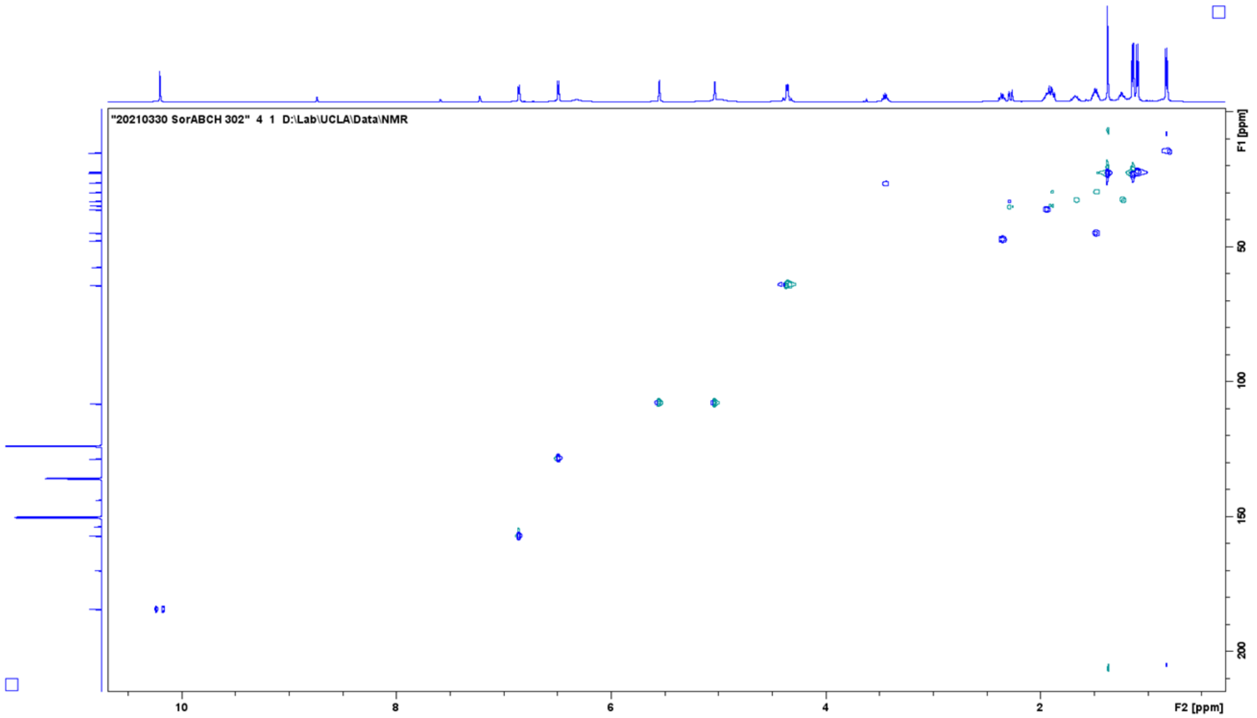

Supplementary Fig 33. ^{1}H-^{13}C HSQC of compound **5** in d5-pyridine, 500 MHz.

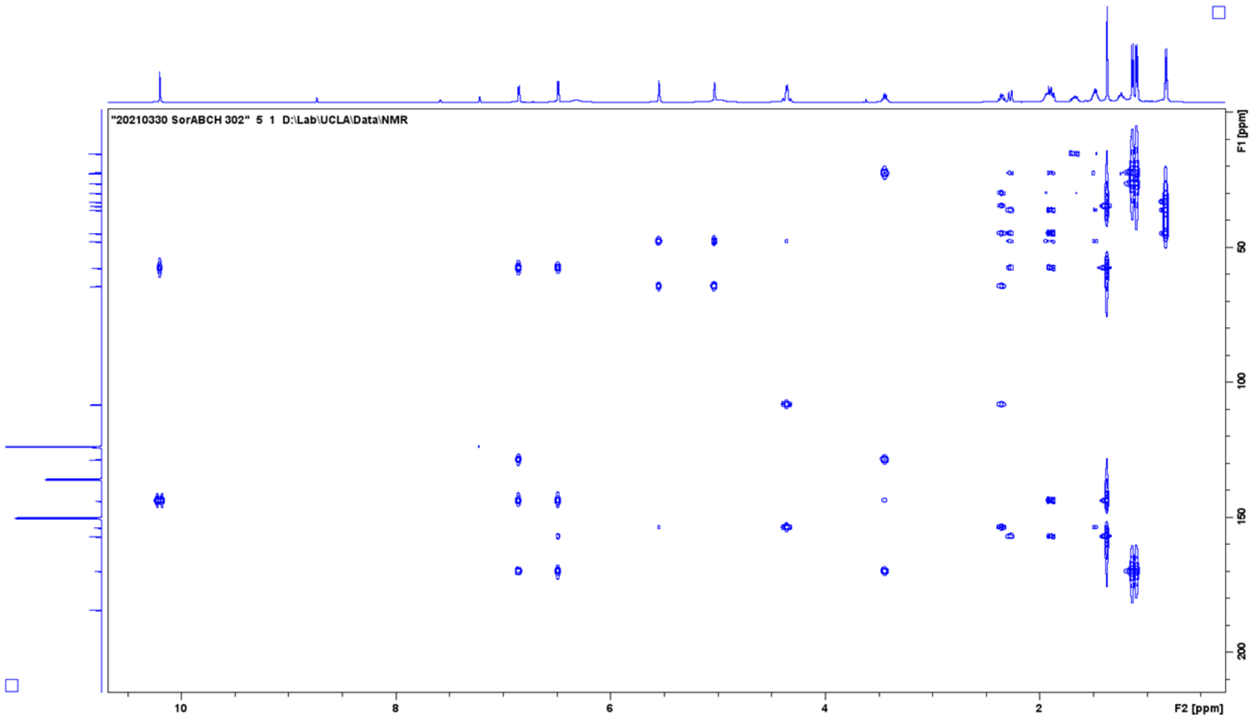

Supplementary Fig 34. ^{1}H-^{13}C HMBC of compound **5** in d5-pyridine, 500 MHz.

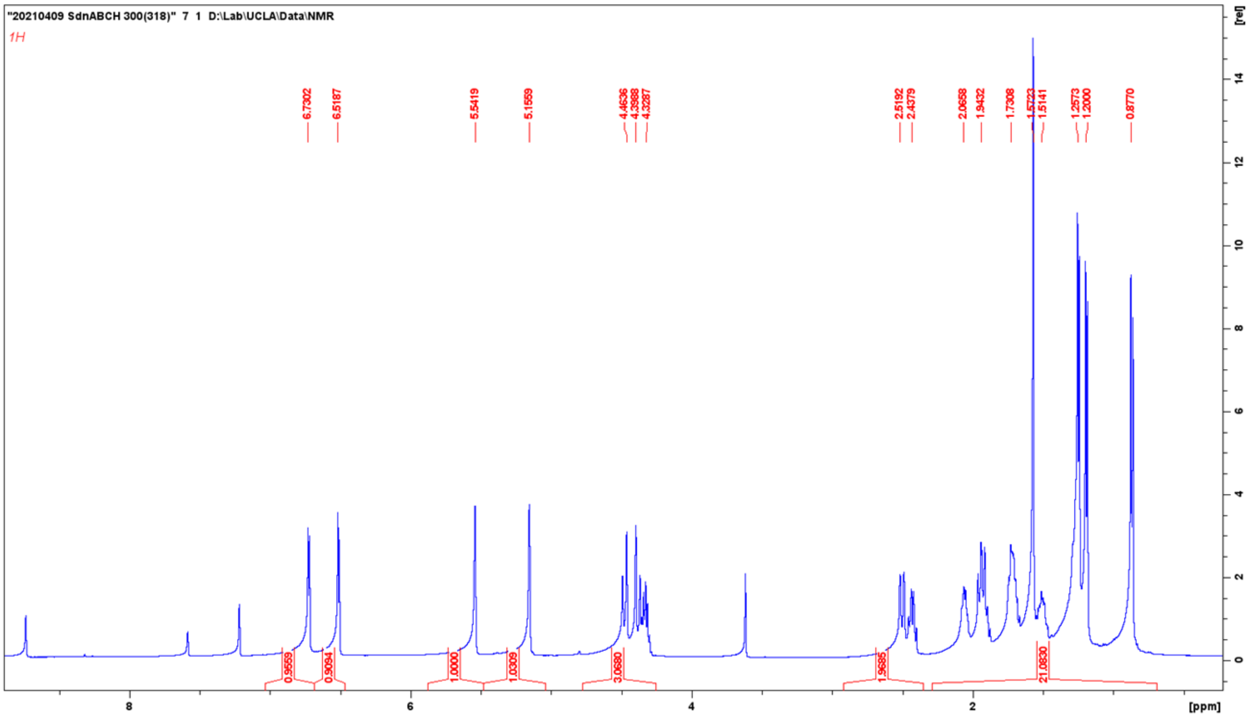

Supplementary Fig 35. ^{1}H NMR of compound **6** in d5-pyridine, 500 MHz.

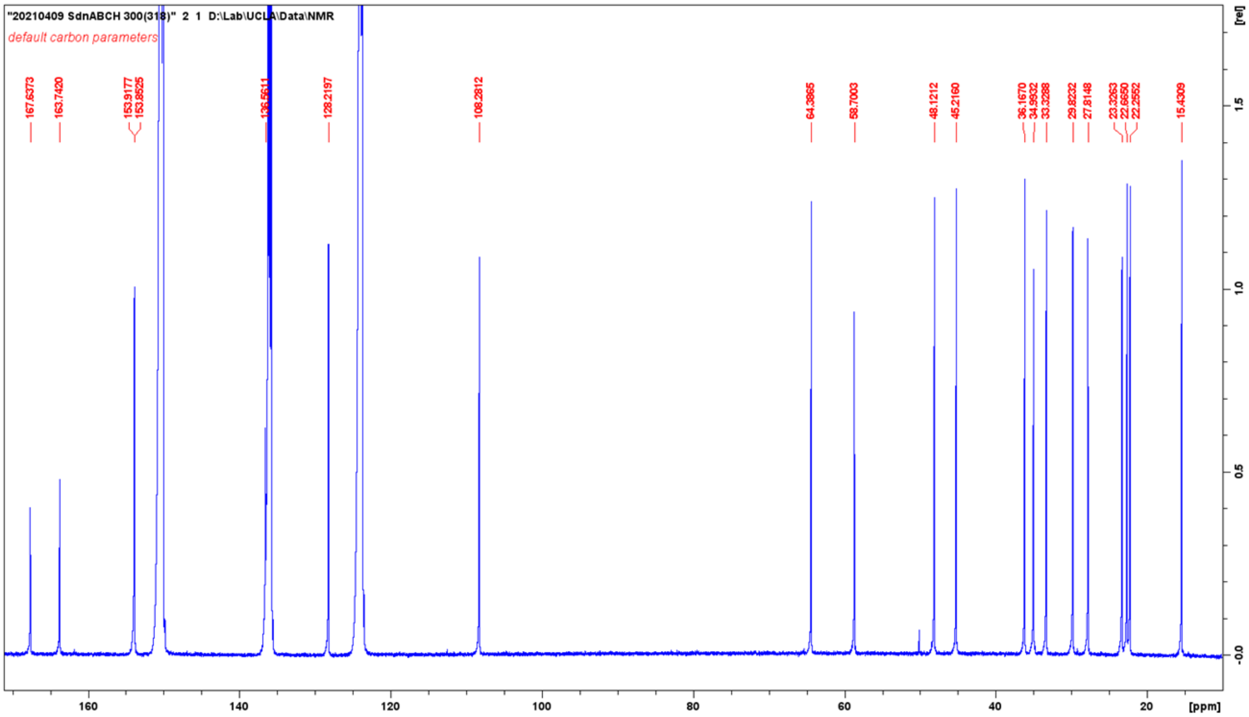

Supplementary Fig 36. ^{13}C NMR of compound **6** in d5-pyridine, 500 MHz.

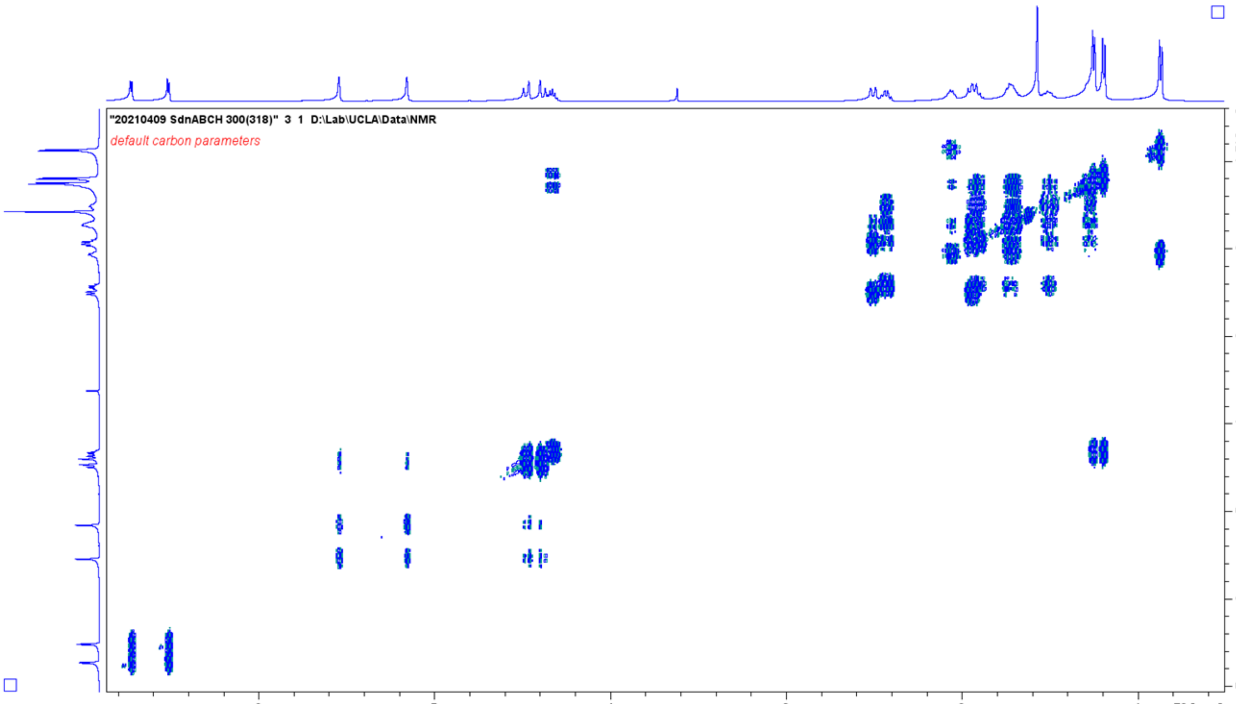

Supplementary Fig 37. ^{1}H-^{1}H COSY of compound **6** in d5-pyridine, 500 MHz.

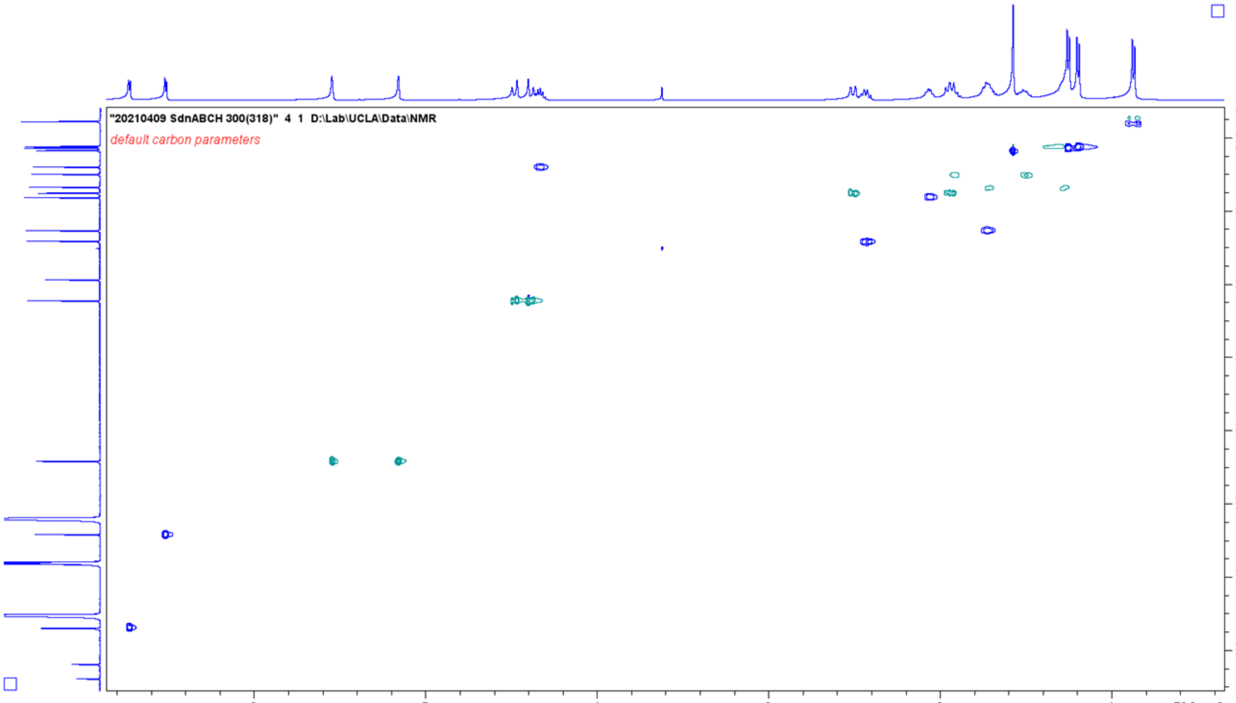

Supplementary Fig 38. ^{1}H-^{13}C HSQC of compound **6** in d5-pyridine, 500 MHz.

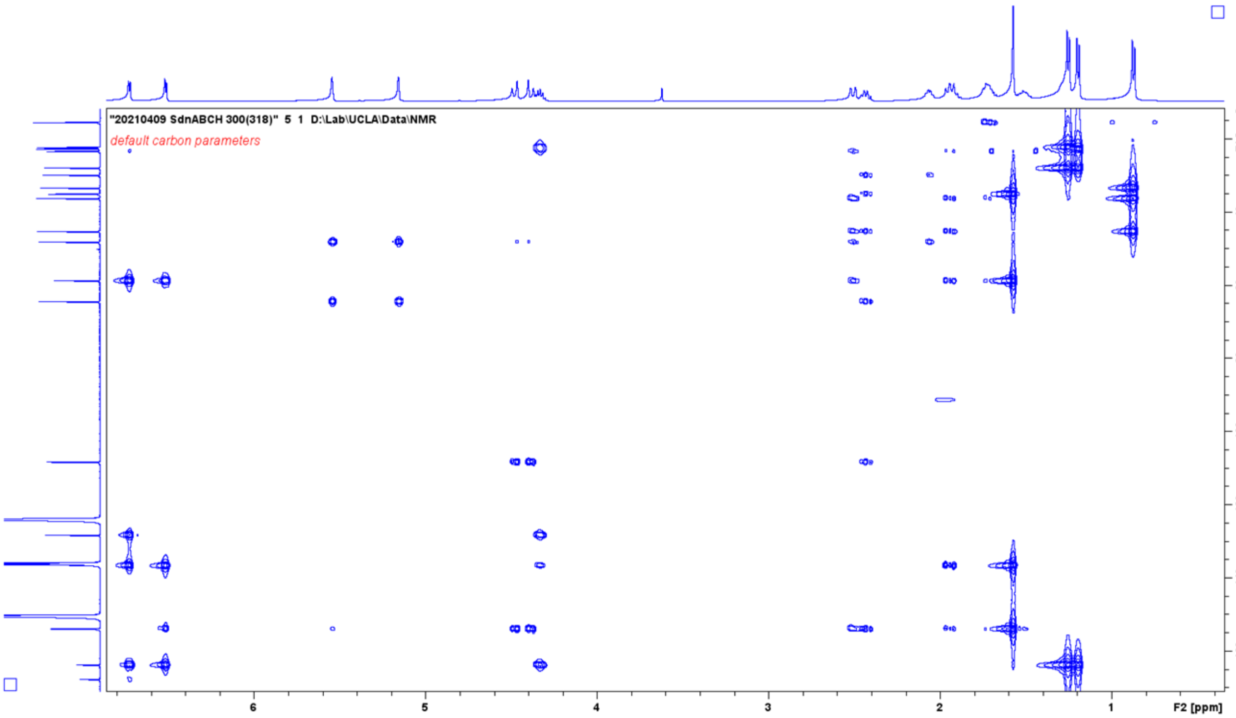

Supplementary Fig 39. ^{1}H-^{13}C HMBC of compound **6** in d5-pyridine, 500 MHz.

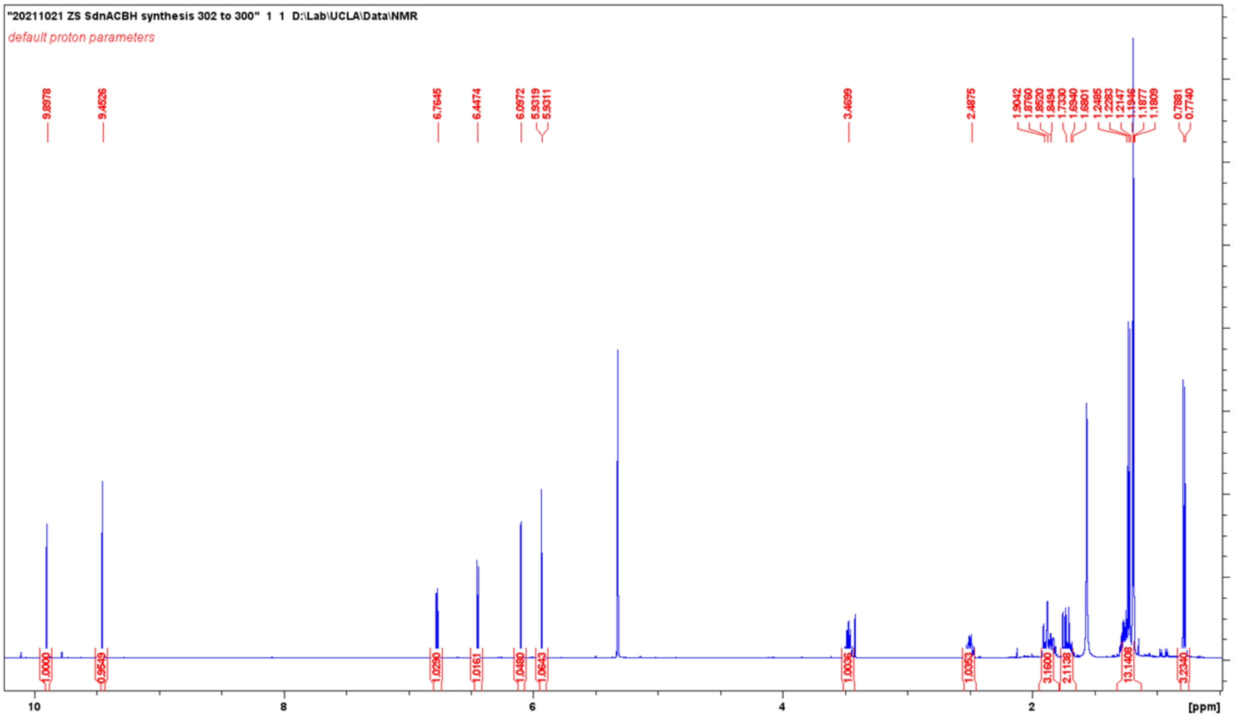

Supplementary Fig 40. ^{1}H NMR of compound **7** in CD_2Cl_2, 500 MHz.

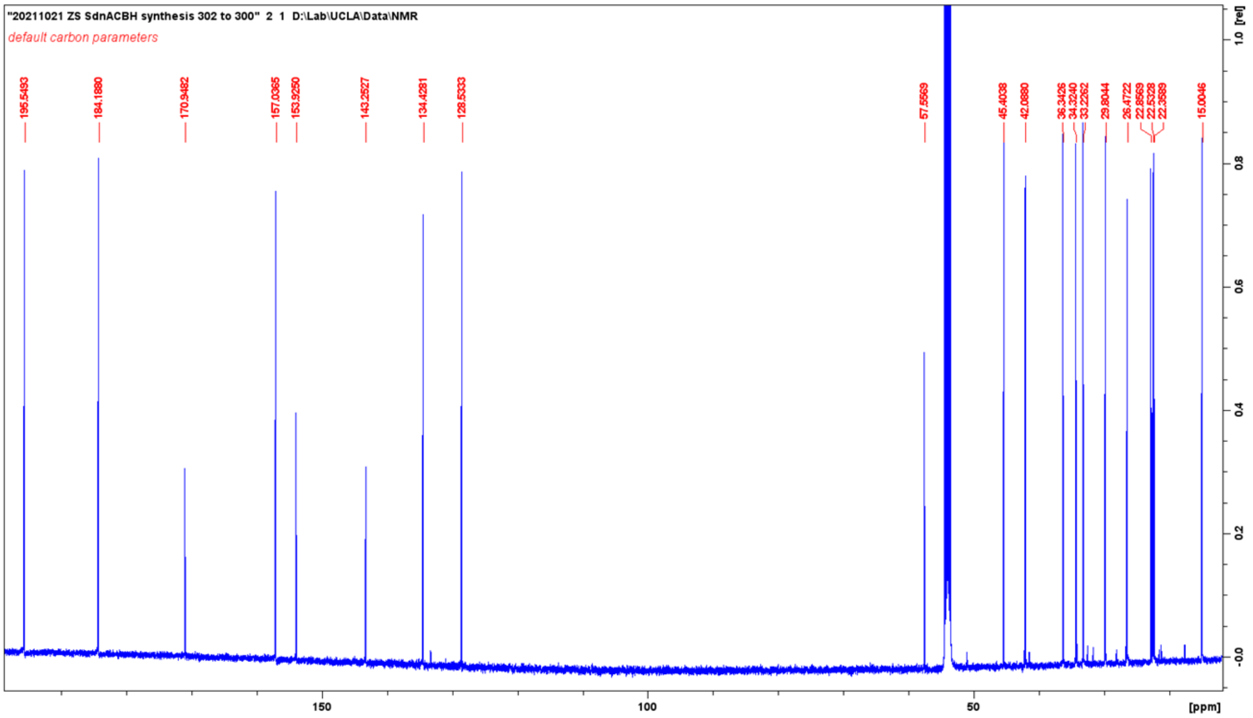

Supplementary Fig 41. ^{13}C NMR of compound **7** in CD_2Cl_2, 500 MHz.

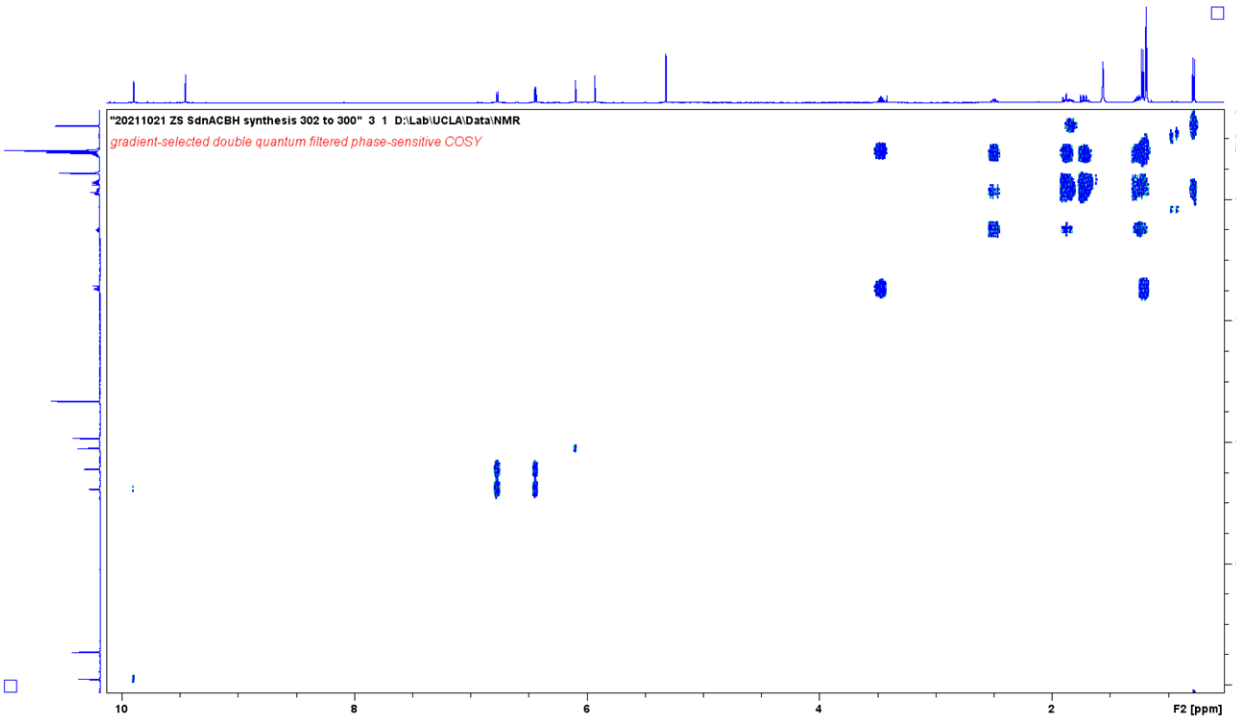

Supplementary Fig 42. ^{1}H-^{1}H COSY of compound **7** in CD_2Cl_2, 500 MHz.

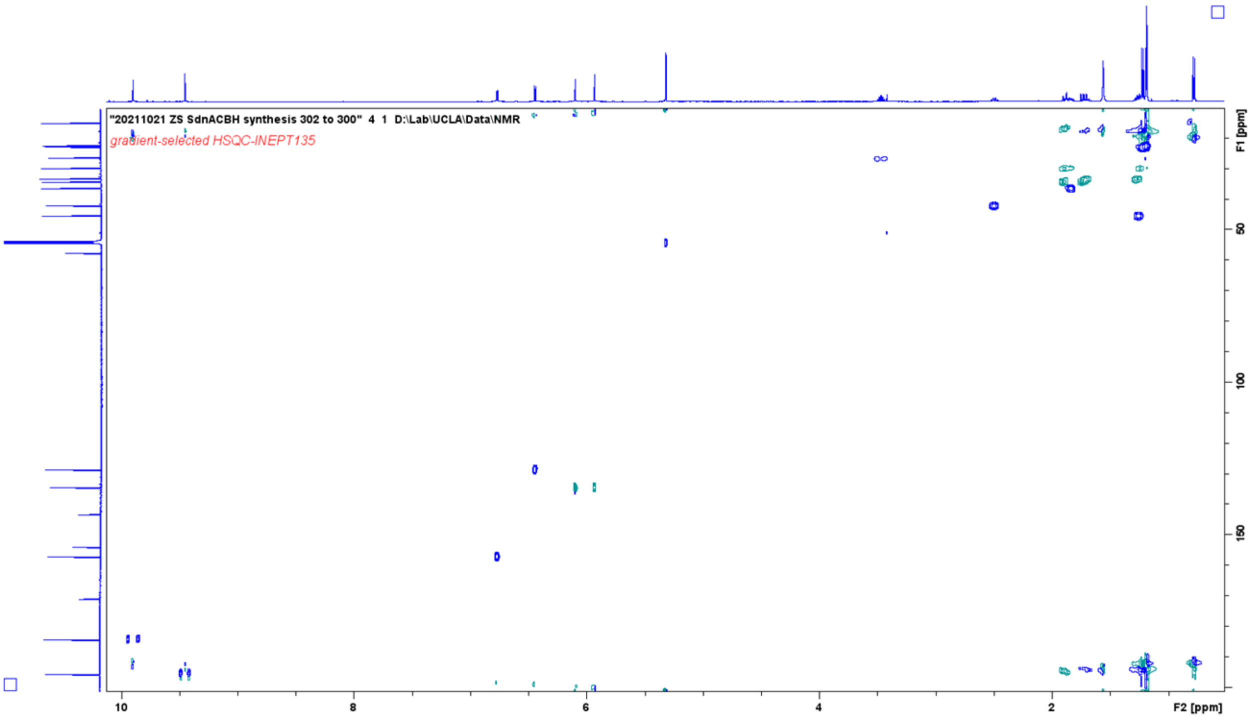

Supplementary Fig 43. ^{1}H-^{13}C HSQC of compound **7** in CD_2Cl_2, 500 MHz.

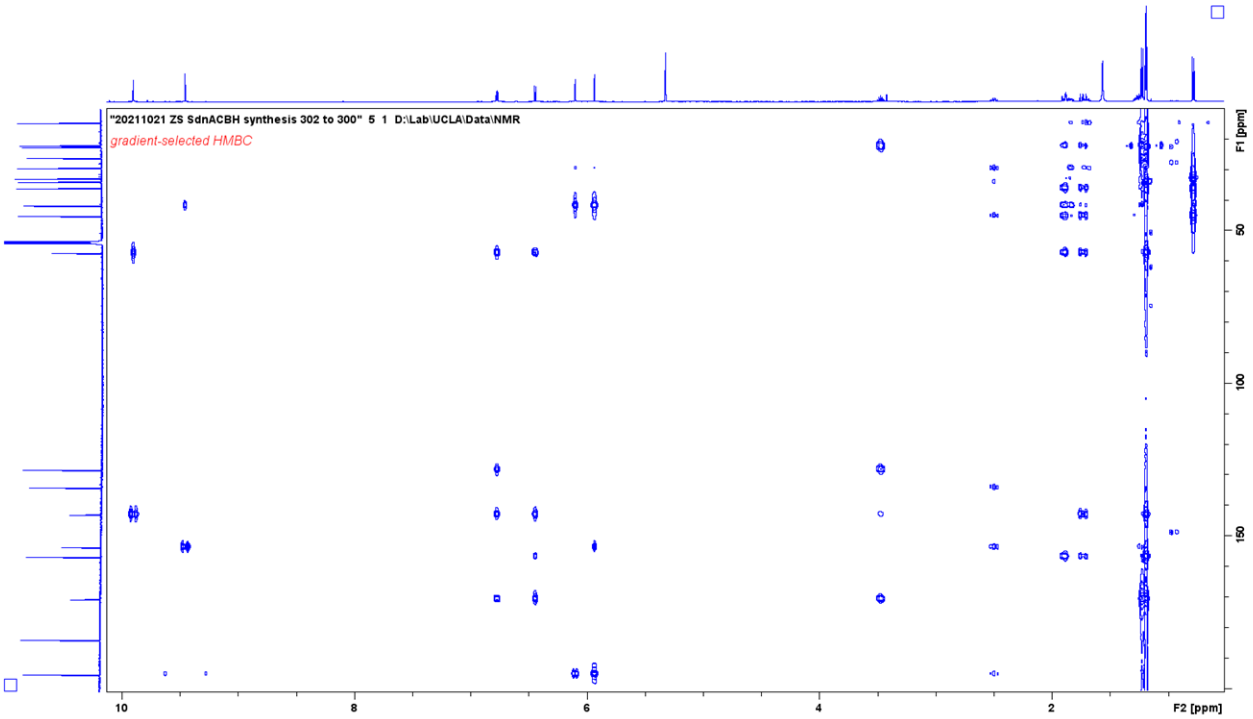

Supplementary Fig 44. ^{1}H-^{13}C HMQC of compound **7** in CD_2Cl_2, 500 MHz.

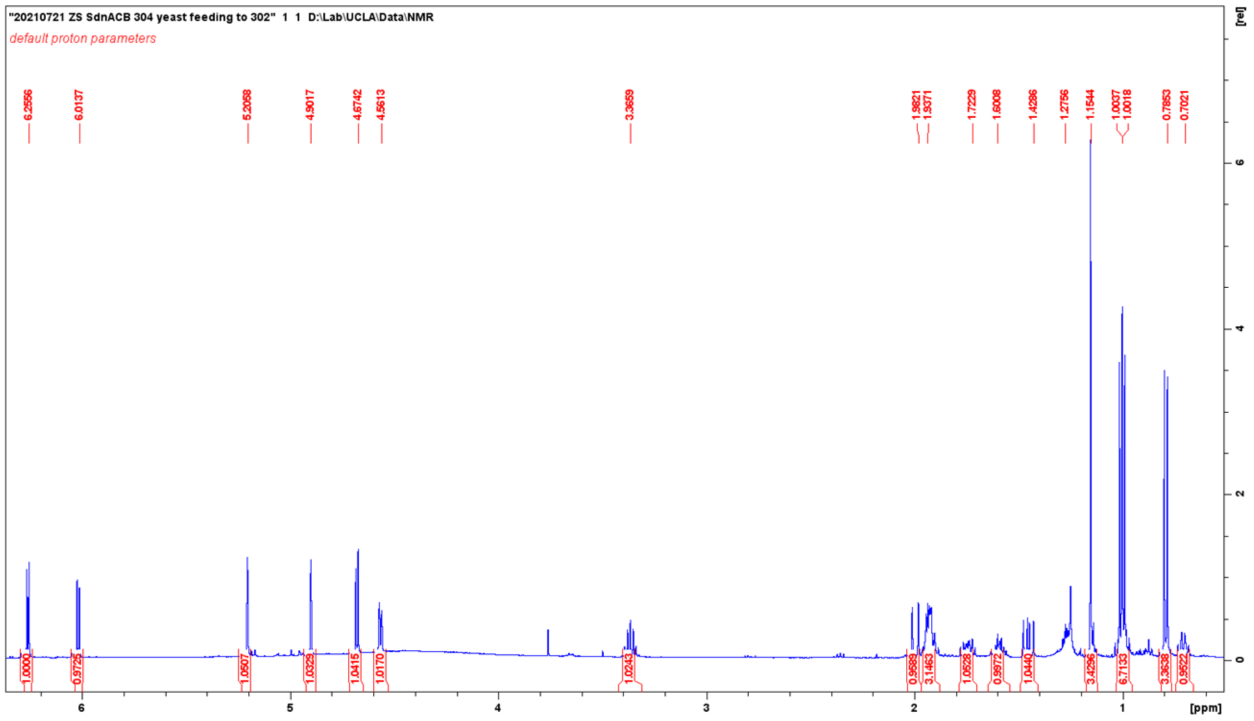

Supplementary Fig 45. ^{1}H NMR of compound **8** in $CDCl_3$, 500 MHz.

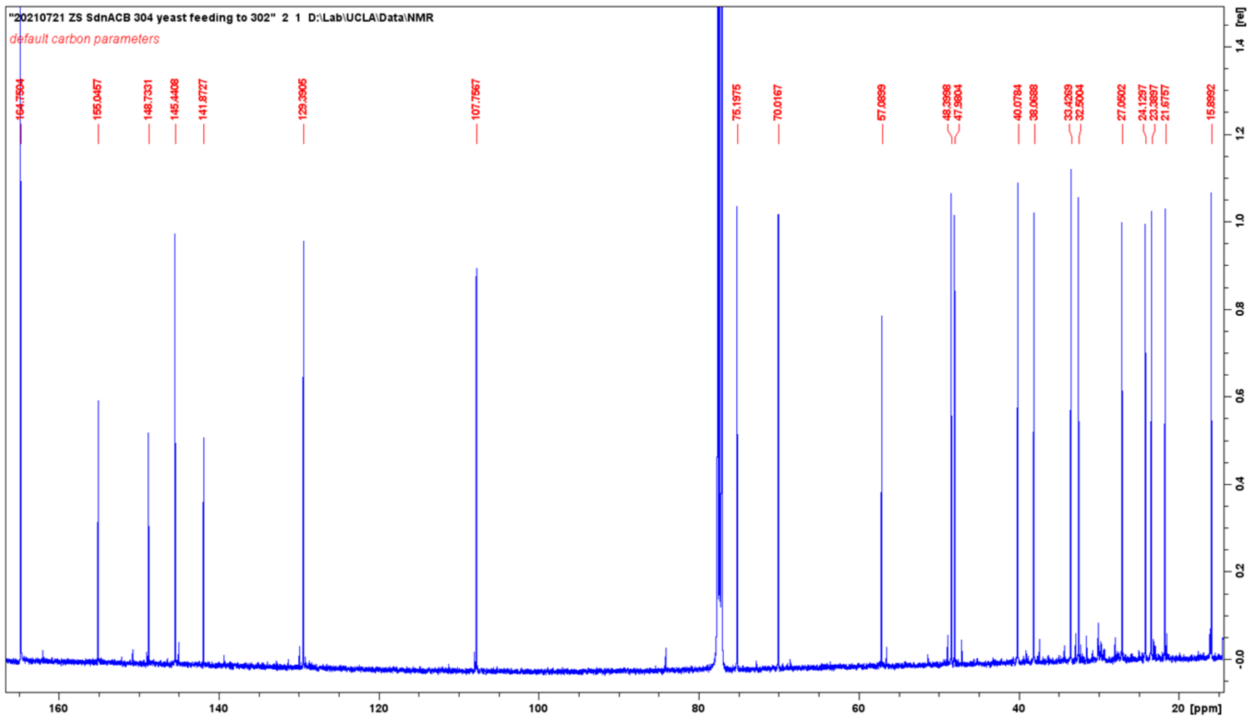

Supplementary Fig 46. ^{13}C NMR of compound **8** in $CDCl_3$, 500 MHz.

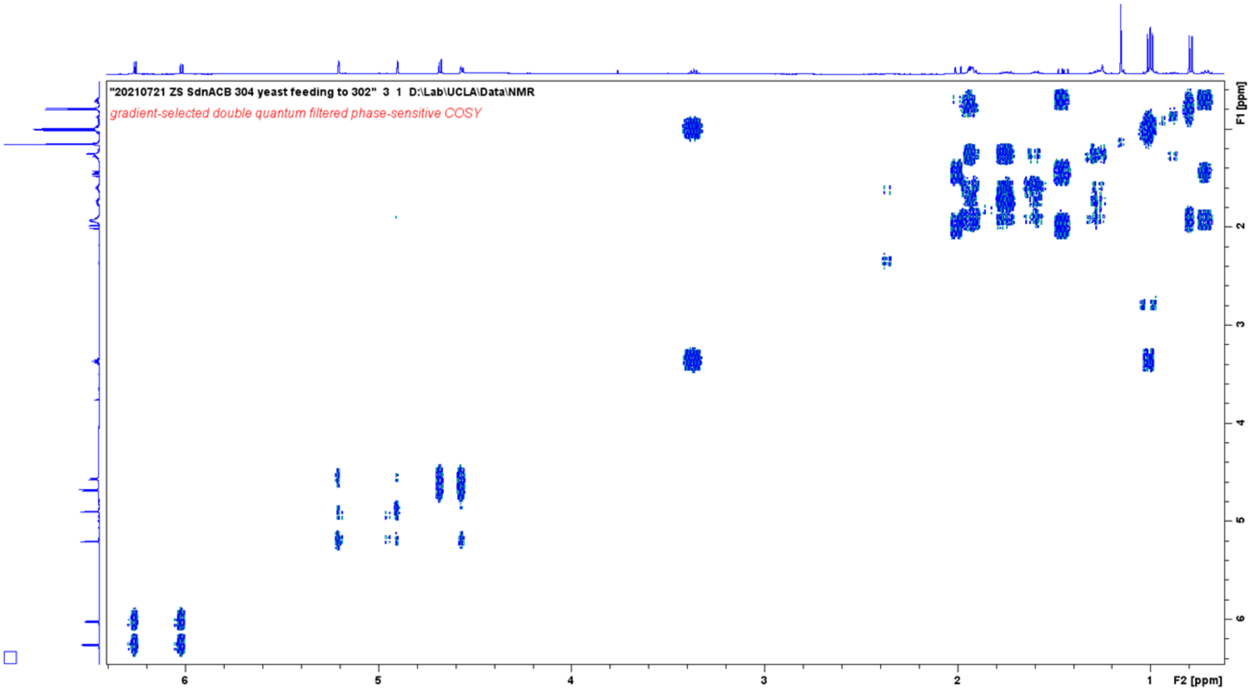

Supplementary Fig 47. ^{1}H-^{1}H COSY of compound **8** in $CDCl_3$, 500 MHz.

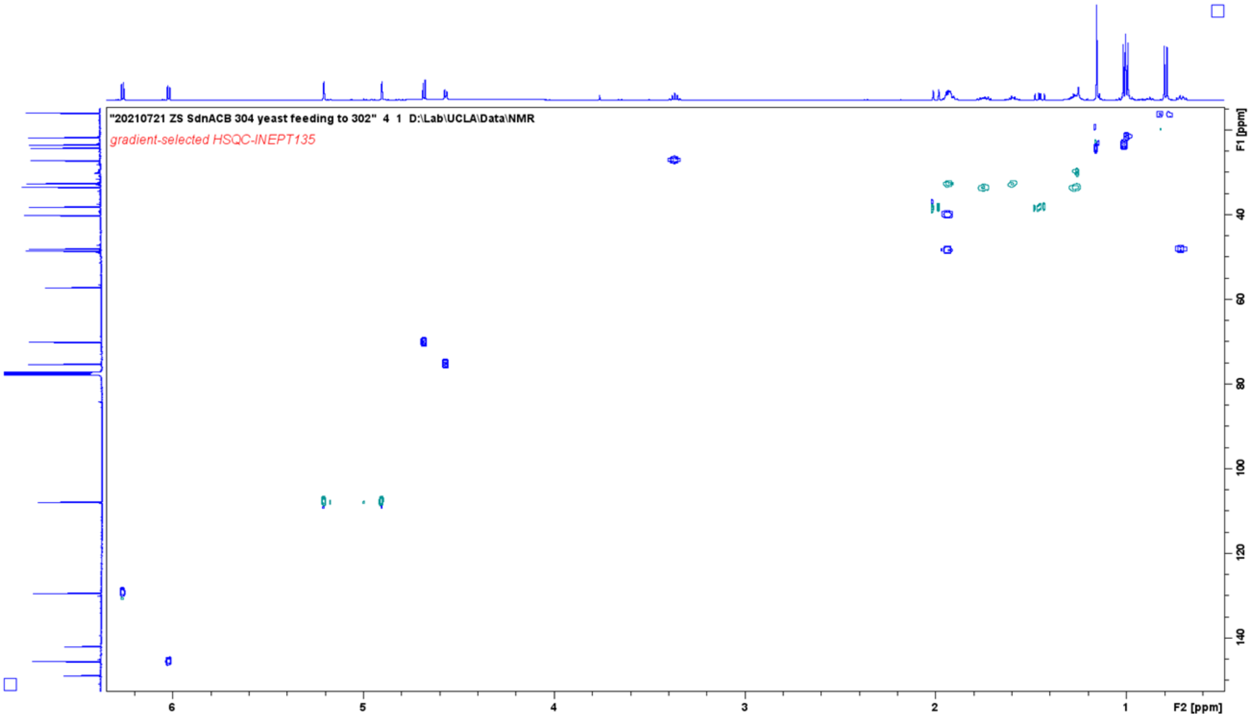

Supplementary Fig 48. ^{1}H-^{13}C HSQC of compound **8** in $CDCl_3$, 500 MHz.

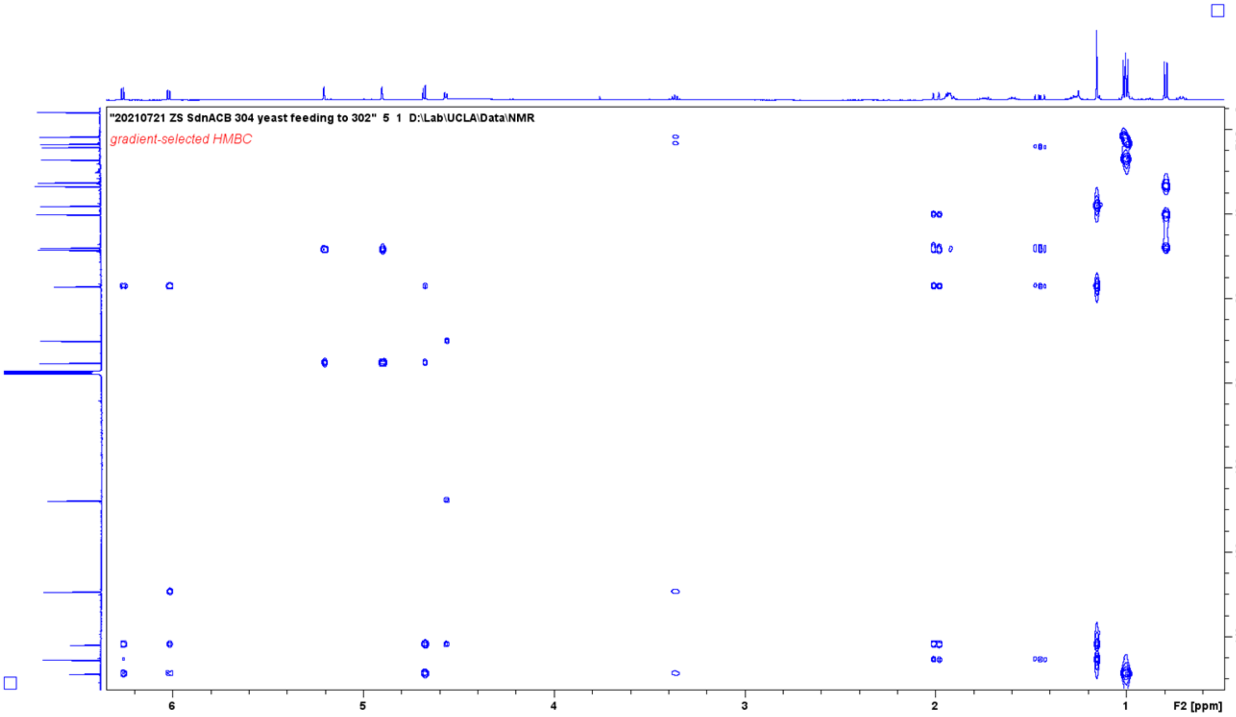

Supplementary Fig 49. ^{1}H-^{13}C HMBC of compound **8** in $CDCl_3$, 500 MHz.

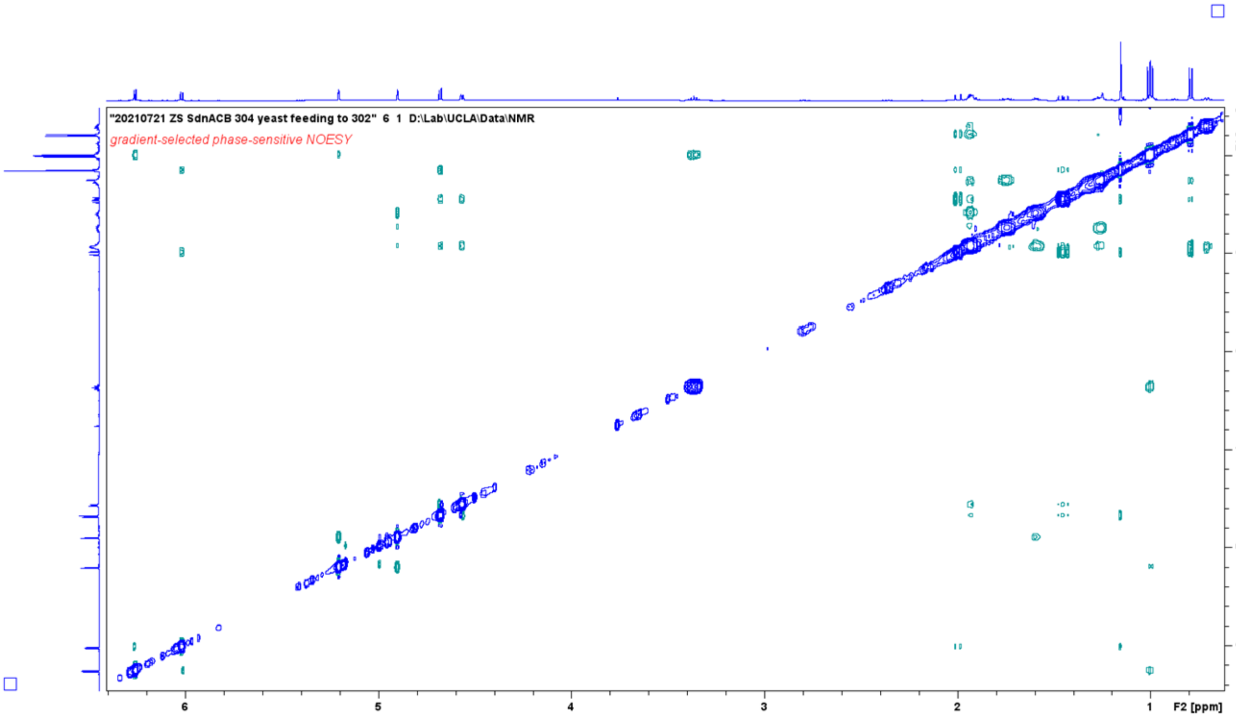

Supplementary Fig 50. ^{1}H-^{1}H NOESY of compound **8** in $CDCl_3$, 500 MHz.

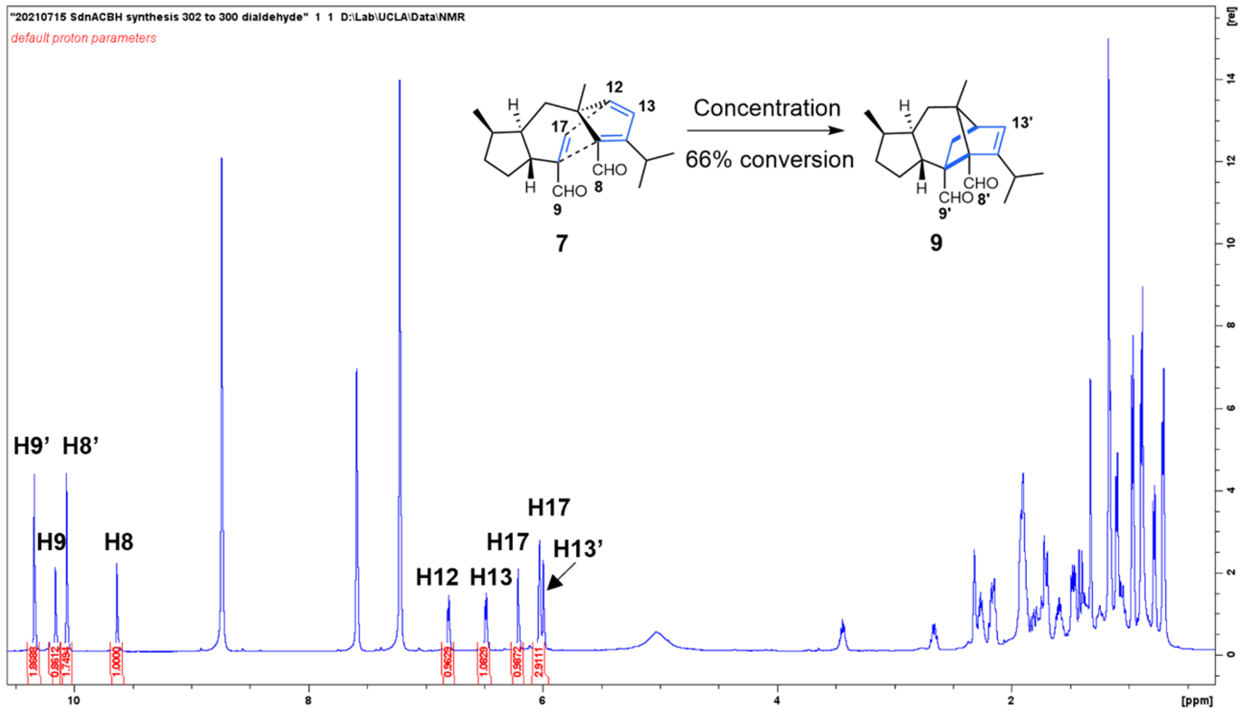

Supplementary Fig 51. ^{1}H NMR of mixed compounds **7** and **9** in d5-pyridine, 500 MHz. The spectrum was taken after a pure sample of compound **7** in $CDCl_2$ was dried in vacuum and redissolved in d5-pyridine. Concentration promoted cyclization of **7** to form a 1 to 2 mixture of **7** and **9**.

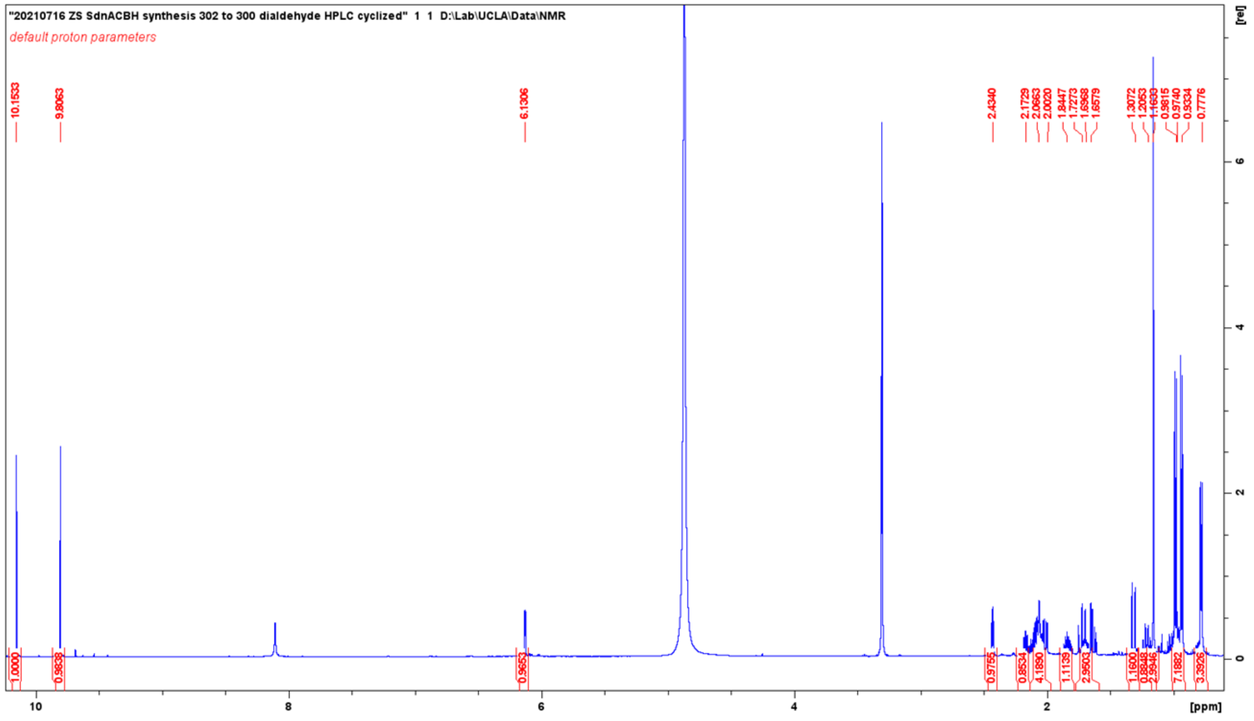

Supplementary Fig 52. ^{1}H NMR of compound **9** in $CDCD_3$, 500 MHz.

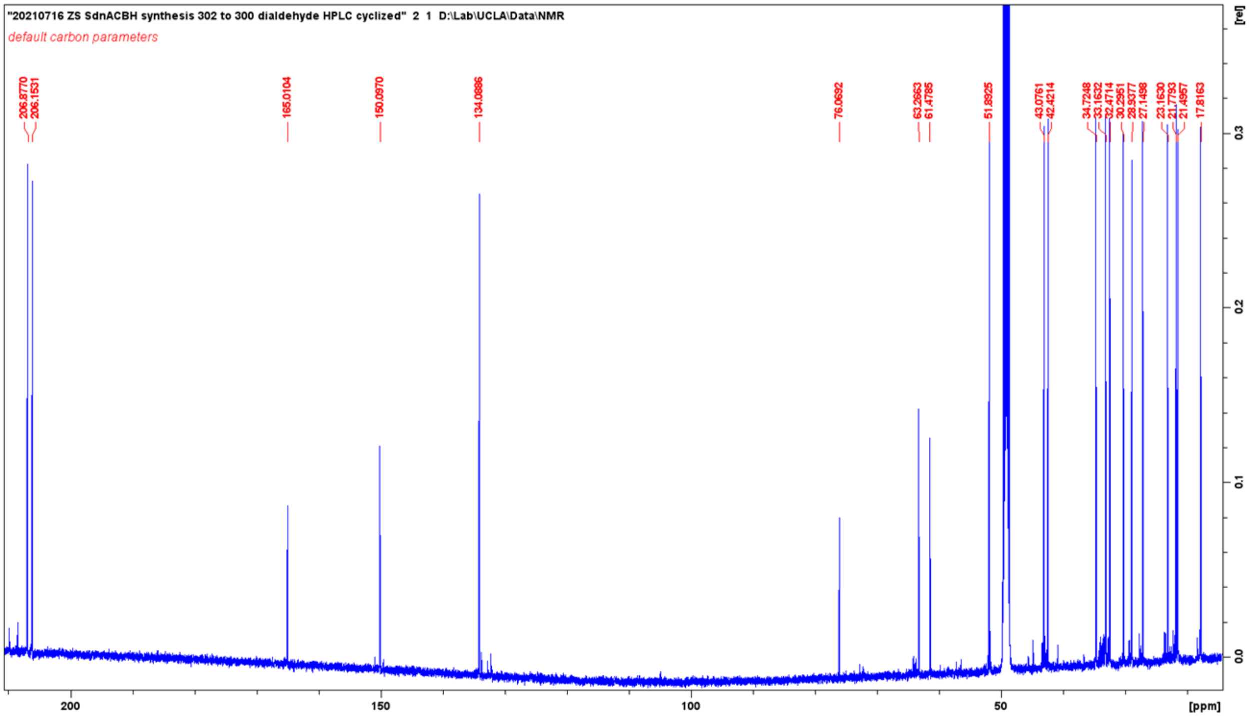

Supplementary Fig 53. ^{13}C NMR of compound **9** in CDCD$_3$, 500 MHz.

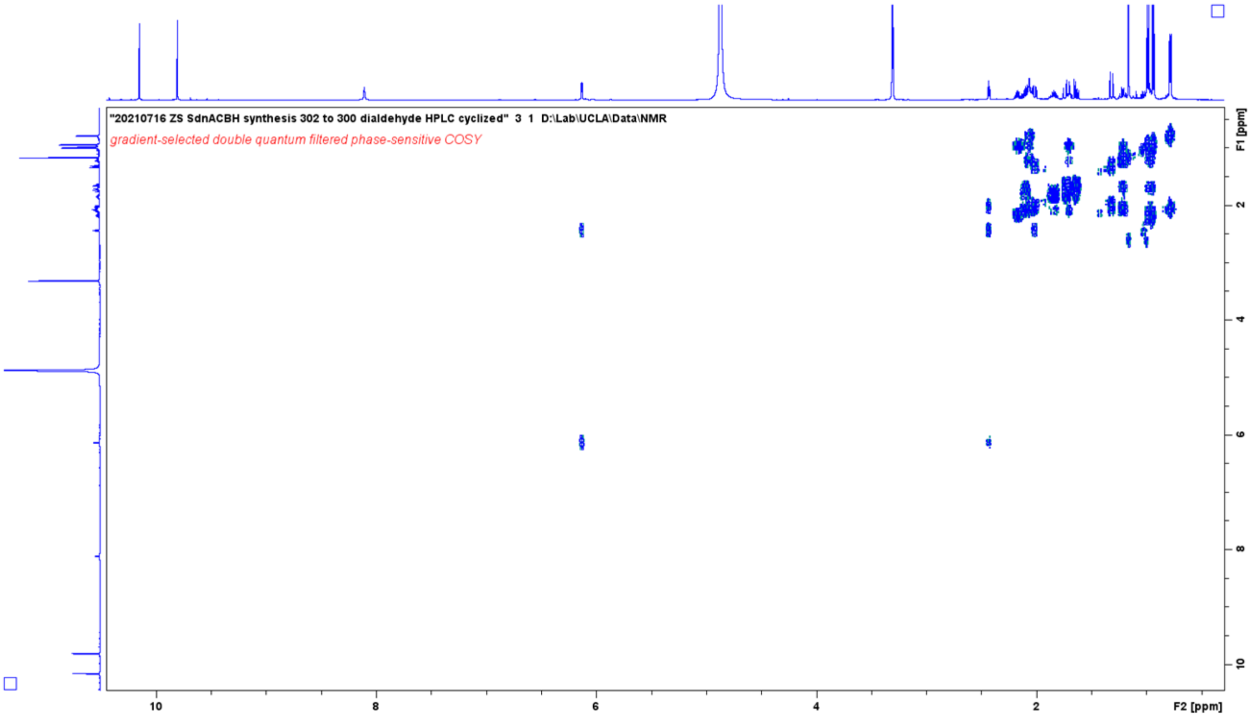

Supplementary Fig 54. ^{1}H-^{1}H COSY of compound **9** in CDCD$_3$, 500 MHz.

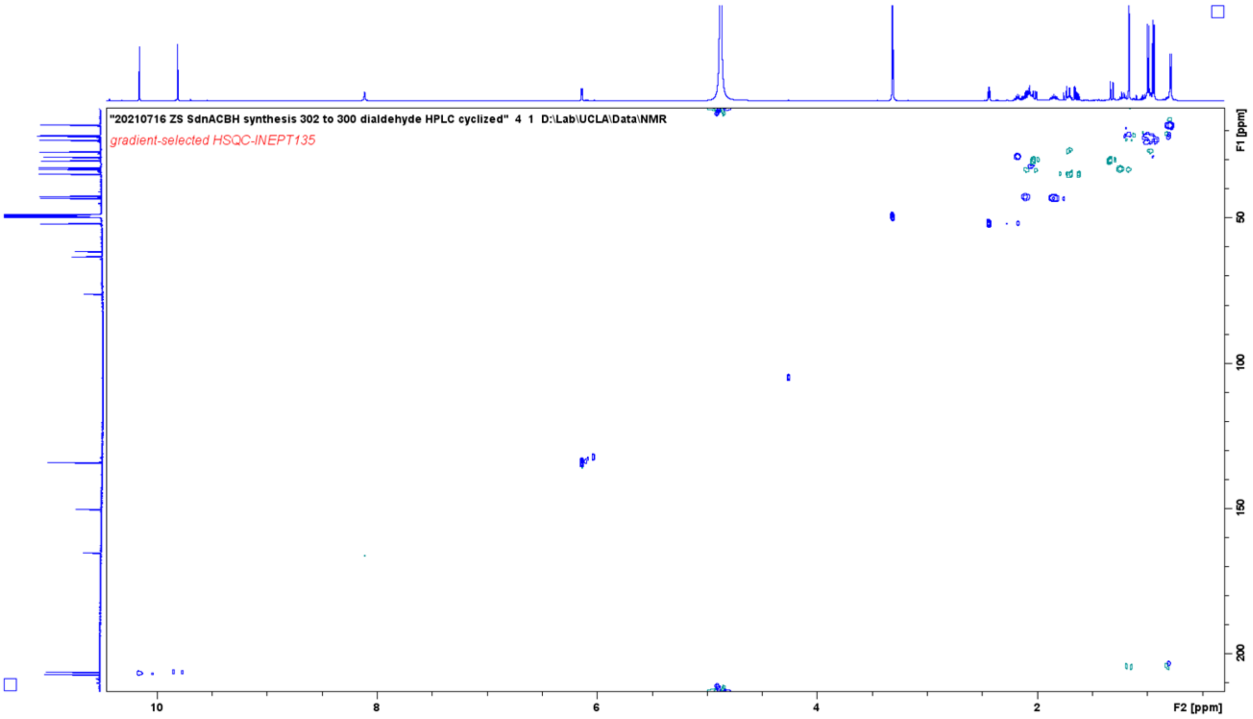

Supplementary Fig 55. ^{1}H-^{13}C HSQC of compound **9** in $CDCD_3$, 500 MHz.

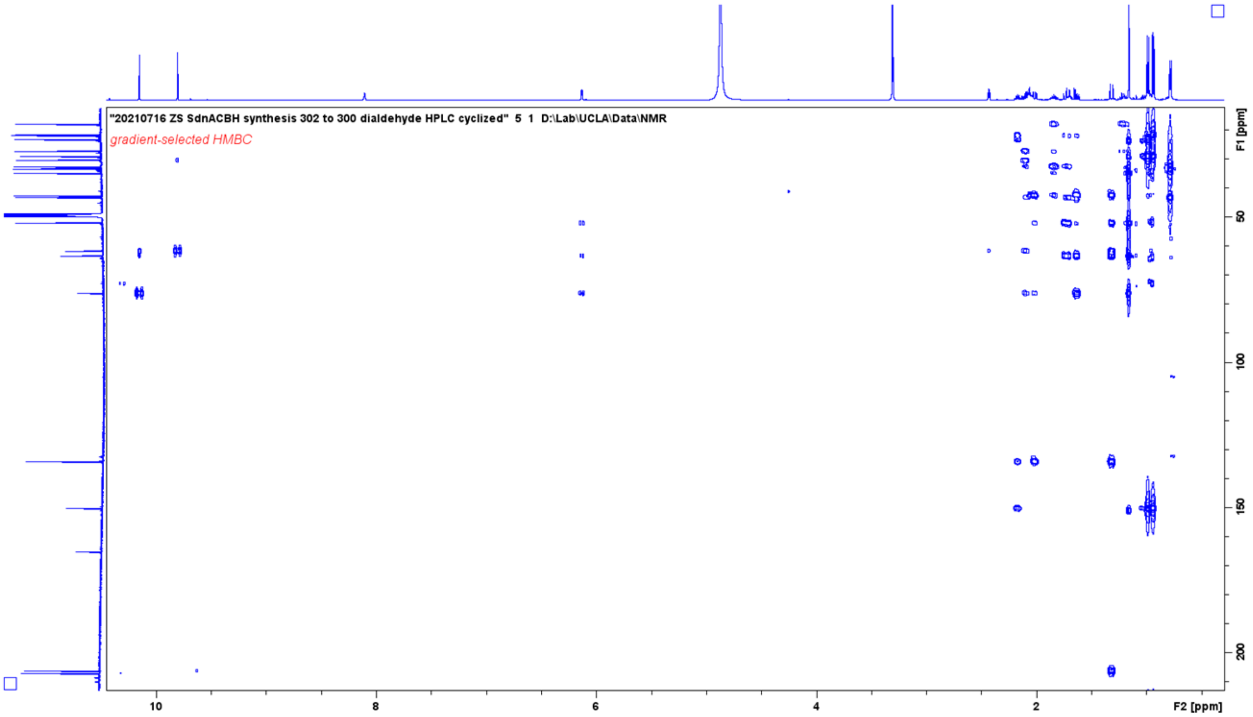

Supplementary Fig 56. ^{1}H-^{13}C HMBC of compound **9** in $CDCD_3$, 500 MHz.

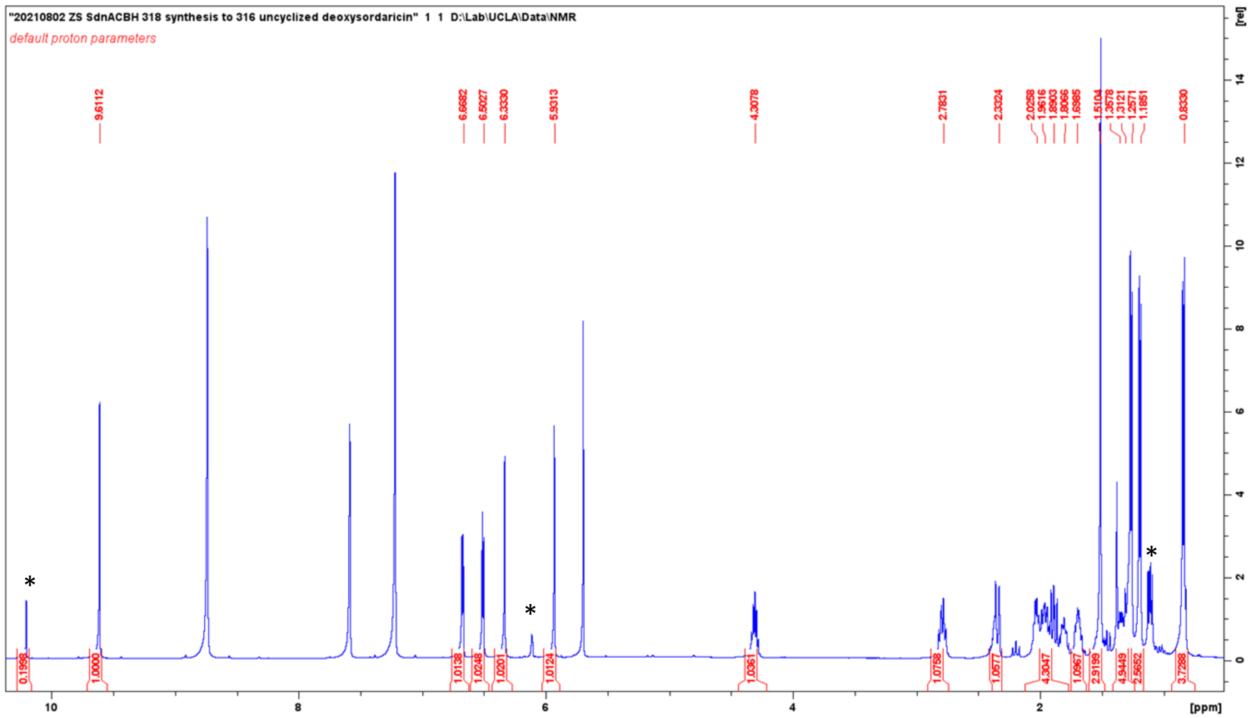

Supplementary Fig 57. ^{1}H NMR of compound **10** in CD_2Cl_2, 500 MHz. Signals labeled with * are from compound **11**, which formed via the cyclization of **10** during the synthesis and reaction workup.

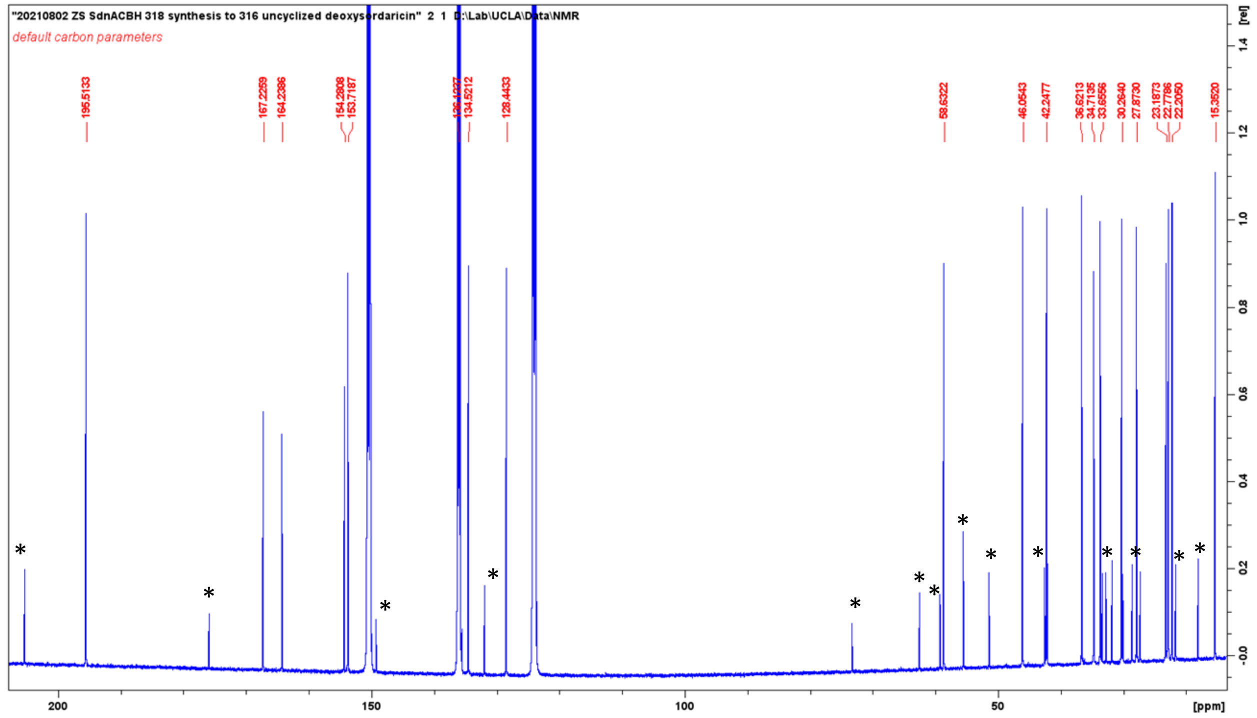

Supplementary Fig 58. ^{13}C NMR of compound **10** in CD_2Cl_2, 500 MHz. Signals labeled with * are from compound **11**, which formed via the cyclization of **10** during the synthesis and reaction workup.

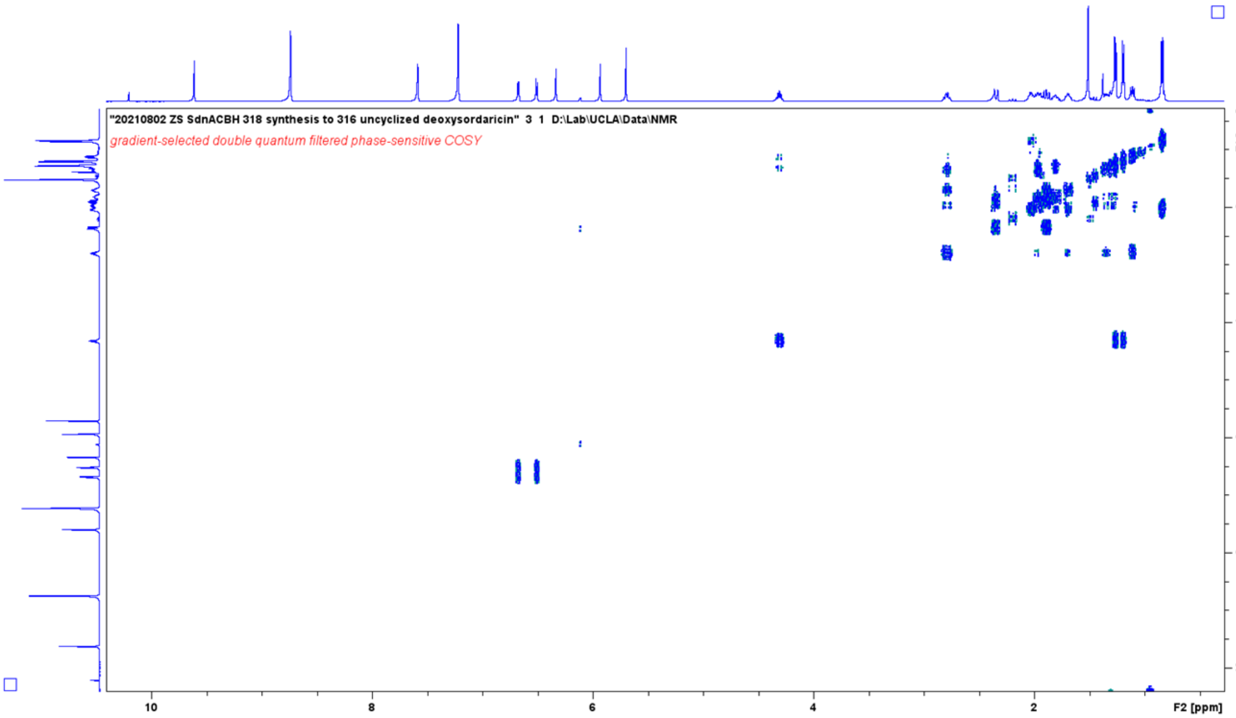

Supplementary Fig 59. ^{1}H-^{1}H COSY of compound **10** in CD_2Cl_2, 500 MHz.

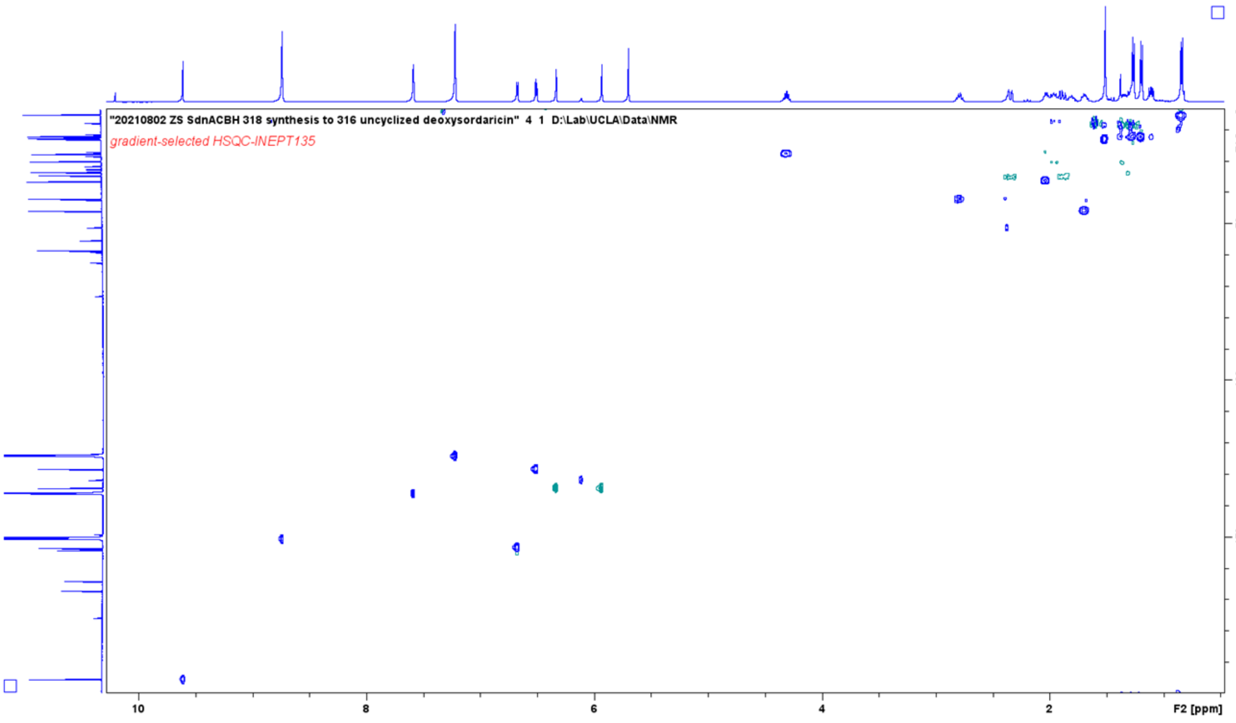

Supplementary Fig 60. ^{1}H-^{13}C HSQC of compound **10** in CD_2Cl_2, 500 MHz.

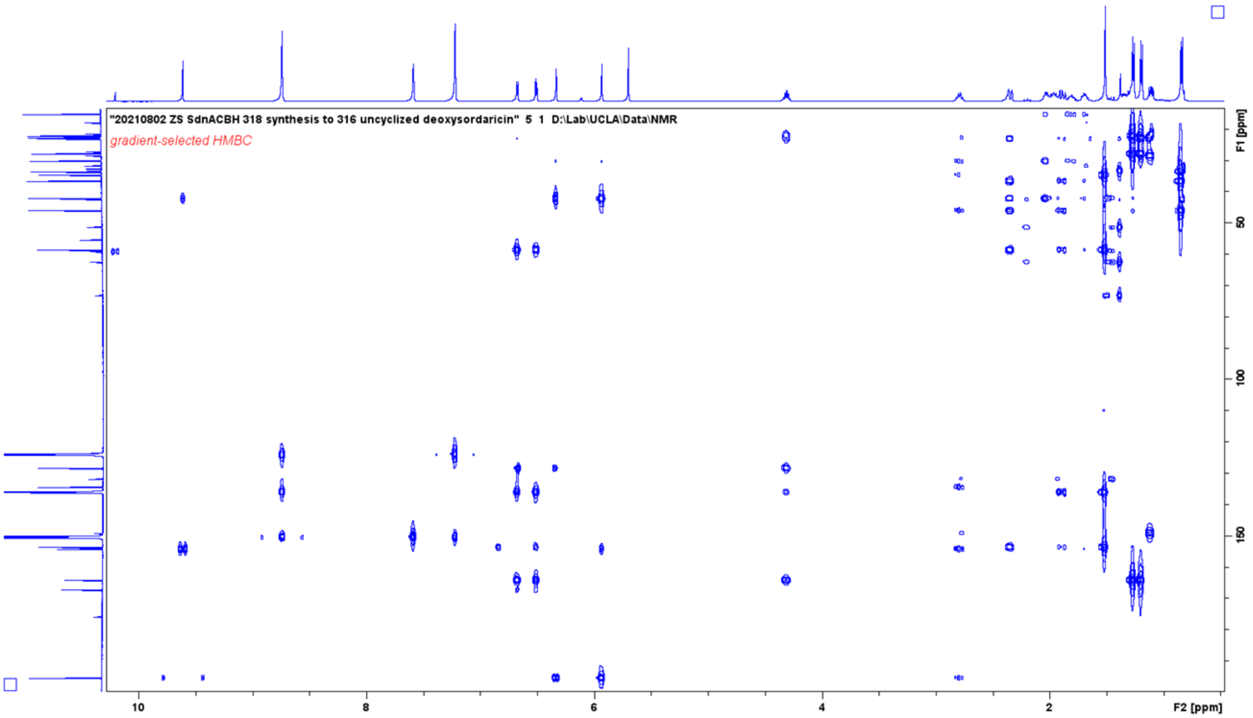

Supplementary Fig 61. ^{1}H-^{13}C HMBC of compound **10** in CD_2Cl_2, 500 MHz.

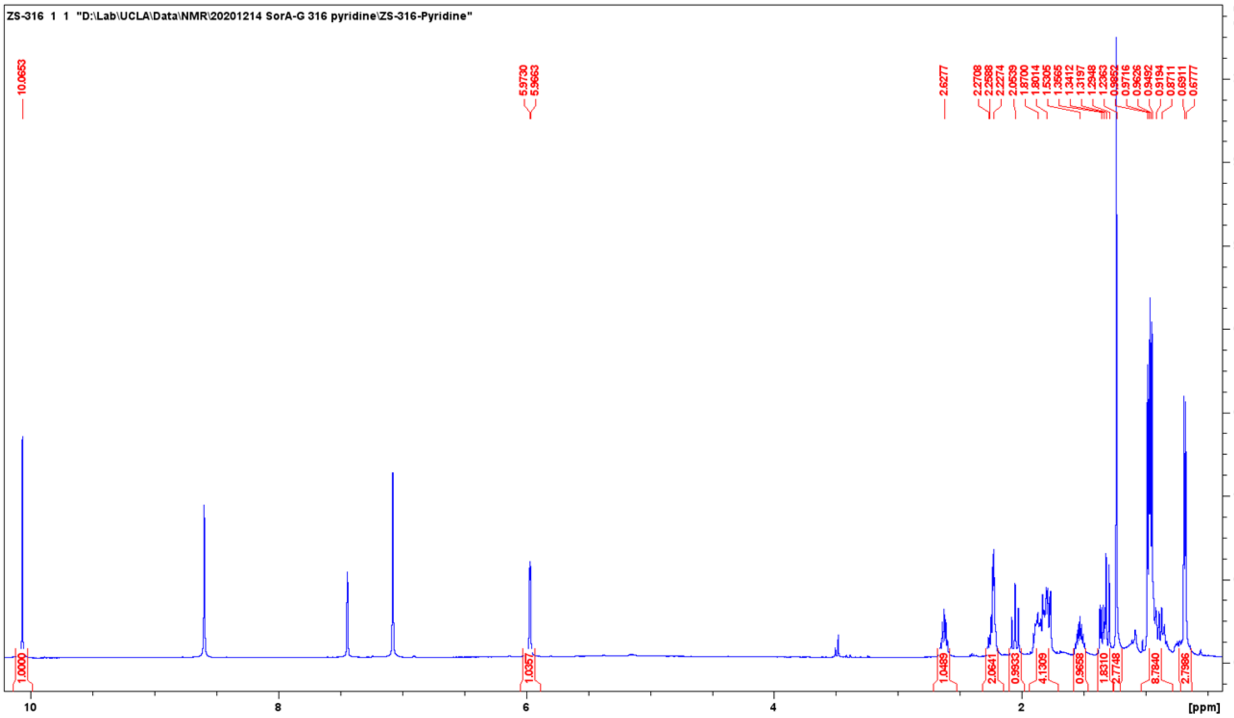

Supplementary Fig 62. ^{1}H NMR of compound **11** in d5-pyridine, 500 MHz.

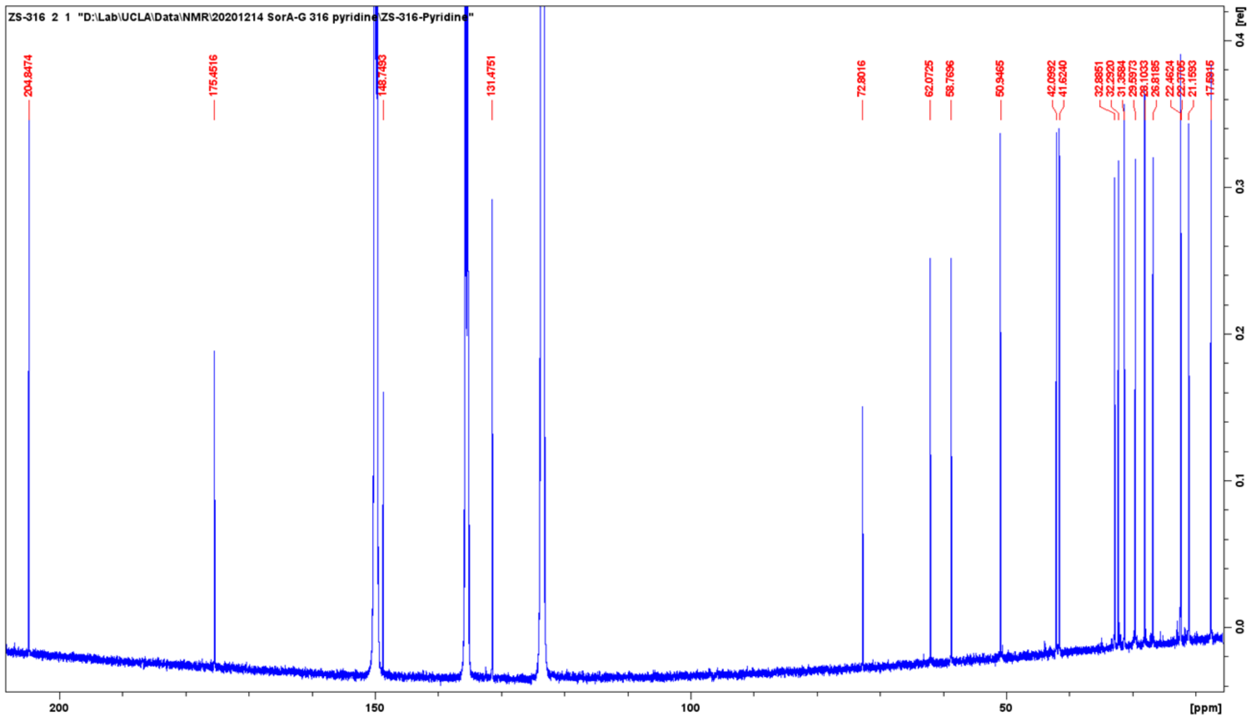

Supplementary Fig 63. ^{13}C NMR of compound **11** in d5-pyridine, 500 MHz.

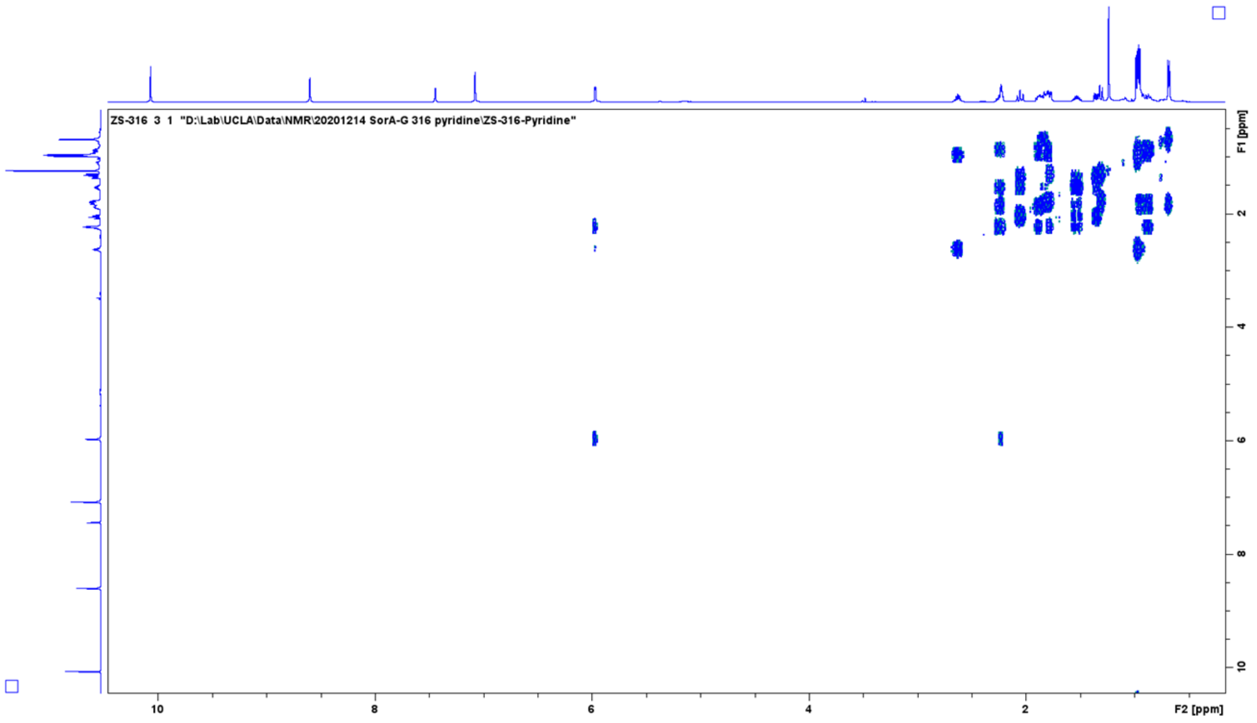

Supplementary Fig 64. ^{1}H-^{1}H COSY of compound **11** in d5-pyridine, 500 MHz.

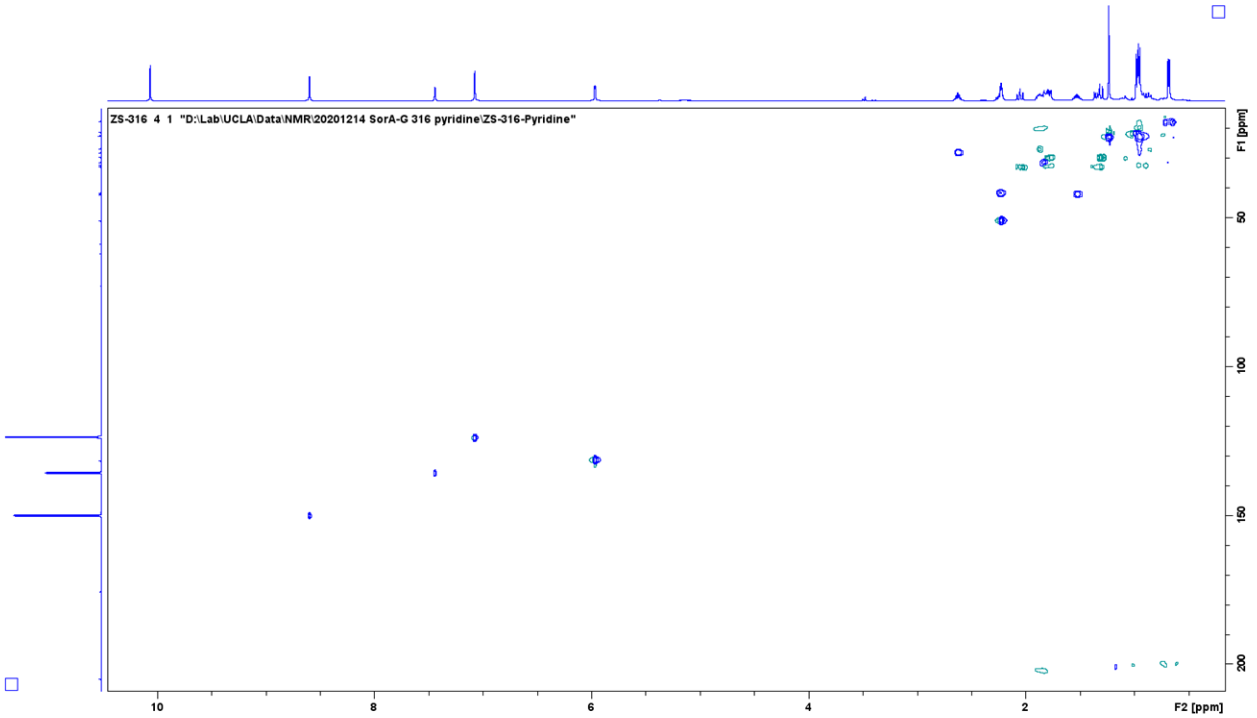

Supplementary Fig 65. ^{1}H-^{13}C HSQC of compound **11** in d5-pyridine, 500 MHz.

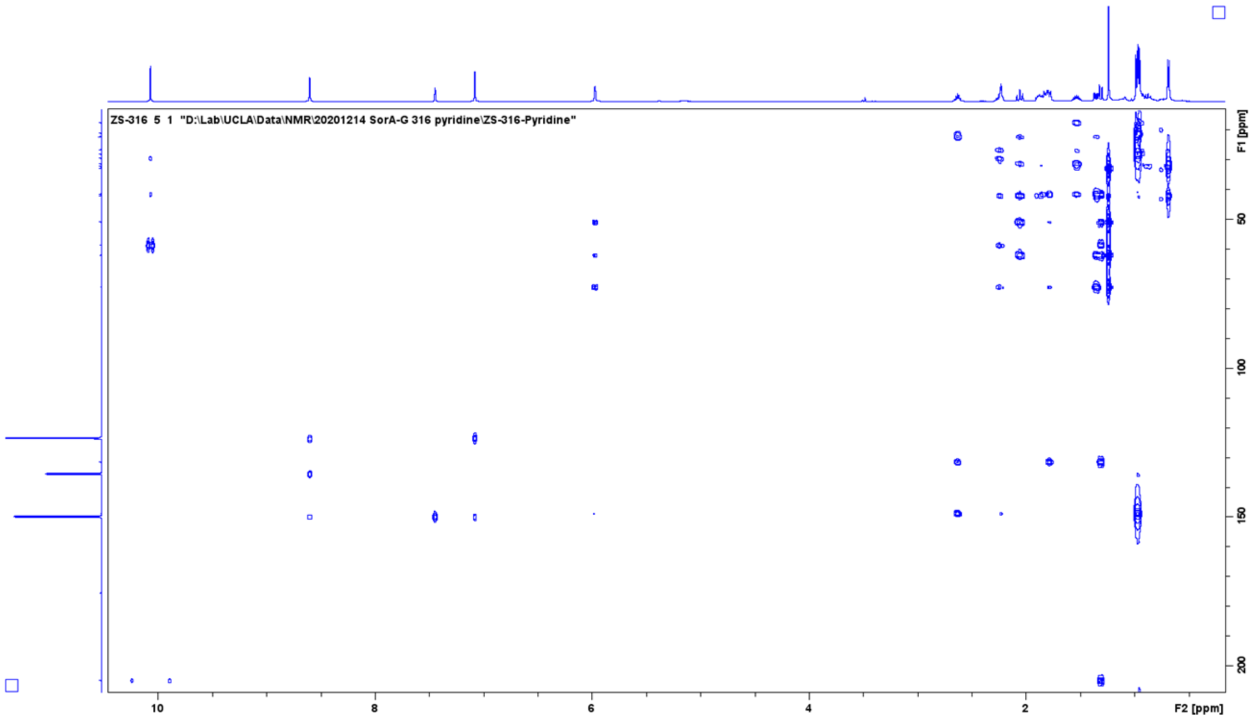

Supplementary Fig 66. ^{1}H-^{13}C HMBC of compound **11** in d5-pyridine, 500 MHz.

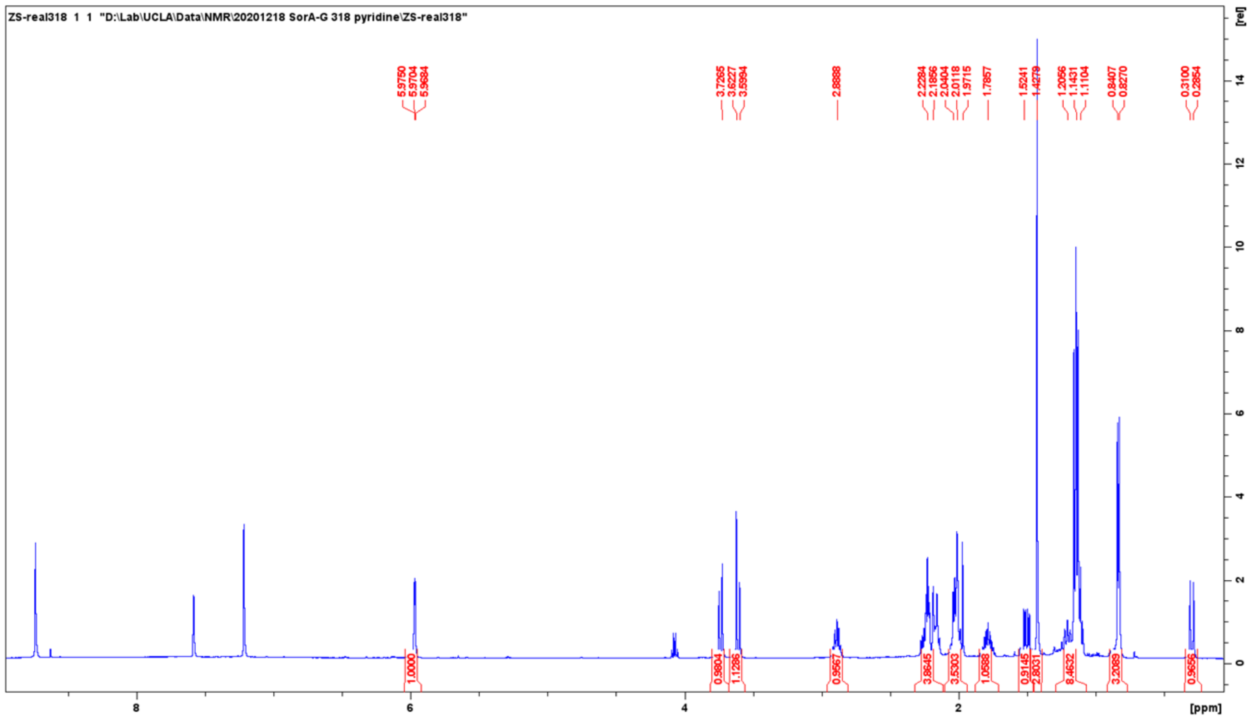

Supplementary Fig 67. ^{1}H NMR of compound **12** in d5-pyridine, 500 MHz.

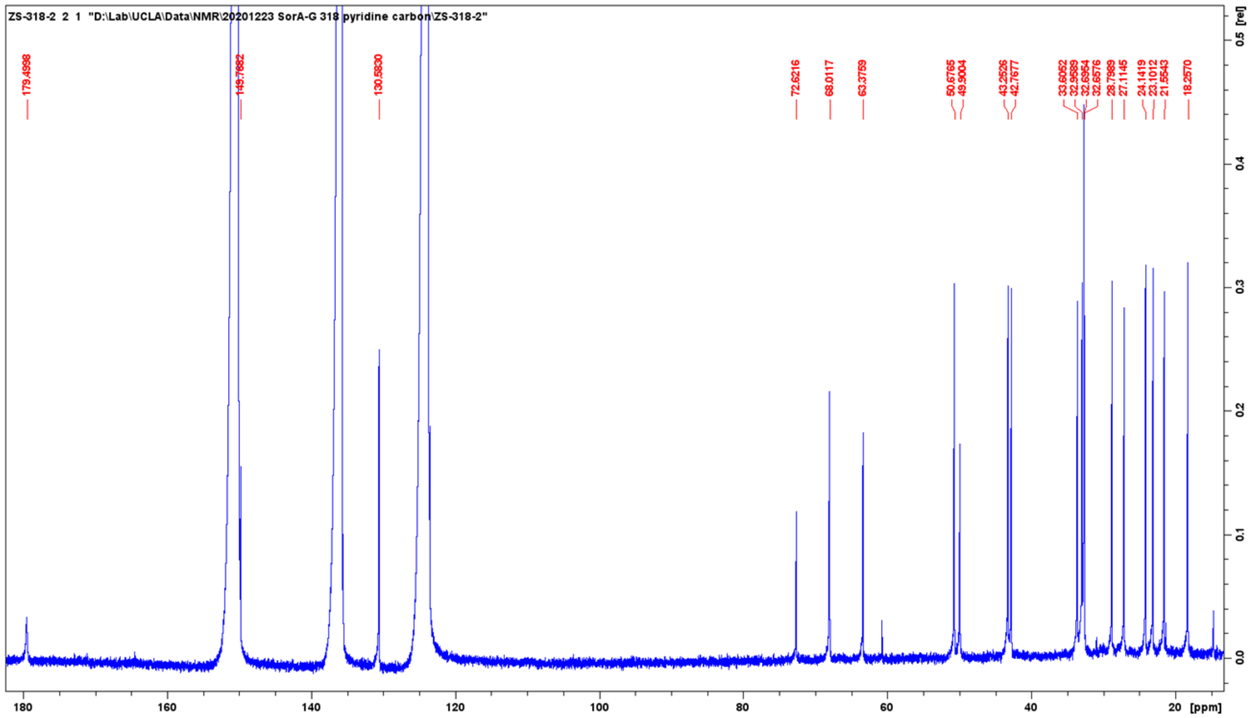

Supplementary Fig 68. ^{13}C NMR of compound **12** in d5-pyridine, 500 MHz.

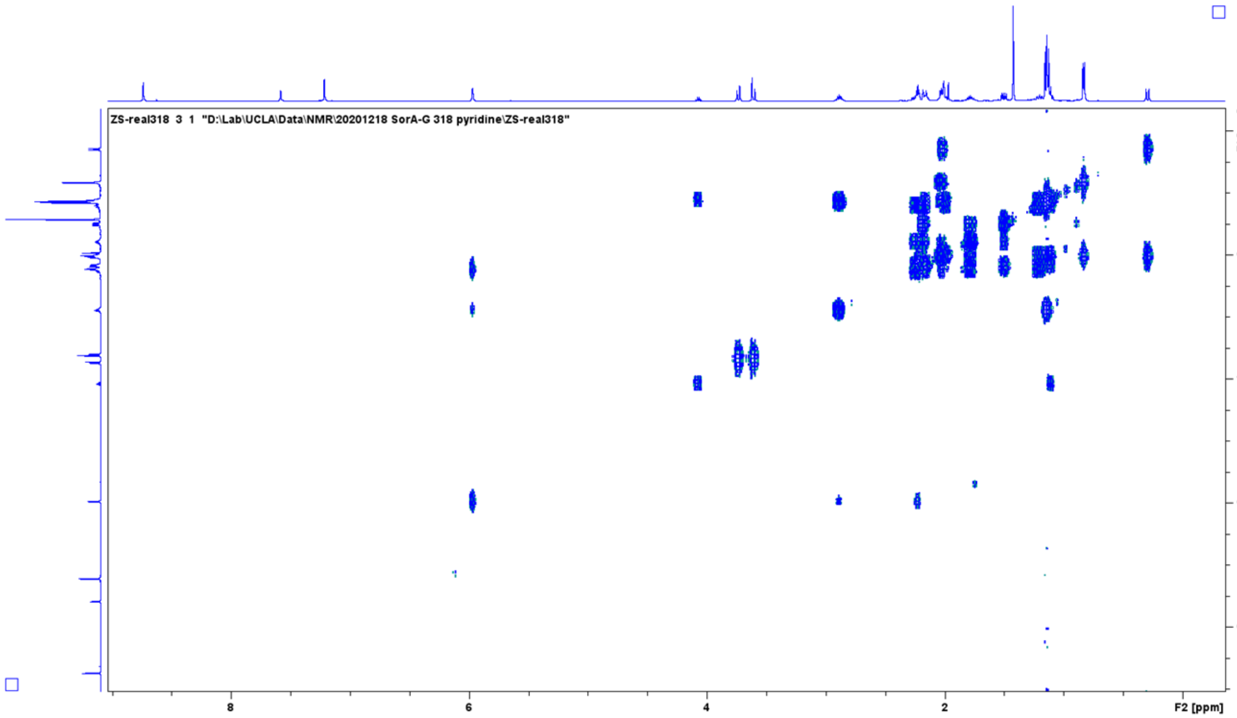

Supplementary Fig 69. ^{1}H-^{1}H COSY of compound **12** in d5-pyridine, 500 MHz.

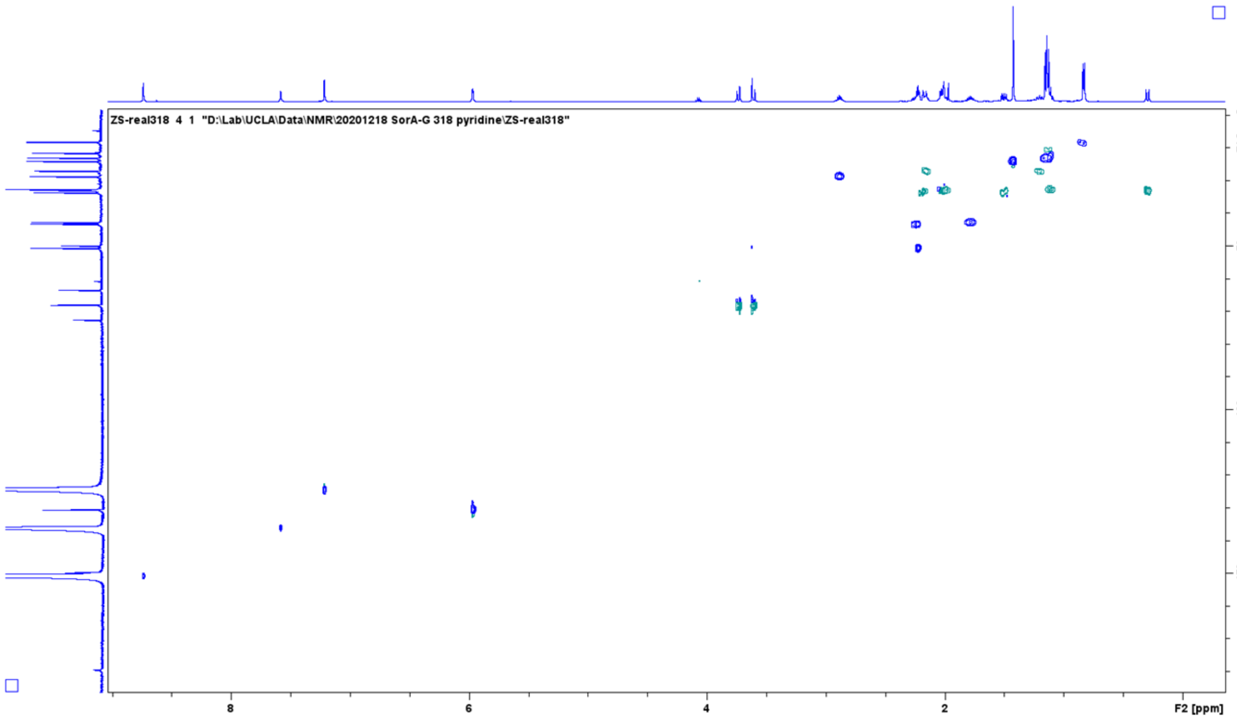

Supplementary Fig 70. ^{1}H-^{13}HSQC of compound **12** in d5-pyridine, 500 MHz.

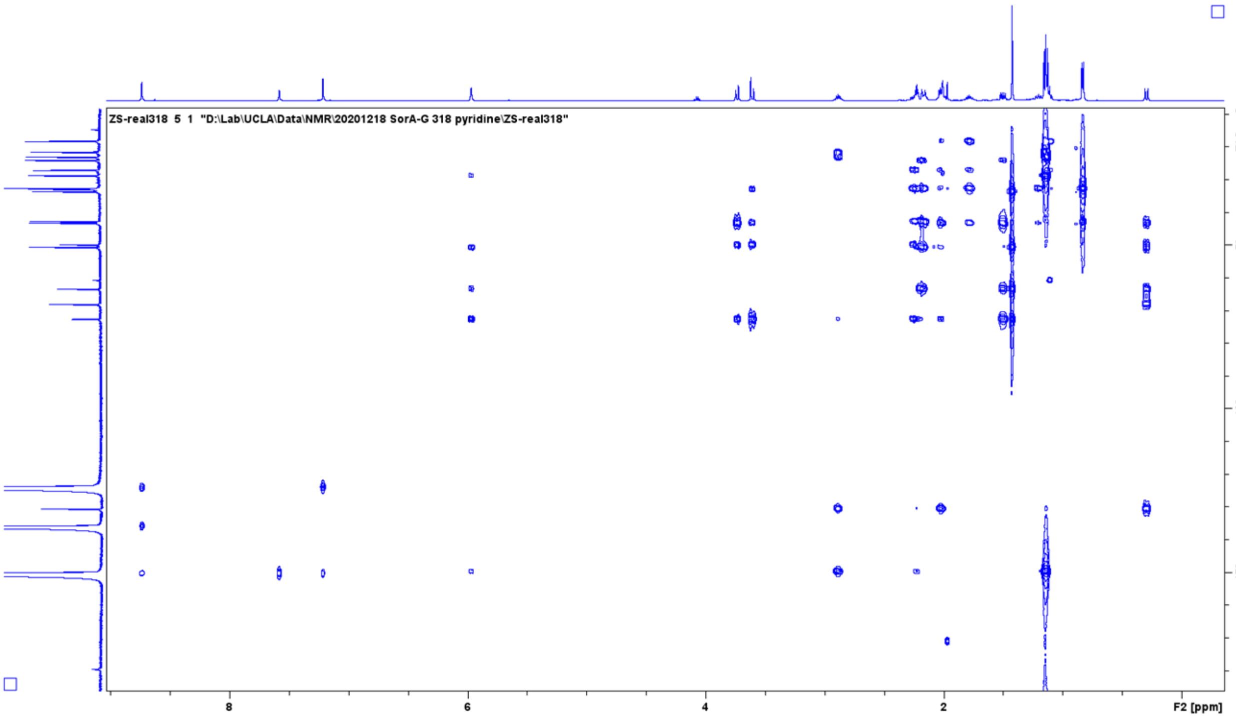

Supplementary Fig 71. ^{1}H-^{13}HMBC of compound **12** in d5-pyridine, 500 MHz.

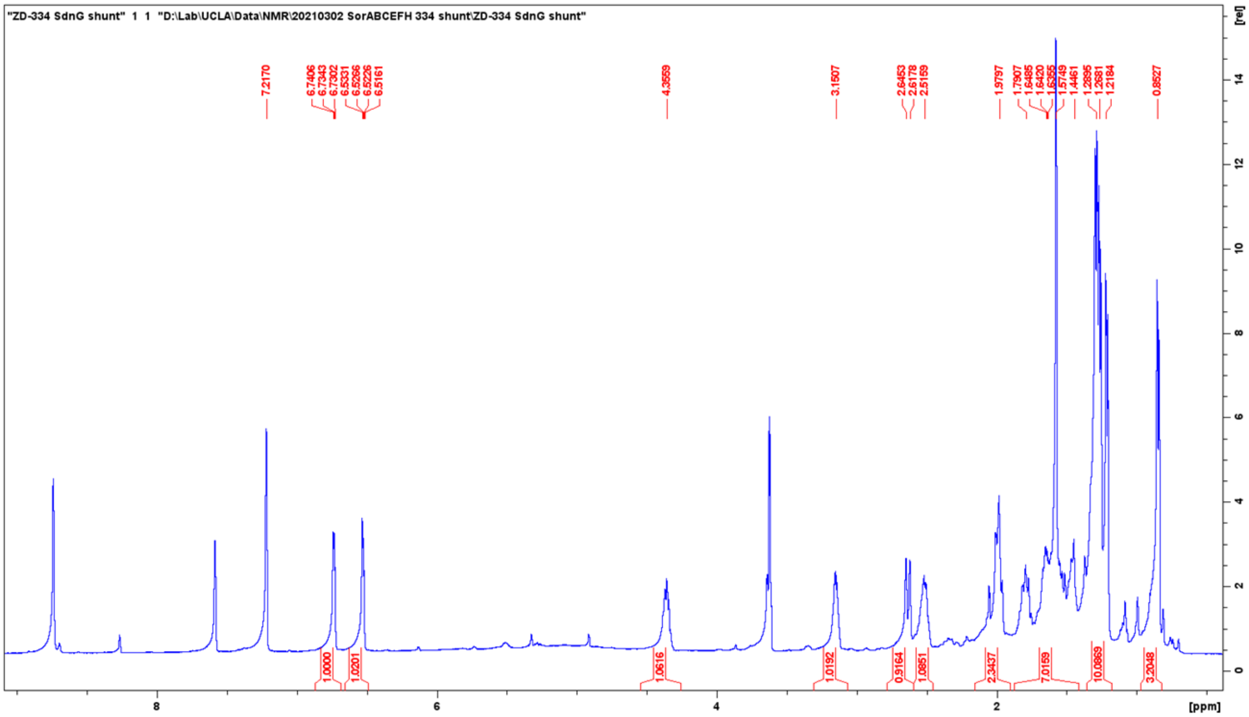

Supplementary Fig 72. ^{1}H NMR of compound **13** in d5-pyridine, 500 MHz.

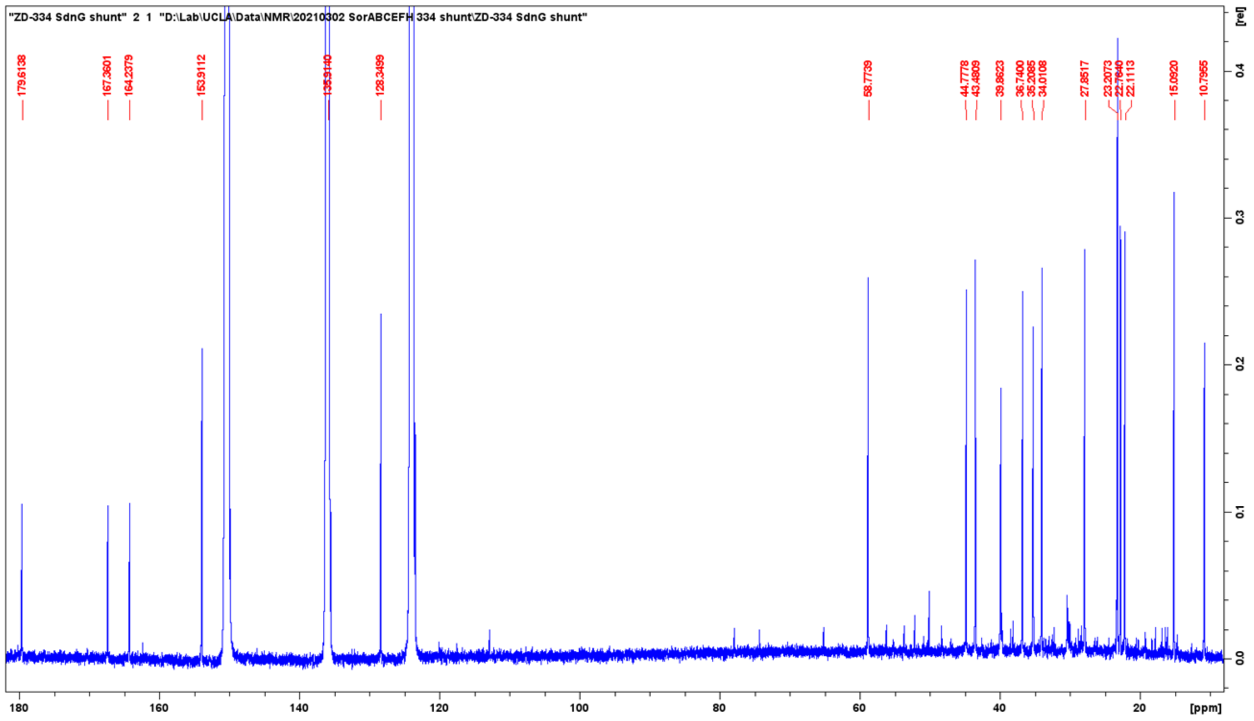

Supplementary Fig 73. ^{13}C NMR of compound **13** in d5-pyridine, 500 MHz.

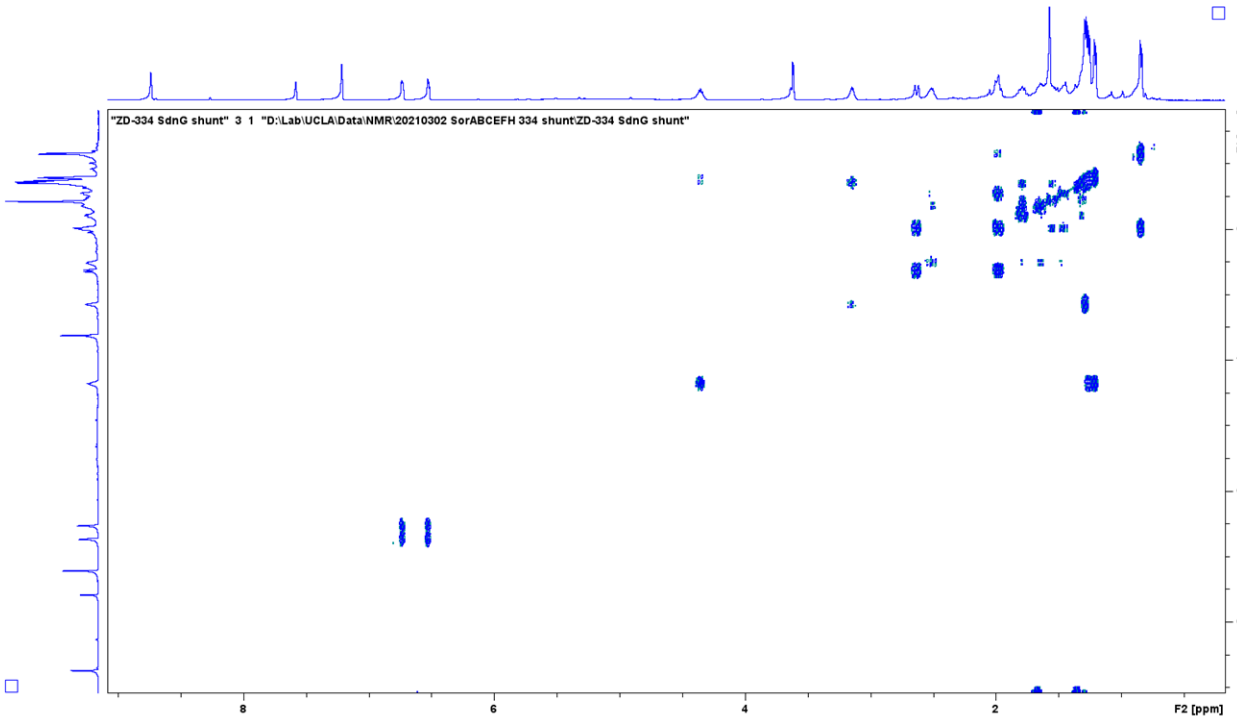

Supplementary Fig 74. ^{1}H-^{1}H COSY of compound **13** in d5-pyridine, 500 MHz.

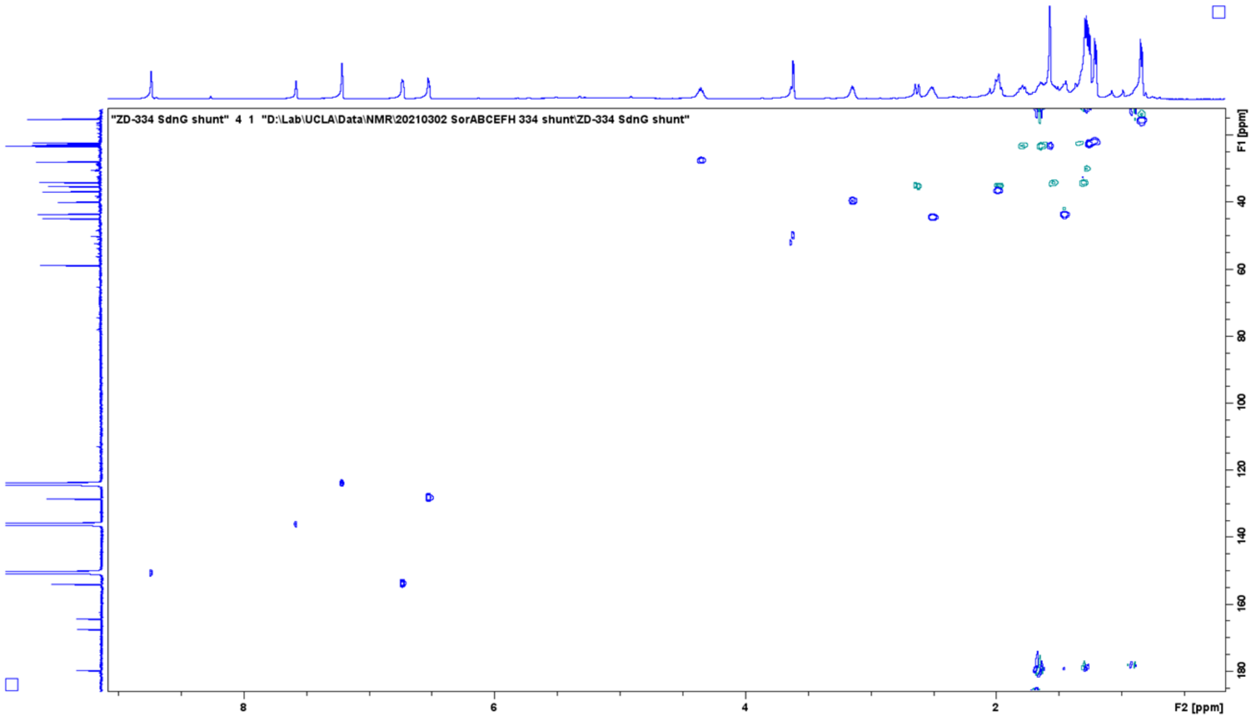

Supplementary Fig 75. ^{1}H-^{13}C HSQC of compound **13** in d5-pyridine, 500 MHz.

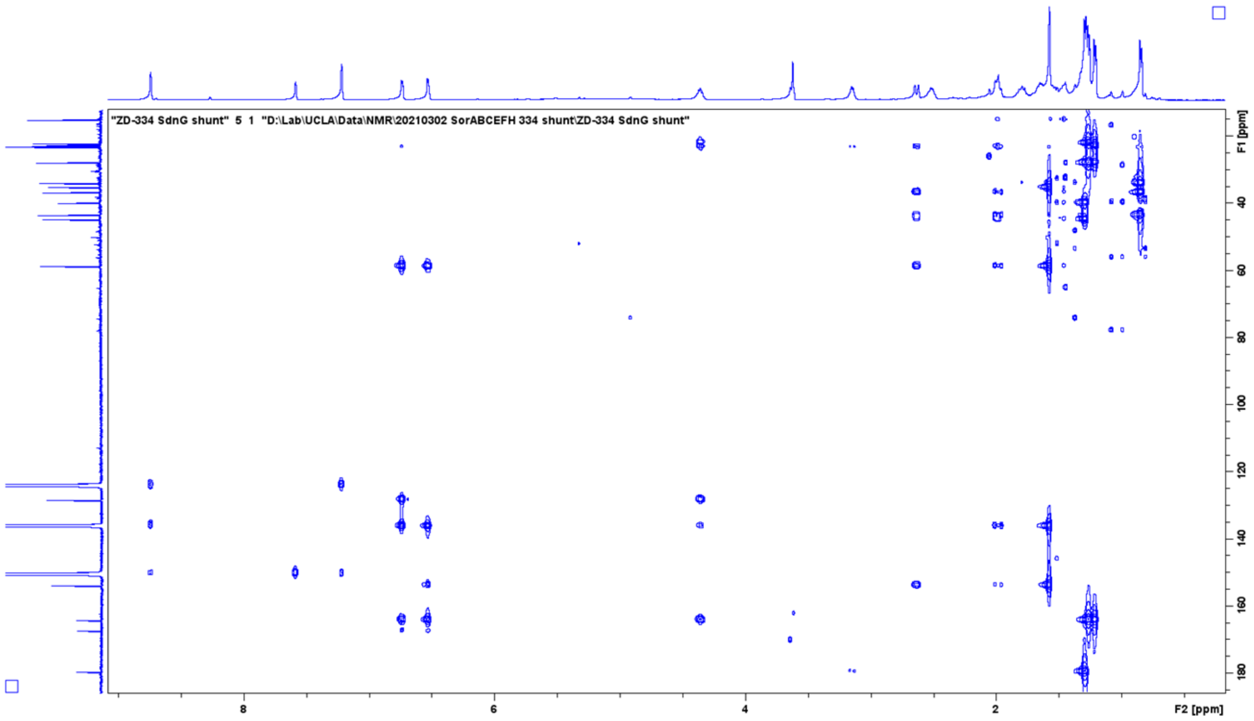

Supplementary Fig 76. ^{1}H-^{13}C HSQC of compound **13** in d5-pyridine, 500 MHz.

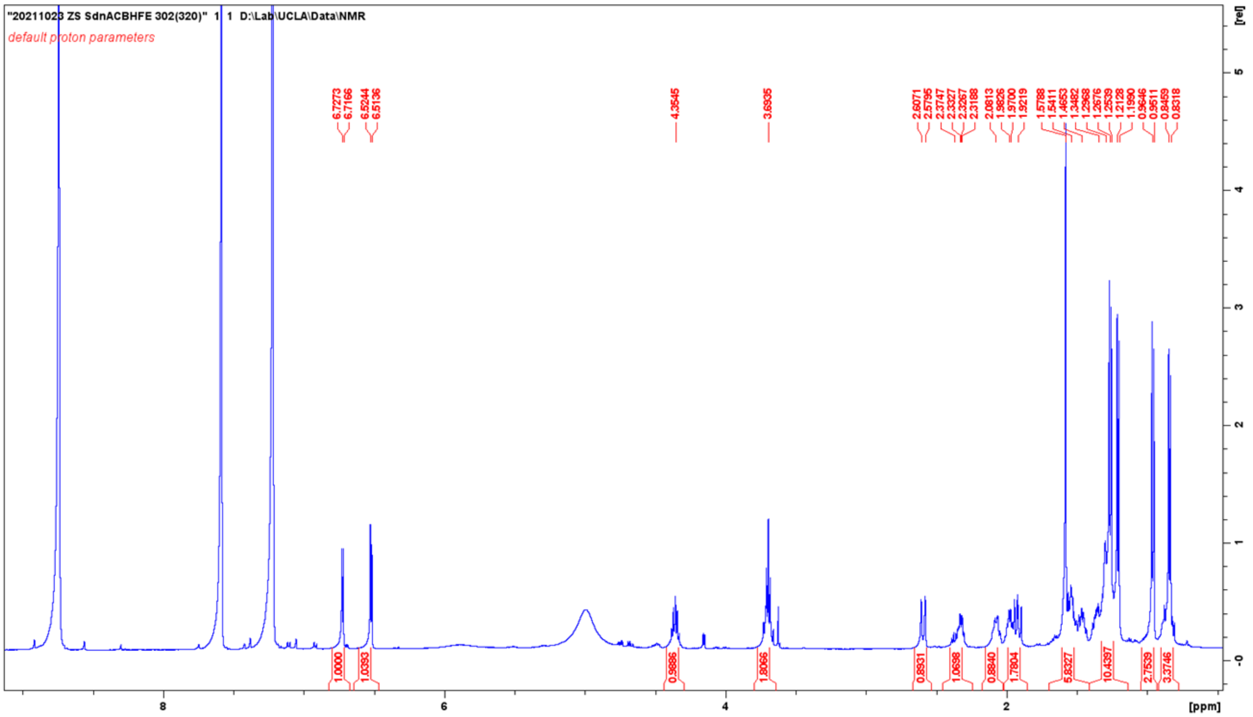

Supplementary Fig 77. ^{1}H NMR of compound **14** in d5-pyridine, 500 MHz.

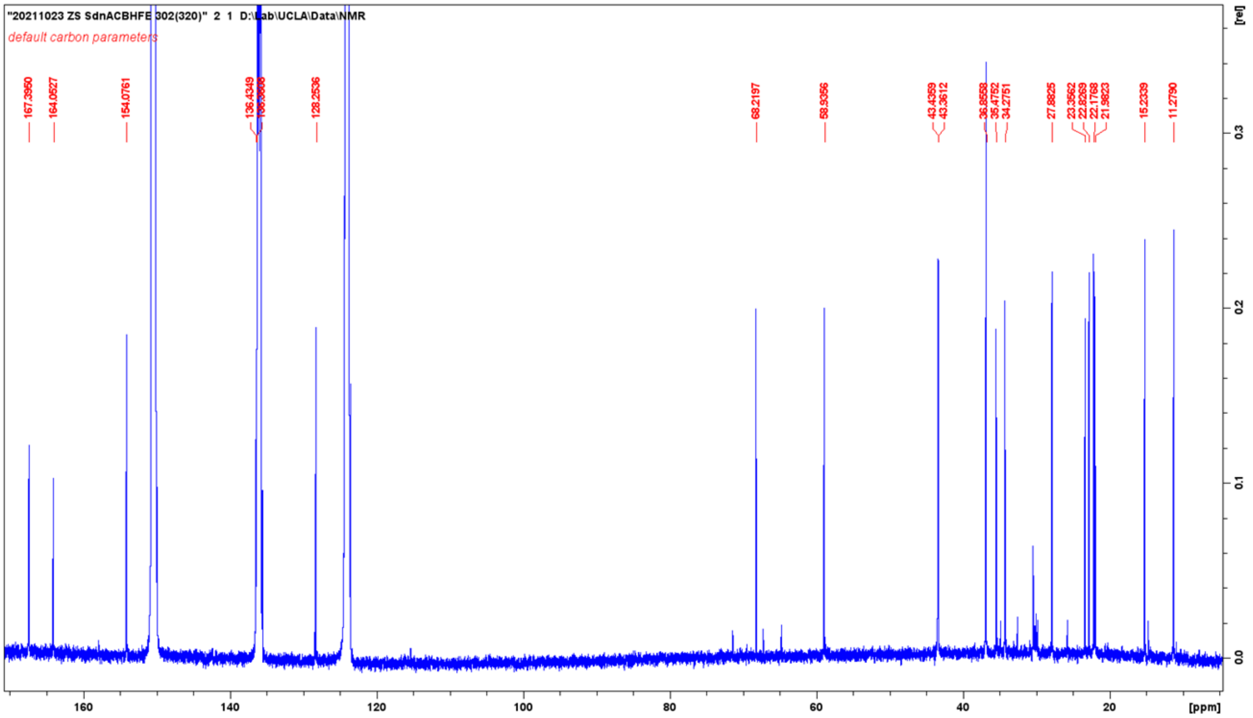

Supplementary Fig 78. ^{13}C NMR of compound **14** in d5-pyridine, 500 MHz.

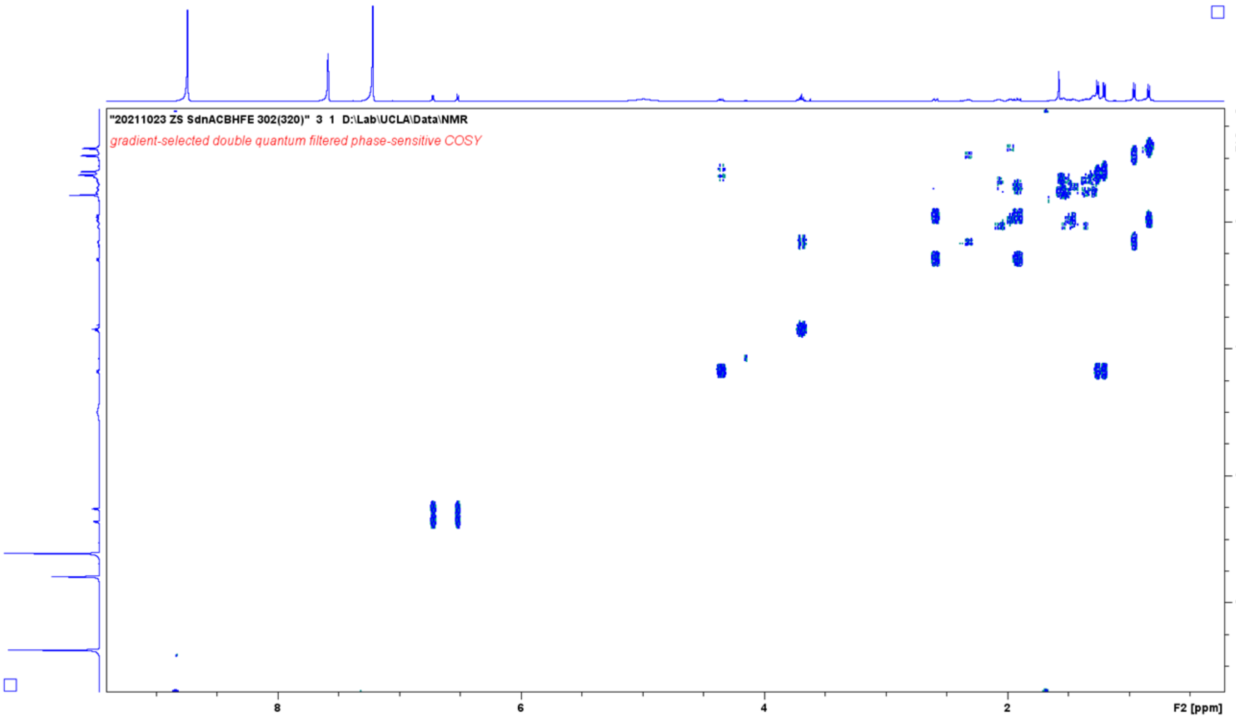

Supplementary Fig 79. ^{1}H-^{1}H COSY of compound **14** in d5-pyridine, 500 MHz.

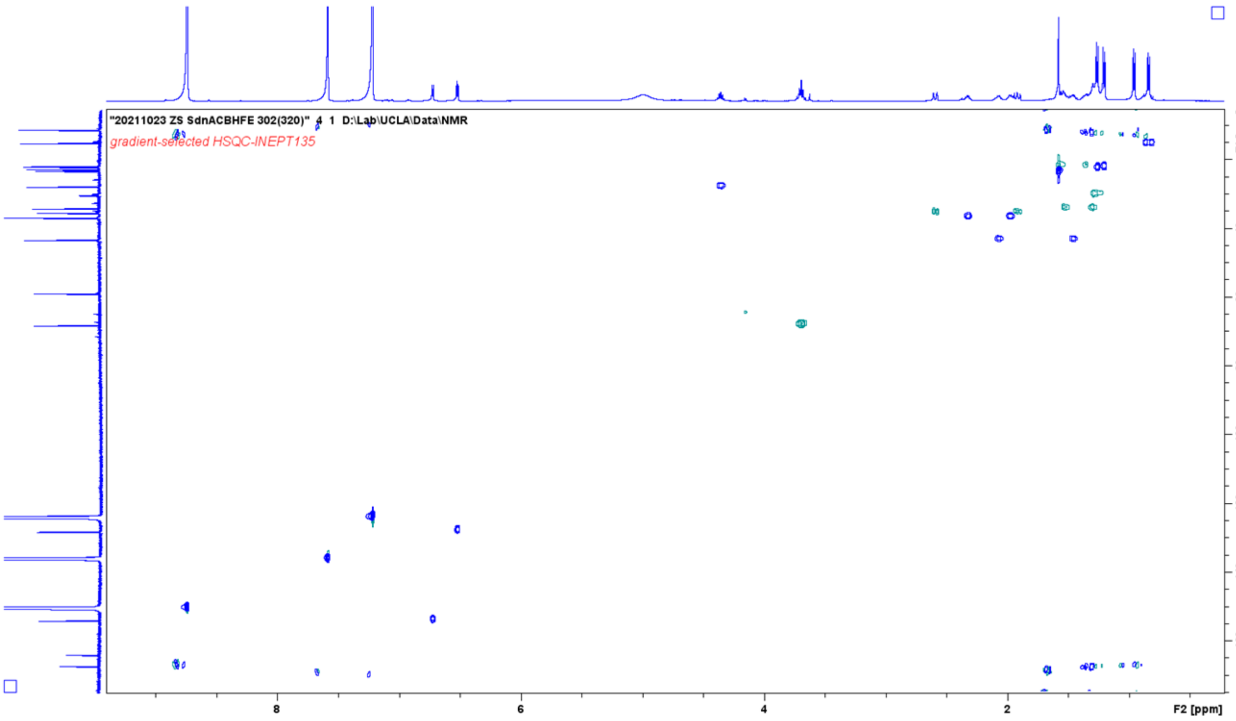

Supplementary Fig 80. ^{1}H-^{13}C HSQC of compound **14** in d5-pyridine, 500 MHz.

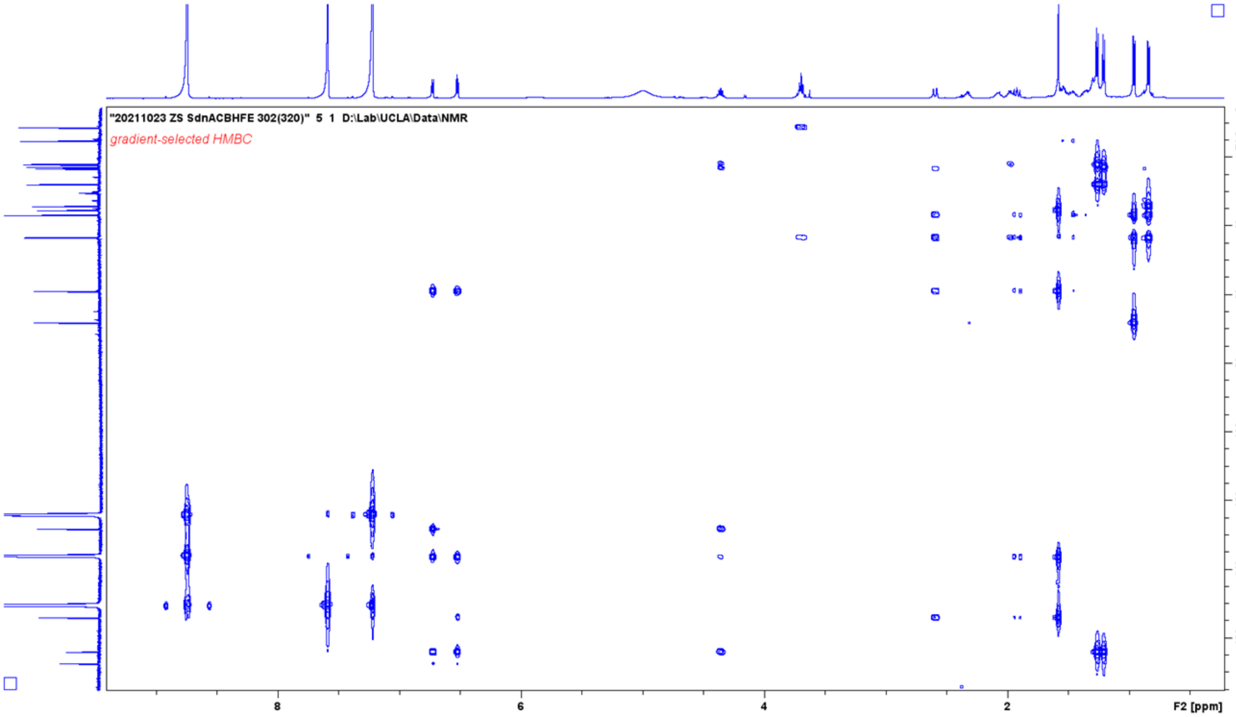

Supplementary Fig 81. ^{1}H-^{13}C HMBC of compound **14** in d5-pyridine, 500 MHz.

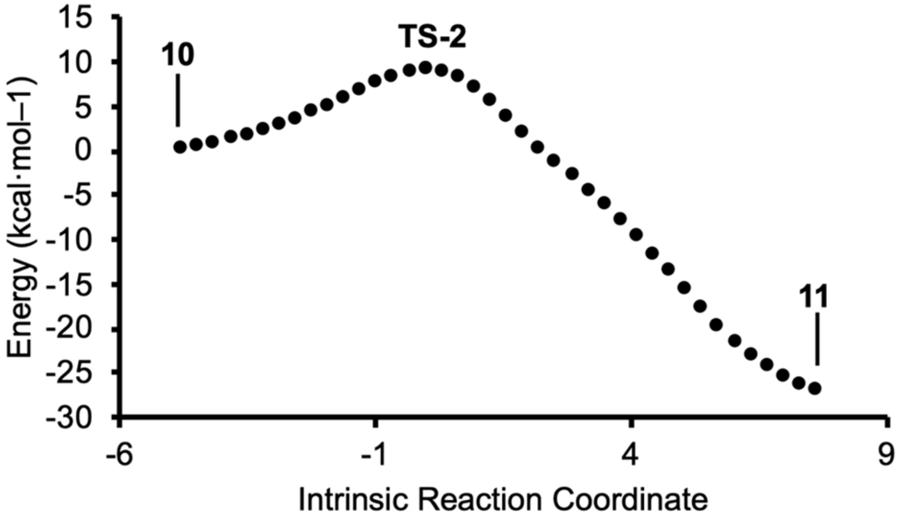

Supplementary Fig 82. Intrinsic reaction coordinate calculation initiated from TS-2.

IV Supplementary references

1. Mander, L. N. & Thomson, R. J. Total Synthesis of Sordaricin. *J. Org. Chem.* **70**, 1654–1670 (2005).

2. Gao, S.-S. *et al.* Biosynthesis of Heptacyclic Duclauxins Requires Extensive Redox Modifications of the Phenalenone Aromatic Polyketide. *J. Am. Chem. Soc.* **140**, 6991–6997 (2018).

3. Jenny, L. Ph.D.Thesis. ETH Zurich, No. 10920. (1994).

4. Kudo, F., Matsuura, Y., Hayashi, T., Fukushima, M. & Eguchi, T. Genome mining of the sordarin biosynthetic gene cluster from Sordaria araneosa Cain ATCC 36386: characterization of cycloaraneosene synthase and GDP-6-deoxyaltrose transferase. *J. Antibiot. (Tokyo).* **69**, 541–548 (2016).

5. Meunier, B., de Visser, S. P. & Shaik, S. Mechanism of Oxidation Reactions Catalyzed by Cytochrome P450 Enzymes. *Chem. Rev.* **104**, 3947–3980 (2004).

6. Guengerich, F. P. Mechanisms of Cytochrome P450-Catalyzed Oxidations. *ACS Catal.* **8**, 10964–10976 (2018).

V Uncropped scan of SDS-PAGE in Supplementary Fig 8.

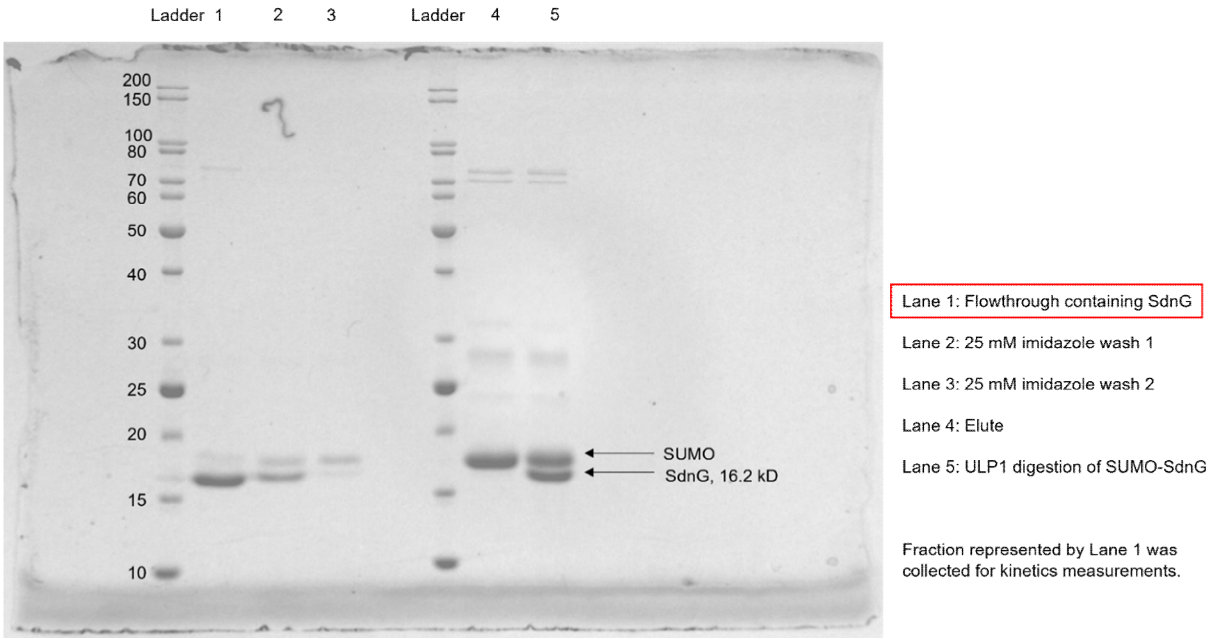

Supplement: Supplementary file 1 — Supplementary Information [file 41467_2022_30288_MOESM1_ESM.pdf]
