# Supplementary material for: Discovery and characterization of a terpene biosynthetic pathway featuring a norbornene-forming Diels-Alderase
Source: Nat Commun. 2022 May 11;13:2568. doi: 10.1038/s41467-022-30288-6 (PMC9095873; doi:10.1038/s41467-022-30288-6)
Supplement: Supplementary file 3 — Description of Additional Supplementary Information [file 41467_2022_30288_MOESM3_ESM.pdf]

Description of Additional Supplementary Information

The Supplementary Information contains a “Supplementary Information” file (including Supplementary Figures, Supplementary Tables, and Supplementary Notes), a “Supplementary Data-Molecular coordinates of calculated structures” file, and a “Supplementary Table-Sequences of oligonucleotides used in the study” file.
